# Transforming Growth Factor‐β Pathway: Biological Functions and Therapeutic Targets

**DOI:** 10.1002/mco2.70278

**Published:** 2025-06-27

**Authors:** Reham Hassan Mekky, Mohammed E. Abo‐El Fetoh, Safaa A. Faheem, Abdullah F. Radwan, Mariam H. Fawzy, Aya M. Mustafa, Mohamed A. Said, Daniela Calina, Javad Sharifi‐Rad, William C. Cho

**Affiliations:** ^1^ Department of Pharmacognosy Faculty of Pharmacy Egyptian Russian University Badr City Cairo Egypt; ^2^ Department of Pharmacology and Toxicology Faculty of Pharmacy Egyptian Russian University Badr City Cairo Egypt; ^3^ Department of Biochemistry and Molecular Biology Faculty of Pharmacy Egyptian Russian University Badr City Cairo Egypt; ^4^ Department of Pharmaceutical Chemistry Faculty of Pharmacy Egyptian Russian University Badr City Cairo Egypt; ^5^ Department of Clinical Pharmacy University of Medicine and Pharmacy of Craiova Craiova Romania; ^6^ Universidad Espíritu Santo Samborondón Ecuador; ^7^ Centro de Estudios Tecnológicos y Universitarios del Golfo Veracruz Mexico; ^8^ Department of Medicine College of Medicine Korea University Seoul Republic of Korea; ^9^ Department of Clinical Oncology Queen Elizabeth Hospital Kowloon Hong Kong SAR China

**Keywords:** angiogenesis, apoptosis, cancer progression, natural compounds, TGF‐β pathway, therapeutic modulation

## Abstract

Cancer progression is often driven by aberrant cell growth and genetic mutations, leading to metastasis. The transforming growth factor‐beta (TGF‐β) pathway, a key regulator of cellular growth and differentiation, exhibits dual roles in cancer by initially acting as a tumor suppressor and later promoting tumor progression and metastasis. Natural compounds, recognized for their diverse bioactivities and low toxicity, have shown potential in targeting cancer‐related pathways, including TGF‐β signaling. This review examines the therapeutic potential of natural products in modulating TGF‐β signaling and their anticancer effects across various cancer types. We evaluated relevant preclinical and clinical studies assessing the impact of natural products on TGF‐β modulation and cancer progression. Natural compounds from sources such as plants impact TGF‐β signaling, influencing processes like cell proliferation, apoptosis, and angiogenesis. Key compounds reviewed include ginsenosides, halofuginone, and epigallocatechin gallate, demonstrating significant anticancer activity via TGF‐β pathway modulation. These findings suggest natural products may serve as complementary therapies in cancer treatment by targeting TGF‐β signaling, potentially improving patient outcomes. Continued research and clinical evaluation are necessary to integrate these compounds into conventional cancer therapies, aiming to offer safer, cost‐effective options that enhance quality of life.

## Graphical abstract

Natural compounds modulate the TGF‐β pathway, which acts as both a tumor suppressor and promoter during cancer progression. Agents such as ginsenosides, halofuginone, and EGCG influence cell proliferation, apoptosis, and angiogenesis. These bioactive molecules may complement standard therapies, highlighting the need for advanced formulations, mechanistic studies, and clinical validation.

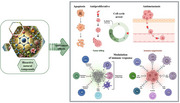

## 1 Introduction

Cancer represents a critical component of the global disease burden, characterized by the unchecked proliferation of abnormal cells due to genetic mutations; these mutations disrupt normal cellular functions, leading to invasion of surrounding tissues and metastasis to distant sites [1, 2]. This disease process contributes extensively to worldwide morbidity and mortality, straining healthcare systems and resources [3]. Addressing cancer's mechanisms and progression is essential for reducing its significant impact on global health [4]. Cancer treatment has relied heavily on approaches such as chemotherapy, radiation, and surgery; however, these treatments often come with significant side effects and limitations, prompting the need for alternative therapies [5, 6, 7, 8]. Transforming growth factor‐β (TGF‐β) is a member of the versatile TGF‐β family of cytokines, which play important roles in regulating a wide range of essential activities, including cellular differentiation, development, invasion, motility, angiogenesis, extracellular matrix (ECM) production, and immune responses [9]; it has other activities beyond regulating the proliferation, differentiation, apoptosis, epithelial–mesenchymal transition (EMT), and metastasis of cancer cells. TGF‐β has been linked in recent publications to its impact on cells within the tumor microenvironment (TME). It stimulates the deposition of ECM, promotes angiogenesis, and suppresses the antitumor immune response [10]. The TGF‐β signaling pathway can either promote or inhibit tumorigenesis, contingent on cellular and environmental contexts; it acts as a tumor suppressor in normal tissues by halting the cell cycle at G1, curbing epithelial cell proliferation, and promoting differentiation, yet in advanced tumors, TGF‐β shifts to an oncogenic role, driving cancer cell growth, self‐renewal of cancer‐initiating cells, EMT, invasion, tumor progression, metastasis, and immune evasion [11, 12, 13]. Furthermore, TGF‐β can induce a favorable TME by stimulating cancer‐associated fibroblasts (CAFs) through paracrine signaling [14]. This activation of CAFs leads to the promotion of angiogenesis, production of ECM, and suppression of the antitumor immune response. These effects ultimately contribute to the progression of cancer and the facilitation of metastasis [15].

Beyond its dual role in cancer, TGF‐β signaling is also a central driver of fibrotic diseases across various organs, including the lungs, liver, kidneys, heart, skin, and pancreas. In pulmonary fibrosis, TGF‐β promotes fibroblast activation, EMT, and ECM deposition, resulting in irreversible architectural damage; these effects can be attenuated by agents that inhibit mitochondrion‐mediated oxidative stress [16, 17, 18]. In the liver, it induces hepatic stellate cell activation and collagen synthesis, central to hepatic fibrosis [19]. In renal tissues, TGF‐β triggers mesangial expansion and tubular atrophy, promoting chronic kidney disease [20]. Additionally, in cardiac fibrosis, TGF‐β contributes to myocardial remodeling and stiffness by enhancing ECM accumulation [21]. In the pancreas, its activation of pancreatic stellate cells underlies fibrotic remodeling associated with chronic pancreatitis and pancreatic cancer [22]. These profibrotic effects are mediated through both SMAD‐dependent and SMAD‐independent signaling pathways, underscoring the importance of TGF‐β as a shared molecular driver across both fibrotic and neoplastic diseases [23].

**FIGURE 1:**
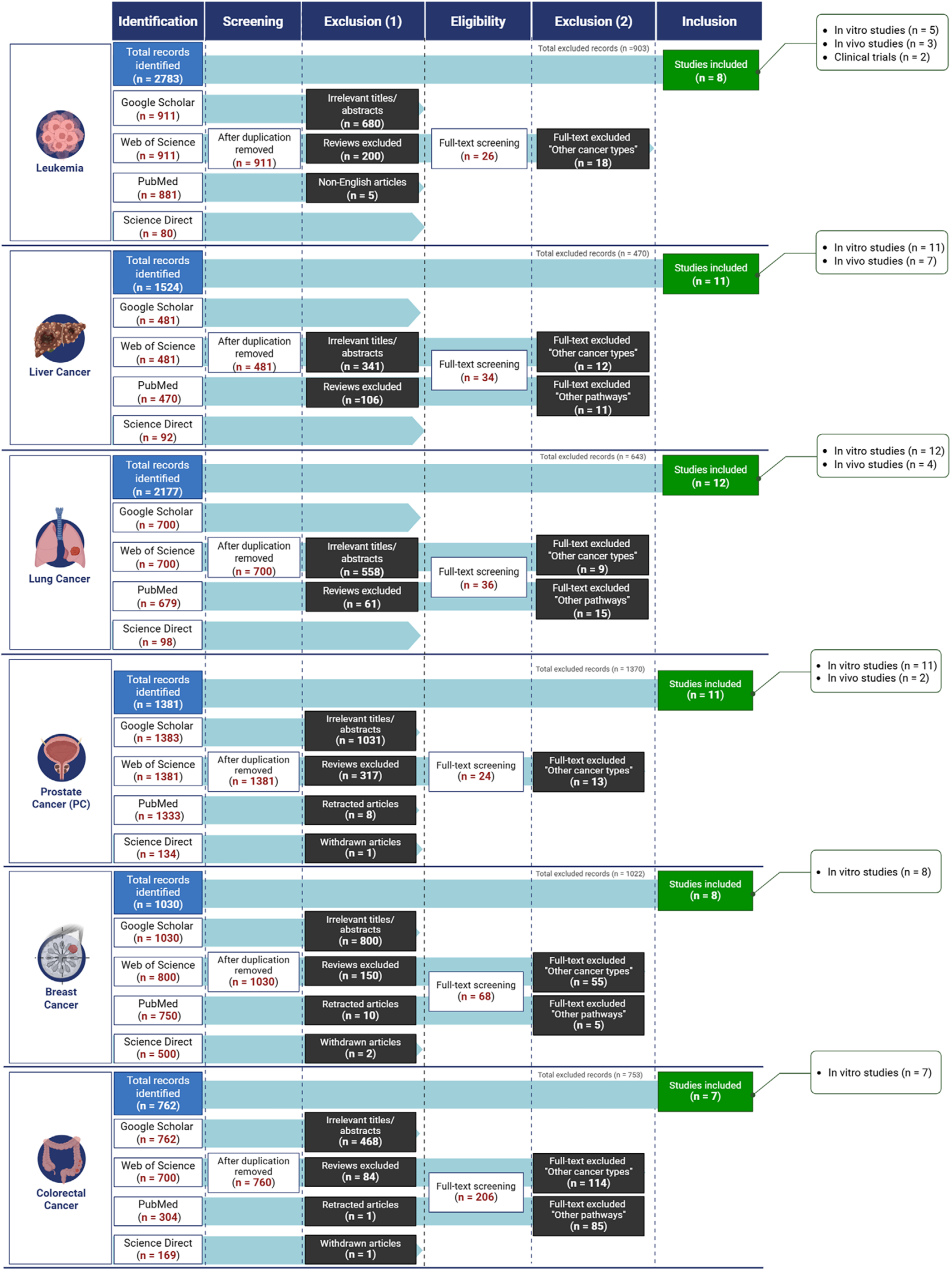
Workflow summary of literature selection across six cancer types based on database searches and predefined inclusion criteria. This figure presents the screening and selection process for studies investigating the role of TGF‐β signaling in prostate, breast, colorectal, leukemia, liver, and lung cancers. These cancer types were included based on their clinical relevance and the availability of mechanistic data related to TGF‐β modulation. The workflow outlines the number of records identified from major scientific databases, followed by exclusion steps based on relevance, cancer type, and signaling pathway specificity. The final number and type of included studies (in vitro, in vivo, and clinical) are shown for each cancer type.

The dualistic roles of TGF‐β in tumor suppression and promotion necessitate an in‐depth examination of its therapeutic modulation, aiming to elucidate its complex mechanisms and translational potential in cancer therapy. Upon activation, by forming complexes with Smad4, Smad proteins are able to migrate to the nucleus. They assemble corepressors, coactivators, transcription factors, DNA‐binding transcription factors, and chromatin remodeling factors within the nucleus. The constituents’ variation in expression may be accountable for the response to TGF‐β, which is influenced by the specific cell type and surrounding environment [24]. The SMAD2/3/4 complex, which is located in the nucleus, induces tumor‐promoting actions via stimulating cell proliferation. The mentioned factors are platelet‐derived growth factor subunit B, immunological suppression forkhead box P3 (Foxp3), the activation of EMT involves the activation of specific genes such as SNAIL/SLUG, ZEB1/ZEB2, and HMGA2. Conversely, the suppression of EMT involves the downregulation of genes such as E‐cadherin and cytokeratin. Additionally, the process of metastasis is facilitated by the expression of genes such as HDM2 and MMP‐9. These factors have the ability to limit tumor growth by inhibiting cell proliferation (p15, p21, p57, 4E‐BP1), promoting cell death (Bim, DAPK, GADD45β), and inducing autophagy (ATG5, ATG7, beclin‐1). Moreover, they inhibit angiogenesis (thrombospondin) and inhibit inflammation (Foxp3) [25]. While chemotherapy remains a cornerstone of cancer treatment, it can cause significant side effects in healthy cells, including nausea, vomiting, mucous membrane inflammation, hair loss, nerve damage, and bone marrow suppression [26, 27, 28]. Additionally, chemotherapy often leads to multidrug resistance, a major factor in over 90% of cancer‐related deaths among treated patients [29, 30]. The complex composition of cancer cells further limits the effectiveness of traditional treatments like chemotherapy and radiation, which can damage both cancerous and healthy cells, leading to severe blood toxicities and tissue damage [31]. Although many chemotherapeutics target specific pathways or molecules, cancer's adaptive nature often leads to resistance, challenging long‐term efficacy [31]. The plant kingdom, with over 350,000 documented vascular plant species and ongoing discoveries, serves as a rich source of bioactive compounds for drug development [32, 33]. This largely unexplored field offers vast potential for therapeutic discovery, with plants providing pharmacologically active components used in teas, extracts, and as foundations for semi‐synthetic pharmaceuticals [34]. Phytochemicals, including terpenes, alkaloids, essential oils, and flavonoids, possess notable medicinal properties, especially against cancer [35, 36]. Nearly 50% of current antitumor drugs are derived from natural products, valued for their roles in chemotherapy and chemoprevention by influencing cell cycle regulation, disrupting oncogenic pathways, and inhibiting tumor growth [37, 38]. Natural products offer chemo‐preventive benefits by reducing reactive oxygen species (ROS), enhancing DNA repair, and enabling immune surveillance to eliminate abnormal cells [39]. These compounds target cancer through diverse mechanisms such as promoting apoptosis, inhibiting cell proliferation, angiogenesis, and metastasis. Additionally, many natural products enhance chemotherapy's effectiveness in otherwise resistant cancers [40, 41]. Due to rising cancer rates and high treatment costs, there is an urgent need for affordable, safe, and accessible alternatives, making natural products an appealing substitute for synthetic drugs due to their minimal toxicity, diverse chemistry, and accessibility [42]. Natural products, particularly those affecting TGF‐β signaling, hold promise in overcoming limitations associated with conventional cancer treatments and this comprehensive review explores the therapeutic potential of natural compounds in modulating the TGF‐β pathway and their anticancer effects across various cancer types. A comprehensive literature search was performed to identify peer‐reviewed publications on the anticancer properties of natural compounds that modulate the TGF‐β signaling pathway. The following databases were utilized: Web of Science, ScienceDirect, Google Scholar, and PubMed/MedLine. The selection process involved specific inclusion and exclusion criteria to ensure the relevance and quality of the studies (Figure 1).

## 2 The TGF‐β Pathway in Cancer: Dual Roles and Therapeutic Targets

The therapeutic potential of natural compounds in cancer treatment lies in their ability to modulate various signaling pathways involved in cancer progression. This section provides an overview of recent findings on the interaction of natural compounds with the TGF‐β signaling pathway, a critical mediator of cell proliferation, apoptosis, and metastasis.

### 2.1 TGF‐β Pathway: From Tumor Suppression to Oncogenesis

The TGF‐β pathway is recognized for its complex roles in biological regulation, where it impacts cellular development and promotes cytostatic and apoptotic processes. Its influence varies significantly across cancer stages, where early tumor‐suppressive effects give way to later oncogenic actions in progressive cancers [43, 44]. This signaling system, which was first discovered to be a strong tumor suppressor, regulates a number of cellular functions, including migration, differentiation, apoptosis, and proliferation, and plays also a complex and varied role in cancer [45, 46].

#### 2.1.1 Tumor‐Suppressive Functions of TGF‐β in Early‐Stage Cancer

In normal cells and early‐stage cancers, TGF‐β limits cell proliferation, induces cell cycle arrest, and promotes apoptosis [47, 48]. Pointing to its tumor‐suppressive mechanisms, TGF‐β can reduce cell proliferation and induce apoptosis through various pathways [49].

- *Cell proliferation reduction*: Achieved through two transcriptional events: inducing CDK inhibitors and inhibiting C‐MYC expression. TGF‐β slows cell growth in neural, epithelial, and hematologic cells by targeting CDKs and their associated inhibitors (CDK‐IS), which regulate the progression of cell cycles beyond G1 during proliferation [50]. TGF‐β promotes the production of P15INK4B, P21CIP1, and P27KIP1, which inhibit the cyclin–CDK complex, leading to cell cycle arrest in the G1 phase [51]. When such CDK clusters are inactive, phosphorylation of retinoblastoma protein (pRb) is inhibited. Phosphorylation of pRb is the key factor in cell cycle progression, preventing G1 cells from entering the S phase. At the same time, TGF‐β reduces the expression of the oncogene C‐MYC, suppressing cell growth. C‐MYC allows cells to multiply forever and stimulates cell division [50]. In epithelial cells, the SMAD complexes induced by TGF‐β cooperatively control the decrease in C‐MYC expression along with transcription factors P107, E2F4/E2F5, and CCAAT/enhancer‐binding protein (C/EBP). This SMAD‐dependent cascade of TGF‐β is involved in multiple antiproliferative reactions, as TGF‐β inhibits P70 S6 kinase via protein phosphatase 2A, causing G1 phase cell cycle arrest [52].
- *Induction of apoptosis*: Regarding the hindering of apoptosis escape of the tumor, there are two primary pathways: SMAD‐dependent and independent. The SMAD‐dependent route includes proteins that promote apoptosis such DAPK, SHIP, and TIEG1. DAPK can enhance cytochrome C leakage and TGF‐β‐dependent cellular death by linking SMADs to mitochondrial proapoptotic processes [53]. SHIP disrupts the PI3K–Akt route, causing death of cells before survival [54]. TIEG1 may trigger oxidative stress and generate ROS [55]. Thus, it could be concluded that TGF‐β‐mediated SMAD signaling regulates the expression of proapoptotic proteins, which suppress tumor growth through apoptosis. With that TGF‐β SMAD‐independent route, TGF‐β‐mediated apoptosis activates caspase. TGF‐β suppresses antiapoptotic genes including BCL‐2 related proteins, BCL‐XL, and XLAP, while promoting proapoptotic genes like caspase‐8, caspase‐3, and Bcl‐2‐interacting killer [53]

#### 2.1.2 Oncogenic Role of TGF‐β in Advanced Cancer

In the context of advanced cancer, it assumes an oncogenic role, facilitating tumor initiation and metastatic progression [56]. Regarding TGF‐β role in cancer, the dysregulation within the TGF‐β signaling cascade or specific attenuation of its tumor‐suppressive functions enables neoplastic cells to evade the growth‐inhibitory actions of TGF‐β.

In pancreatic ductal adenocarcinoma (PDAC), one of the most aggressive and lethal cancers, the oncogenic behavior of TGF‐β is often intensified by frequent loss of SMAD4—a key component of its canonical signaling machinery [57]. When SMAD4 is inactivated, the classical SMAD2/3/4‐mediated pathway is impaired, causing TGF‐β signaling to shift toward noncanonical routes such as MAPK, PI3K/AKT, JNK, and Rho–GTPase pathways; these alternative pathways can drive hallmark malignant features, including EMT, tumor cell invasion, immunosuppression, and angiogenesis. In this altered signaling landscape, the tumor‐suppressive effects of TGF‐β are diminished, while its pro‐oncogenic actions prevail; accelerating tumor progression and limiting the effectiveness of current treatments [57]. Recognizing the dominant role of noncanonical signaling in SMAD4‐deficient PDAC opens new therapeutic opportunities, particularly through strategies that target both upstream TGF‐β activity and its downstream noncanonical mediators [58]. By exploiting TGF‐β‐initiated pathways, cancer cells harness its oncogenic potential to drive processes such as tumor growth, EMT, angiogenesis, and immune evasion [59, 60].

Mechanisms of oncogenic action:

- *EMT*: TGF‐β promotes EMT throughout normal development and growth. TGF‐β induces EMT, which promotes tumor cell migration and invasion. TGF‐β promotes EMT in cancer by regulating E‐cadherin, N‐cadherin, Snail, and vimentin transcriptional factors [60]. For instance, TGF‐β activates the transcription factor Snail and recruits the Sin3A/HDAC1/HDAC2 complex. This leads to deacetylation of the E‐cadherin promoter, inhibiting translation. Thus, TGF‐β promotes EMT [61]. TGF‐β1 temporarily activates the mesenchymal marker vimentin, according to multiple studies [62, 63]. Investigations suggest that TGF‐β1 binds to serine/threonine kinase receptors, phosphorylating Smad2 and Smad3, and forming a heteromeric complex with Smad4. The translocation of this complex to the nucleus influences the transcription of specific genes, such as vimentin [63, 64]. TGF‐β promotes the formation of mutant p53, p63 protein complex, and SMADs in p53‐mutated cancer cells. The ternary complex disrupts P63's tumor‐suppressive activity, allowing mutant p53 and TGF‐β to trigger EMT [65].
- *Angiogenesis*: TGF‐β promotes the angiogenesis by inducing proangiogenic growth factors including vascular endothelial growth factor (VEGF) and CTGF in fibroblast and epithelial cells. These substances induce endothelial cells to create capillaries and play an important role in producing and sustaining tumor angiogenesis, hence speeding cancer growth [66]. In addition, TGF‐β/SMAD4 interaction increases miR‐29a production, which targets PTEN and activates the AKT cascade. This promotes angiogenesis [67]. TGF‐β receptors have an important role in angiogenesis, in addition to their action as ligands. TGF‐β activates MMP9 and ALK5, a type I receptor, to promote the development of new blood vessels. TGF‐β–ALK5 signal transduction can increase the development of angiogenesis and invasion of cancers of the prostate and breast cells [68].
- *Immune evasion*: TGF‐β suppresses immunity to tumors and hinders host immunosurveillance by modulating immune cell levels and activity [60]. The signaling of TGF‐β inhibits the formation, differentiation, and activation of cytolytic natural killer (NK) cells [69]. A research investigation found that TGF‐β suppresses NK cells by altering their epigenetic makeup and upregulating the mTOR signaling pathway [70]. TGF‐β suppresses T cell growth and effector activity by suppressing the production of IL‐2, a cytokine that promotes CD4+ T cell development. TGF‐β signaling limits the cytotoxic program of CD8+ T cells by stimulating SMADs and transcription factor ATF1, repressing granzyme B and IFN‐γ, and affecting the lytic activity of CD8+ T cells [60]. Furthermore, TGF‐β utilizes IL‐2 to activate the gene transcription factor FOXP3 in naive CD4+ T cells, leading to their transformation to regulatory T (Treg) cells leading to the contribution of tumor growth and escaping from the immune response [71].

In conclusion, the TGF‐β pathway plays a dual role in cancer, acting as both a tumor suppressor in early stages and an oncogene in advanced stages. Understanding the intricate mechanisms of TGF‐β signaling is important for developing targeted therapies to exploit its tumor‐suppressive functions and inhibit its oncogenic activities.

### 2.2 Therapeutic Targeting of the TGF‐β Pathway in Cancer

The elevated presence of TGF‐β in both tumor cells and their microenvironment, coupled with compelling evidence of its prometastatic role and other different roles such as potentiation of growth, escaping from immune surveillance, and apoptosis, positions it as a promising target for therapy [72]. Poor initial outcomes and the rise of medication resistance persist as substantial challenges. Cancer researchers try to create more effective treatments with fewer adverse effects than traditional cancer therapy. Clinical trials have been conducted on several TGF‐β pathway inhibitors, monoclonal antibodies (mAbs), ligand traps, and antisense oligonucleotides (ASOs) [60].

### 2.3 Current Therapeutic Strategies

- *TGF‐β receptor kinase inhibitors*: Numerous TGF‐β receptor kinase inhibitors have been developed to engage with the ATP‐binding region of TGF‐β receptor kinase, thus inhibiting its activity and hindering the ensuing signaling cascade. Such as vactosertib, galunisertib, LY3200882, A83‐01, LY573636, SB‐431542, and SB‐505124, all work as TGF‐β kinase inhibitor hindering the tumor growth, invasion, and metastasis [73, 74, 75, 76, 77, 78].
- *mAbs*: Another class that are antibodies targeting TGF‐β obstruct either ligand activation or receptor binding. Both processes are necessary for TGF‐β to trigger protumorigenic and immunological suppressing effects. Such as SRK181‐mIgG1, fresolimumab, LY3022859, and 264RAD, all have been shown to reduce tumor growth and metastasis [79, 80, 81, 82].
- *Anti‐TGF‐β antibodies and ligand traps*: These are mostly in phase one clinical studies, such as Bintrafusp alfa [80] or in preclinical studies such as TGF‐βRII [83] and the only one of them that is in phase 3 clinical studies is Luspatercept [84].
- *Antisense oligonucleotides*: Concerning ASOs, they provide a unique strategy to specifically target and inactivate genes important in cancer growth, particularly those that are difficult to block using small compounds or mAbs [85]. TGF‐β2 expression is targeted by AP12009 and is being researched to treat malignant glioma, pancreatic cancer, and malignant melanoma using immunotherapy. AP12009 has undergone or is currently use in phase III trials against astrocytoma [86, 87], while AP11014 and AP15012 are antisense compounds are employed in preclinical trials for treating non‐small cell lung cancer (NSCLC), prostate carcinoma, and CRC respectively [88, 89]. Combining chemotherapeutic drugs with TGF‐β kinase inhibitors shows promise in preclinical and clinical studies, highlighting TGF‐β as a valuable cancer target and underscoring the importance of advancing understanding of its signaling pathways and refining patient selection to improve therapeutic outcomes.

## 3 Preclinical Pharmacological Studies

Preclinical pharmacological studies play a vital role in assessing the efficacy and mechanisms of natural compounds in modulating the TGF‐β pathway. This section provides an overview of both in vitro and in vivo studies, focusing on how specific compounds interact with TGF‐β signaling to inhibit cancer progression in different model systems.

### 3.1 In Vitro Studies Using Different Cancer Cells Line

#### 3.1.1 Leukemia

Homoharringtonine (HHT), initially derived from the plant *Cephalotaxus hainanensis*, was examined for its antileukemic properties on human acute myeloid leukemia (AML) cell lines, including KG‐1 and U937 [90]. The objective of this work was to validate that HHT has the ability to stimulate smad3, which subsequently triggers the activation of the TGF‐β pathway and suppresses cell proliferation in AML cell lines. In order to accomplish this, U937 cells were exposed to HHT at various concentrations of 0, 2, 4, and 8 ng/mL for a duration of 24 h. Similarly, KG‐1 cells were exposed to HHT at different concentrations of 0, 100, and 200 ng/mL for the same duration of 24 h. Next, the phosphorylation level of smad3 at Ser423/425 was quantified. The findings demonstrated a considerable rise in the level of Ser423/425 phosphorylated‐smad3 (p‐smad3) in U937 cells as the concentration of HHT rose. However, KG‐1 cells exhibited only a slight increase. These findings indicate that HHT has the ability to trigger smad3 phosphorylation.

The effects of HHT on the cycle state and apoptosis of the cell were evaluated in U937 cells and KG‐1 cells. Various concentrations of HHT (0, 2, 4, and 8 ng/mL) were used to treat U937 cells for 24 h, while KG‐1 cells were treated with varying concentrations of HHT (0, 30, 60, and 120 ng/mL) for the same duration. The objective was to determine the specific mechanism of cell death induced by HHT. The results demonstrated that HHT could lead to a decrease in the protein levels of c‐myc, cyclin‐dependent kinase 4 (CDK4), and CDK6, while simultaneously increasing the levels of p15. Nevertheless, there were no substantial alterations observed in p21, p27, and p57. By activating the TGF‐β pathway, suppressing downstream c‐myc, CDK4, and CDK6, and raising p15 expression, the data suggest that HHT can halt the U937 cell cycle during the G1 phase. The outcomes for KG‐1 cells exhibited significant dissimilarities. Following a 24‐h incubation period with HHT at various concentrations (0, 30, 60, and 120 ng/mL), it was shown that there was no notable alteration in the G1 phase of the cells. However, there was a substantial elevation in cell apoptosis. These findings indicate that HHT has the ability to suppress the growth of KG‐1 cells by triggering apoptosis instead of halting the cell cycle. The results indicated that HHT can impede the growth of both KG‐1 and U937 cells, albeit through different ways. KG‐1 cells are hindered by inducing apoptosis, whilst U937 cells are hindered by cell cycle arrest. To summarize, HHT induces Ser423/425 phosphorylation of Smad 3 in the AML cell lines U937 and KG‐1. Subsequent to this phosphorylation, the TGF‐β pathway is triggered, resulting in death in KG‐1 cells and cell cycle arrest in U937 cells specifically at the G1 phase [91].

Ginsenosides are triterpene saponins that are regarded as the primary bioactive components of herbal medicines obtained from the roots and rhizomes of various Panax species (Araliaceae). The ginsenosides can be categorized into two groups: 20(S)‐protopanaxadiol compounds (including ginsenosides Rb1, Rb2, Rb3, Rc, Rd, Rh2, Rg3, and others) and 20(S)‐protopanaxatriol compounds (including ginsenosides Re, Rg1, Rg2, Rh1, and others), with the exception of ginsenoside R0. Out of these, ginsenoside Rh2 has demonstrated the ability to hinder the proliferation of many human cell types by causing cell cycle arrest and death [92].

The effects of ginsenosides Rh2 on the cell cycle and the molecular mechanism involved were evaluated using HL‐60 promyelocytic leukemia cells. Rh2 was introduced to the HL‐60 cells at different concentrations ranging from 10 to 40 µM. TGF‐β1 has been shown to induce growth arrest and differentiation by increasing the expression of the cyclin‐dependent kinase inhibitors (CDKIs) p21^CIP1/WAF1^ or p27^KIP1^ in various cell lines. As a result, the administration of Rh2 was found to increase the production and gene expression of TGF‐β1, activate the Smad/FoxO3a signaling pathway, decrease the expression of CDK4,6, and inhibit the phosphorylation of pRb protein and the nuclear translocation of E2F1, which is essential for the transition of the cell cycle from G1 phase to S phase. The study revealed that Rh2 mediated G1 phase cell cycle arrest is associated with the enhanced recruitment of p21^CIP1/WAF1^ and p27^KIP1^ to CDK2, CDK4, and CDK6. Subsequently, there is a reduction in the phosphorylation of the Rb protein, leading to the inhibition of E2F1 translocation in HL‐60 cells. Furthermore, it was found that TGF‐β1 signaling is essential for the cell cycle arrest and differentiation produced by Rh2 in HL‐60 cells. Consequently, they deduce that ginsenoside Rh2 has the potential to be an effective treatment for leukemia [93]

Halofuginone (HF) is a small molecule quinazolinone alkaloid that has been shown to have strong antitumor effects because of its ability to inhibit cell growth and promote cell death [94]. Furthermore, it has been demonstrated in multiple solid tumor models to display antiangiogenic, antiproliferative, and proapoptotic properties. The researchers conducted an investigation to examine the effects of HF on NB4 cells in vitro. They focused on the relationship between enhanced angiogenesis, VEGF, and the progression of AML. The cells were treated with concentrations 50, 100, and 200 ng/mL of HF for 6, 12, and 24 h. The data revealed an increased expression of tissue inhibitor of metalloproteinases 2 (TIMP2) and chemokine ligand 10 (CXCL10), which are antiangiogenic factors. Additionally, there was a decrease in the expression of proangiogenic factors, including hepatic growth factor (HGF), hypoxia‐inducible factors‐α (HIF‐1α), angiopoietin (ANGPT‐1 and ANGPT‐2), as well as VEGF gene expression. The findings indicate that HF, a novel anticancer drug, inhibits smad2 signaling and suppresses TGF‐β signaling, effectively reducing angiogenesis and leukemia growth, both of which are crucial factors in tumor progression [95]. The cells NB4 and its derivative NB4‐R2 are the focus of another in vitro study that employs HF. In this experiment, the NB4 cell line was exposed to HF at concentrations of 50, 100, or 200 ng/mL. The results demonstrated that HF therapy enhanced TGF‐β signaling, as evidenced by the upregulation of TGF‐β1 expression, phosphorylation of Smad 3, and increased expression of TGF‐β receptor II (TGF‐βRII). The results showed an increase in the expression of p15 and p21, which are CDKIs, and a decrease in the expression of c‐myc. This could have contributed to the suppression of TGF‐β‐induced cell growth and programmed cell death (apoptosis), in line with the activation of the TGF‐β pathway caused by HF [96].

Epigallocatechin gallate (EGCG), a type of polyphenol included in green tea (GT), triggers apoptosis and autophagy in human mesothelioma cells [97]. The cytotoxic and apoptotic effects of EGCG were evaluated on the K562 chronic myeloid leukemia cell line. The cells were subjected to continuous treatment with 100 µM EGCG for 24, 48, and 72 h. EGCG exerted a suppressive effect on growth and triggered apoptosis in K562 cells. Moreover, the apoptosis it triggers is associated with the excessive production of the TGF‐β2 gene and the suppression of the CDC25A and cyclin D1 genes [98].

Bufalin has demonstrated anticancer properties by provoked cell cycle arrest, cellular demise, and cellular differentiation in a diverse range of human cancer cell types, such as hepatocellular carcinoma, prostate, gastric, and leukemia cells. Bufalin efficiently inhibits TGF‐β‐induced EMT and migration in A549 cells. Transcription factors, including Twist, Twist2, Snail, Slug, and ZEB2, inhibit the expression of E‐cadherin. Bufalin effectively suppresses the increased expression of Twist2 and ZEB2. The phosphorylation of Smad2 and Smad3, produced by TGF‐β, is markedly inhibited after a 24‐h treatment with 50 nM bufalin. TβRI directly phosphorylates Smad2 and Smad3, while TGF‐β‐bound TβRII phosphorylates and activates TβRI. The expression of TβRI and TβRII is considerably reduced after treating A549 cells with bufalin [99].

**FIGURE 2:**
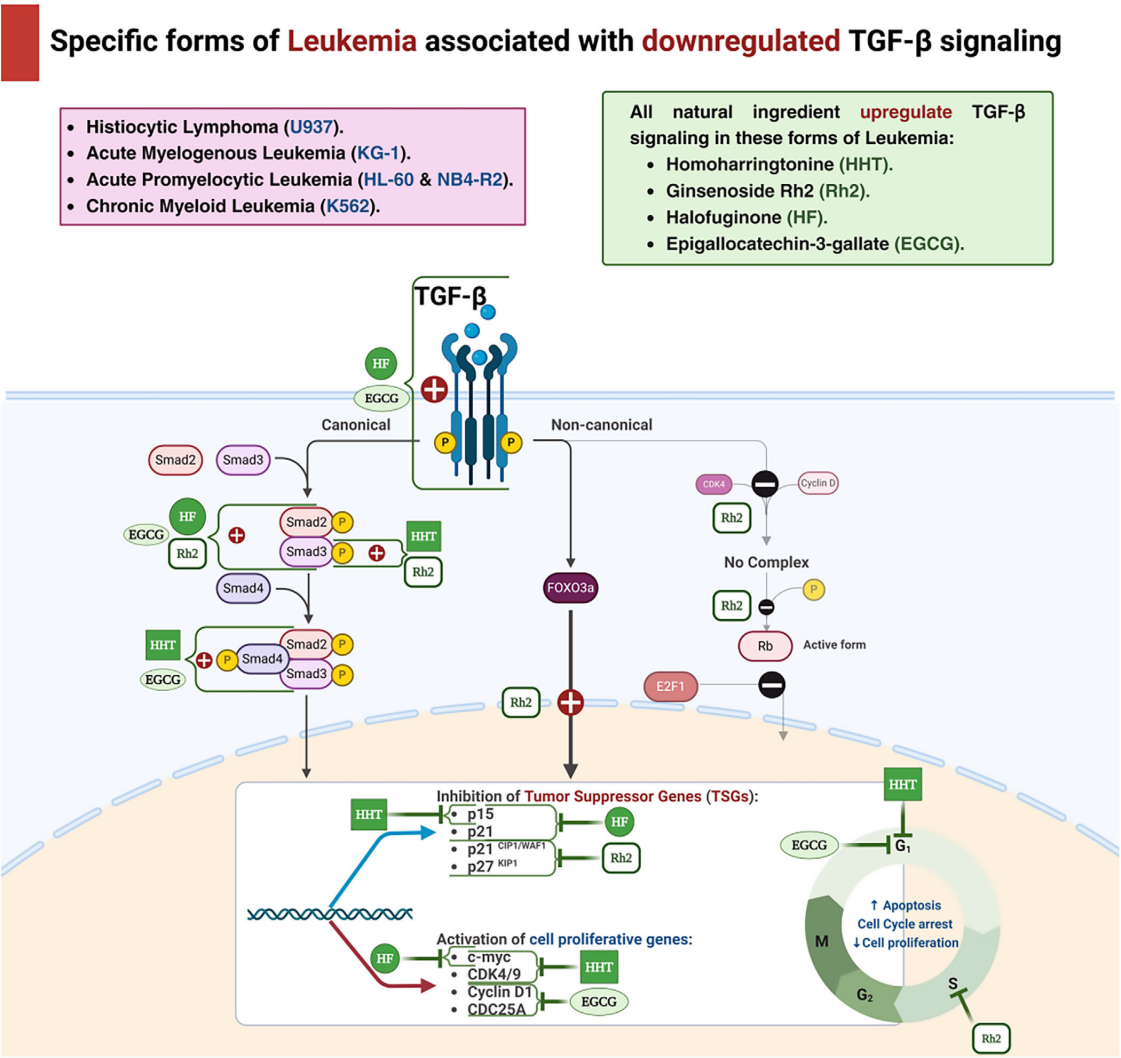
Natural products downregulating TGF‐β pathway in treatment of leukemia. The schematic illustrates the modulation of TGF‐β signaling by various natural compounds in different forms of leukemia, specifically histiocytic lymphoma (U937), acute myelogenous leukemia (KG‐1), acute promyelocytic leukemia (HL‐60 and NB4‐R2), and chronic myeloid leukemia (K562). Natural compounds such as homoharringtonine (HHT), ginsenoside Rh2 (Rh2), halofuginone (HF), and epigallocatechin‐3‐gallate (EGCG) upregulate TGF‐β signaling pathways, leading to varied effects on cell proliferation, apoptosis, and cell cycle arrest. The canonical pathway involves Smad2/3 phosphorylation, which complexes with Smad4 and translocates to the nucleus to regulate gene expression. The noncanonical pathway involves FOXO3a activation. Key mechanisms include the inhibition of tumor suppressor genes (TSGs) like p15, p21, and p27, and the activation of cell proliferative genes such as c‐myc, CDK4/9, cyclin D1, and CDC25A. The diagram also highlights the resulting cellular outcomes such as increased apoptosis, cell cycle arrest, and reduced cell proliferation. CDC25A, cell division cycle 25A; CDK, cyclin‐dependent kinase; EGCG, epigallocatechin‐3‐gallate; FOXO3a, Forkhead box O3; HHT, homoharringtonine; HF, halofuginone; Rh2, ginsenoside Rh2; Smad, mothers against decapentaplegic homolog; TGF‐β, transforming growth factor‐beta; TSG, tumor suppressor genes.

**FIGURE 3:**
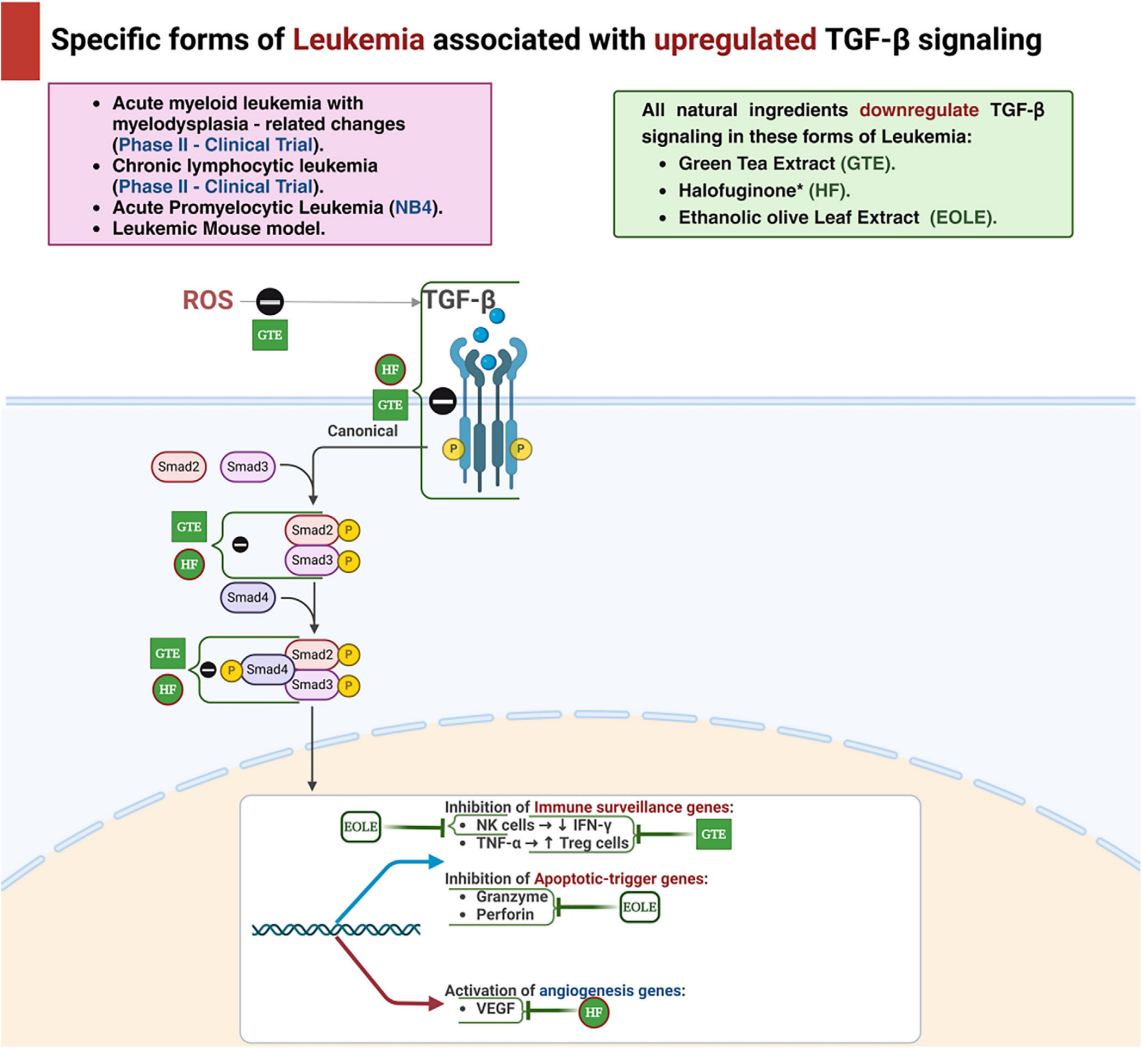
Natural products upregulating TGF‐β pathway in treatment of leukemia. This schematic represents the modulation of TGF‐β signaling by natural compounds in specific forms of leukemia, including acute myeloid leukemia with myelodysplasia‐related changes, chronic lymphocytic leukemia, acute promyelocytic leukemia (NB4), and leukemic mouse model. Natural ingredients such as green tea extract (GTE), halofuginone (HF), and ethanolic olive leaf extract (EOLE) are shown to downregulate TGF‐β signaling. The canonical TGF‐β signaling pathway involves the phosphorylation of Smad2/3, which then forms a complex with Smad4 and translocates to the nucleus to influence gene expression. Key impacts of these natural compounds include inhibition of immune surveillance genes (e.g., reduced NK cells and IFN‐γ, increased TNF‐α and Treg cells), inhibition of apoptotic‐trigger genes (granzyme, perforin), and activation of angiogenesis genes (VEGF). This diagram highlights the intricate roles of GTE, HF, and EOLE in modulating TGF‐β signaling and their potential therapeutic effects in leukemia. EOLE, ethanolic olive leaf extract; GTE, green tea extract; HF, halofuginone; IFN‐γ, interferon‐gamma; NK, natural killer; ROS, reactive oxygen species; Smad, mothers against decapentaplegic homolog; TGF‐β, transforming growth factor‐beta; TNF‐α, tumor necrosis factor‐alpha; Treg cells, regulatory T cells; VEGF, vascular endothelial growth factor.

Key mechanisms and effects of natural compounds on the TGF‐β pathway in leukemia cell lines are summarized in Table 1 and Figures 2 and 3.

**TABLE 1:** In vitro effects of natural compounds on TGF‐β pathway in leukemia cell lines.

Compound (chemical class)	Cancer cell lines	Tested concentrations	Mechanism/signaling pathways	Results (effect on cancer cells)	References
Homoharringtonine (alkaloid)	U937, KG‐1	0, 2, 4, 8 ng/mL (U937); 0, 100, 200 ng/mL (KG‐1)	Activates TGF‐β pathway ↑p‐Smad3 (Ser423/425) ↓c‐myc, ↓CDK4 ↓CDK6, ↑p15	U937: Cell cycle arrest at G1 phase; KG‐1: Apoptosis	[91]
Halofuginone (alkaloid)	HL‐60	10–40 µM	↑TGF‐β1 expression ↑Smad/FoxO3a ↓CDK4, ↓CDK6 ↓p‐Rb	G1 phase cell cycle arrest ↓E2F1 translocation	[92, 100]
Ginsenoside Rh2 (saponin)	NB4, NB4‐R2	50, 100, 200 ng/mL	↓Smad2 signaling, ↑TGF‐β1, ↑p‐Smad3, ↑TGF‐βRII, ↑TIMP2, ↑CXCL10 ↓VEGF, ↓HGF ↓HIF‐1α, ↓ANGPT	↓Angiogenesis ↓Cell growth ↓Migration ↑Apoptosis	[95, 96]
Epigallocatechin gallate (polyphenol)	K562	100 µM	↑TGF‐β2, ↓CDC25A, ↓cyclin D1	↓Growth ↑Apoptosis	[98]
Bufalin (steroidal compound)	A549	50 nM	↓TGF‐β‐induced EMT, ↓Twist2, ↓ZEB2, ↓p‐Smad2, ↓p‐Smad3 ↓TβRI, ↓TβRII	↓EMT ↓Migration	[99]

Abbreviations: ↑, increase; ↓, decrease; CDK, cyclin‐dependent kinase; CXCL, chemokine ligand; EMT, epithelial–mesenchymal transition; HGF, hepatic growth factor; HHT, homoharringtonine; HIF, hypoxia‐inducible factors; p‐Rb, phosphorylated retinoblastoma protein; Smad, mothers against decapentaplegic homolog; ANGPT, angiopoietin; TGF‐β, transforming growth factor‐beta; TIMP, tissue inhibitor of metalloproteinases.

#### 3.1.2 Lung Cancer

Oroxylin A (OXA), a flavonoid derived from *Scutellariae radix*, suppresses the formation of Treg cells in NSCLC. Oleanolic acid (OA) was extensively utilized for its anti‐inflammatory and anticancer properties. OA, at a concentration of 40 µM, hinders the release of TGF‐β1 by lung cancer cells by reducing the NF‐κB signaling pathway. This, in turn, reduces the production of Treg cells in the lung cancer environment. OA directly reduces the response of T cells to TGF‐β1, which then hinders the functioning of Treg cells. TGF‐β is an important cytokine that plays a vital role in the process of differentiating CD4+ nT cells into CD4+CD25+Foxp3+ Treg cells. Treg cells were generated through coculturing PBMC with H460 cells, resulting in the activation of p‐Smad3, p‐ERK, p‐JNK, and p‐p38. Furthermore, OA also suppressed the activation of these proteins in the coculture experiment. These data indicate that OA influences the development of Treg cells by affecting both the Smad and MAPK signaling pathways [101].

Resveratrol, also known as trans‐3, 4, 5‐trihydroxystilbene or RES, is a type of polyphenolic molecule that is classified as a stilbene Plants spontaneously synthesize it as a defense mechanism against pathogenic illness. Resveratrol demonstrates advantageous health benefits such as its ability to act as an antioxidant, reduce inflammation, protect the heart, and inhibit tumor growth; it also influences the development and advancement of tumors [102]. The results suggest that resveratrol inhibits the formation of new blood vessels and the spread of cancer cells to other parts of the body. The data we obtained indicate that a concentration of 20 µM resveratrol commences TGF‐1‐induced EMT by upregulating the expression of the mesenchymal phenotype markers fibronectin and vimentin while increasing the expression of the epithelial phenotypic marker E‐cadherin. Resveratrol further suppresses the expression of EMT‐inducing transcription factors Snail1 and Slug [103].

Salvianolic acid B (Sal B) has been identified as a cytotoxic polyphenol with potential therapeutic use in various types of cancer, such as hepatocellular carcinoma, breast cancer, head and neck squamous cell carcinoma, gastric cancer, and colorectal cancer. Additionally, Sal B was found to suppress the growth of A549 cells, a type of NSCLC. The dose at which it inhibits 50% of cell growth, known as the half maximum inhibitory concentration (IC50), was reported to be 279.6 µM. A549 cells were subjected to treatment with Sal B at concentrations of 25, 50, and 100 µM. Sal B counteracted these effects caused by TGF‐β1 and suppressed the EMT and A549 cells migration. Moreover, the administration of Sal B resulted in the inhibition of cell proliferation. This was evident by the suppression of cell cycle progression and the regulation of protein expression levels of important markers, such as cyclin B1 and p21, in TGF‐β1‐stimulated A549 cells. Following that, Sal B caused a rise in the levels of autophagy marker proteins, LC3β and Beclin1, and a decrease in the levels of p62 protein production. Sal B caused a rise in the rates of programmed cell death and the levels of protein expression of markers for programmed cell death, such as caspase‐3/cleaved‐caspase‐3 and Bax. However, the protein expression of Bcl‐2 dropped. The study showed that Sal B strongly suppressed the phosphorylation of Smad2/3 caused by TGF‐β1, leading to the suppression of PAI‐1 protein expression levels. Furthermore, Sal B suppressed the phosphorylation of ERK1/2, JNK1/2, and p38, which resulted in the inhibition of the ERK, JNK, and p38 MAPK signaling pathways in A549 cells triggered by TGF‐β1 [104].

Hypaconitine (HpA) is a naturally occurring compound obtained from the root of aconitum species, which is used in traditional Chinese medicine. Prior research has demonstrated that HpA exhibited toxicity toward HepG2, HCT8, and MCF7 tumor cells. Therefore, the impact of HpA on the viability of A549 cells was initially assessed in this investigation. HpA (16 µmol/L) suppressed the growth of A549 cells. To reduce the harmful impact on EMT, a concentration of 8 µmol/L of HpA was employed in subsequent investigations. The findings of our study showed that HpA partially counteracted the morphological changes generated by TGF‐β1. In addition, HpA partially reversed the switch from E‐cadherin to N‐cadherin that was induced by TGF‐β1, by inhibiting Snail. The findings suggested that HpA suppressed the TGF‐β1‐induced EMT in A549 cells. The induction of EMT by TGF‐β1 has been documented to have a significant role in the process of metastasis. In this study, we discovered that HpA significantly inhibited the adhesion, migration, and invasion produced by TGF‐β1 in A549 cells. These findings suggest that HpA may possess antimetastatic properties. HpA exhibited comparable inhibitory efficacy to pyrrolidine dithiocarbamate, a known NF‐κB inhibitor, in suppressing TGF‐β1‐induced morphological changes, alteration of EMT biomarkers, adhesion, migration, and invasion. Our findings also demonstrated that HpA reduced the phosphorylation of IκBα, which is triggered by TGF‐β1. This impact was also associated with a simultaneous decrease in the movement of the p65 component of NF‐κB into the nucleus. Therefore, the suppressive impact of HpA was somewhat facilitated by deactivation of NF‐κB. Snail, Twist, and zinc finger E‐box binding (ZEB) transcription factors collectively have a role in suppressing the epithelial characteristics and promoting the mesenchymal characteristics [105].

Kaempferol, a naturally occurring flavonoid found in food, is widely recognized for its ability to manage and prevent cancer. The aim of this attempt was to examine the inhibitory impact and underlying mechanisms of kaempferol on EMT and cell migration induced by TGF‐β1. Kaempferol (25 µM) effectively inhibited the promotion of cell migration generated by TGF‐β1‐mediated EMT in human A549 non‐small lung cancer cells. This was achieved by restoring the loss of E‐cadherin, increasing the activity of TGF‐β1‐mediated MMP2, and reducing the levels of N‐cadherin and vimentin. Remarkably, kaempferol counteracted the effects of TGF‐β1 on Snail induction and E‐cadherin repression by weakening Smad3 binding to the Snail promoter. It specifically inhibited the phosphorylation of Smad3 at Thr179 (pSmad3L), without affecting phosphorylation at Ser204, Ser208, or Ser213. Furthermore, the administration of TGF‐β1 to A549 cells led to a prompt activation of Akt by phosphorylation. However, the presence of kaempferol entirely nullified this effect [106]

Ursolic acid is a pentacyclic triterpenoid chemical obtained from many medicinal herbs and has been shown to have several anticancer effects, such as inhibiting the formation of tumors, promoting tumors, and the growth of new blood vessels, as well as causing programmed cell death in different types of cancer cells, including breast, gastric, cervical cancer, hepatocellular carcinoma, and human NSCLC. Our investigation found that a concentration of 20 nM of ursolic acid was able to effectively prevent TGF‐1‐induced cell migration. Additionally, it significantly reduced the activity of MMP‐2 and ‐9 enzymes and caused a notable inhibition in the expression of integrin V5. Furthermore, ursolic acid suppressed the cadherin transition caused by TGF1, resulting in a rise in E‐cadherin and a decrease in N‐cadherin [107].

Cucurbitacin B (CuB) is an organic chemical obtained from the Cucurbitaceae plant, which is a Chinese herb [108]. CuB has been identified as a promising candidate molecule for the treatment of NSCLC due to its ability to target the PI3K/Akt and MAPKs signaling pathways. The expression of p‐PI3K, p‐Akt, and p‐mTOR was reduced by CuB at concentrations of 5, 10, and 15 nM. In addition, CuB upregulated E‐cadherin and downregulated ZEB1, EGFR, N‐cadherin, and vimentin expression, while also inhibited the formation of ROS [109].

Berberine, an isoquinoline alkaloid present in barberry, tree turmeric, and other plants, is renowned for its strong antioxidant, anti‐inflammatory, and anticancer characteristics. In a recent study, it has been examined the effectiveness of a berberine LCN formulation based on monoolein (BM‐LCNs) in treating airway remodeling and EMT. We induced these conditions by activating BEAS‐2B human bronchial epithelial cells with TGF‐β. Administration of 0.5 µM BM‐LCNs effectively countered the effects of TGF‐β by decreasing the levels of endoglin, basic FGF, myeloperoxidase (MPO), thrombospondin‐1 (THBS1), and VEGF. Additionally, it partially restored the production of cystatin C and the secretion of NO [110].

Andrographolide (Andro) is a type of diterpene lactone that has demonstrated many pharmacological properties, including cancer [111]. Consequently, Andro is particularly relevant for developing novel cancer therapies [7]. Recent investigations have demonstrated that Andro effectively regulates the tumor angiogenic TGF‐β1/prolyl hydroxylase 2 (PHD2)/HIF‐1α/VEGF signaling pathway. Specifically, treatment with Andro (5.0 µM) resulted in significant suppression of HIF‐1α, TGF‐β1, Smad, and VEGF protein levels in NSCLC cell lines H1355, H1299, CL1‐0, and CL1‐5. Furthermore, Andro treatment enhanced the expression of PHD2 and hydroxyHIF‐1α, indicating its potential utility in targeting angiogenic processes within tumors [112].

Nobiletin (5, 6, 7, 8, 3′, 4′‐hexamethoxyflavone) is a prominent compound found in citrus fruits. It has been discovered to possess antimetastatic properties. Therapy with TGF‐β1 resulted in an upregulation of vimentin, N‐cadherin, Snail, Slug, ZEB1, Twist, and Smad, and a downregulation of E‐cadherin. However, the expression of E‐cadherin was restored by nobiletin therapy. Furthermore, nobiletin had a suppressive effect on the expression of MMP‐2 and MMP‐9. TGF‐β1 greatly stimulated the phosphorylation of Src, focal adhesion kinase (FAK), and paxillin in A549 cells. However, nobiletin significantly suppressed this stimulation [113].

Neferine is the primary bisbenzylisoquinoline alkaloid that is commonly ingested as a fundamental component in traditional Chinese medicine. Prior research has demonstrated that neferine possesses numerous pharmacological properties, such as antiarrhythmic, antihypertensive, anticancer, and cholinesterase inhibitory actions. Neferine reduces the survival, movement, and infiltration of lung cancer cells in laboratory conditions by promoting pyroptosis through the suppression of TGF‐β, as well as by suppressing EMT and controlling the expression of macrophage‐stimulating1 (MST1). Neferine administration significantly decreased the expression of procaspase‐1, while significantly increasing the expression of cleaved caspase‐1, IL‐1β, IL‐18, gasdermin D (GSDMD), and cleaved GSDMD. Furthermore, neferine therapy resulted in the enhancement of E‐cadherin expression, accompanied by the suppression of N‐cadherin and vimentin expression, hence influencing cellular EMT [114].

Fisetin (Fis), (3,3′,4′,7‐tetrahydroxyflavone), is present in a number of fruits and vegetables, such as cucumbers, apples, grapes, strawberries, and onions [115]. Several animal models and cell cultures have reported the valuable effects of Fis against oxidative stress, inflammation, and cancer [116]. According to an earlier in vitro study, treating HCC‐LM3 and HepG2 cells with Fis (1, 2.5, 5, 10, 20, 40, and 80 µM) significantly suppressed liver cancer cell invasion and migration. The protective effect of Fis against HCC was attributed to blocking the TGF‐β1 signaling pathway, which led to the suppression of EMT as seen by the downregulation induced in TGF‐β expression and a significant decrease in vimentin and N‐cadherin levels after Fis was added. Meanwhile, Liu et al. [117] confirmed that Fis hindered the EMT‐related signaling pathway also by suppressing the expression levels of p38, ERK1/2, and JNK in HCC‐LM3 cells. This was confirmed by the diminished expression levels consequently to Fis. The main effects of natural compounds on TGF‐β signaling in lung cancer cell lines are summarized in Table 2.

**TABLE 2:** In vitro effects of natural compounds on TGF‐β pathway in lung cancer cell lines.

Compound (chemical class)	Cancer cell lines	Tested concentrations	Mechanism/signaling pathways	Effect on cancer cells	References
Oroxylin A (flavonoid)	H460	40 µM	↓NF‐κB signaling, affects Smad and MAPK signaling pathways	↓Treg cells formation ↓p‐Smad3, ↓p‐ERK, ↓p‐JNK, ↑p‐p38	[101]
Resveratrol (polyphenol)	NSCLC	20 µM	↑Mesenchymal markers, ↑Epithelial marker expression	↓EMT ↓Snail1, ↓Slug	[103]
Salvianolic acid B (polyphenol)	A549	25, 50, 100 µM	↓Smad2/3 phosphorylation, ↓ ERK1/2, JNK1/2, and p38 MAPK signaling pathways	↓EMT ↓Cell proliferation and migration ↑Autophagy	[104]
Hypaconitine (alkaloid)	A549	16, 8 µmol/L	↓NF‐κB signaling, ↓Phosphorylation of IκBα and nuclear translocation of p65	↓EMT ↓Cell adhesion ↓Migration ↓Invasion	[105]
Kaempferol (flavonoid)	A549	25 µM	Blocks pSmad3L activation at Thr179, ↑E‐cadherin, ↓MMP2 activity	↓EMT ↓N‐cadherin ↓Vimentin ↓Cell migration	[106]
Ursolic acid (triterpenoid)	NSCLC	20 nM	↓MMP‐2 and ‐9 activity, ↓Integrin V5 expression	Prevents TGF‐β1‐induced cell migration ↑E‐cadherin ↓N‐cadherin	[107]
Cucurbitacin B (triterpenoid)	NSCLC	5, 10, 15 nM	Targets PI3K/Akt and MAPKs signaling pathways, ↓ROS	↑E‐cadherin, ↓ZEB1 ↓EGFR, ↓N‐cadherin ↓Vimentin	[109]
Berberine (alkaloid, BM‐LCNs)	BEAS‐2B	0.5 µM	↓Endoglin, basic FGF, ↓MPO, ↓THBS1,↓VEGF, ↑Cystatin C, ↓NO	Counteracts TGF‐β effects ↓Airway remodeling and EMT	[110]
Andrographolide (diterpenoid)	H1355 H1299 CL1‐0 CL1‐5	5.0 µM	↓TGF‐β1/PHD2/HIF‐1α/VEGF pathway	↓HIF‐1α, ↓TGF‐β1, ↓Smad, ↓VEGF, ↑PHD2, ↓hydroxyHIF‐1α	[112]
Nobiletin (flavonoid)	A549	Not specified	↓Src, ↓FAK, ↓paxillin phosphorylation	↓EMT, ↑E‐cadherin ↓MMP‐2, ↓MMP‐9	[113]
Neferine (alkaloid)	NSCLC	Not specified	↑Pyroptosis, ↓EMT, controls MST1 expression	↓Survival movement, and infiltration of lung cancer cells, ↑pyroptosis	[114]
Fisetin (flavonoid)	HCC‐LM3, HepG2	1, 2.5, 5, 10, 20, 40, 80 µM	↓TGF‐β1 signaling pathway, ↓p38, ERK1/2, and JNK	↓Invasion and migration, ↓Vimentin and N‐cadherin levels	[116]

Abbreviations: A549, human lung carcinoma cell line; BM‐LCNs, berberine‐loaded cubosomes; CuB, cucurbitacin B; EMT, epithelial–mesenchymal transition; ERK, extracellular signal‐regulated kinase; FAK, focal adhesion kinase; FGF, fibroblast growth factor; Fis, fisetin; H1355, H1299, CL1‐0, CL1‐5, various NSCLC cell lines; HCC‐LM3, HepG2 ‐ hepatocellular carcinoma cell lines; HpA, hypaconitine; IκBα, inhibitor of nuclear factor kappa‐B kinase subunit alpha; JNK, c‐Jun N‐terminal Kinase; MAPK, mitogen‐activated protein kinase; MPO, myeloperoxidase; MST1, macrophage‐stimulating 1; NF‐κB, nuclear factor kappa‐light‐chain‐enhancer of activated B cells; OXA, oroxylin A; PHD2, prolyl hydroxylase 2; pSmad3L, phosphorylated Smad3 linker; RES, resveratrol; ROS, reactive oxygen species; TGF‐β, transforming growth factor‐beta; THBS1, thrombospondin‐1; ZEB1, zinc finger E‐box binding homeobox 1.

#### 3.1.3 Liver Carcinoma

The scarlet resin of *Dracaena cochinchinensis* is known as Chinese dragon's blood (Lour.) S. C. Chen [118]. For many years, Chinese dragon's blood has been used to cure a variety of illnesses in China, such as skin disorders, gynecological illnesses, cardiovascular illnesses, otorhinolaryngological illnesses, and complications from diabetes mellitus [119]. An earlier study suggested that Chinese dragon's blood EtOAc extract (CDBEE) had antihepatoma activity [120]. Experimental evidence has shown that when HepG2 and SK‐HEP‐1 cells were exposed to different concentrations of CDBEE (0, 10, 20, 40, and 80 µg/mL) for 24, 48, and 72 h, there was a notable reduction in cell viability, migration, and invasion. The IC50 values of CDBEE in HepG2 and SK‐HEP‐1 cells were documented as 27.84 and 32.06 µg/mL, respectively. CDBEE exerted suppressing effect on Smad3, led to activation of the MAPK signaling pathway, resulted in inducing apoptosis, and inhibition of TGF‐β1‐induced EMT confirmed by the higher phosphorylation of p38, ERK, and PARP, while decrease in the expression of mesenchymal markers N‐cadherin and Slug in HepG2 and SK‐HEP‐1 cells.

Tanshinone IIA (TanIIA), a traditional Chinese medicine, is isolated from *Salvia miltiorrhiza*. TanIIA is known to possess antitumor effects, that was attributed to modulation of drug resistance, invasion, apoptosis, and tumor cell proliferation. Indeed, it was found that TanIIA (0, 5, 10, 20, 40, 80, and 160 µM) significantly inhibited the proliferation in Bel‐7404, SMMC‐7721, and Bel‐7402 cells, and that the IC50 of TanIIA was 40 µM. Further, Bel‐7404, SMMC‐7721, and Bel‐7402 cells exhibited a significant inhibition in migration and invasion while induction in apoptosis in response to TanIIA (40 µM). TanIIA was found to suppress EMT, verified by the elevation in E‐cadherin levels and decrease in levels of N‐cadherin, Ki67, cyclin D1, and Bcl2. TanIIA also blocked Smad2/3 phosphorylation expression by upregulating the expression of Smad7, confirmed by the increase in Smad7 expression and the downregulation of P‐Smad2 and P‐Smad3 [121].

OA, also known as 5,7‐dihydroxy‐6‐methoxyflavone, is a naturally occurring substances derived from *Scutellaria radix*. OA is known to possesses multiple anticarcinoma effects that relay mainly on induction of apoptosis and cell differentiation [122, 123]. OA was utilized for breast cancer cells and non‐small‐cell lung cancer cells owing to its inhibitory role in suppressing cell invasion and metastasis [124, 125]. OA was shown to have suppressive effects on HCC [126].  SMMC‐7721 cells incubated with OA (50 µM) for 48 h significantly expressed a suppression in migration, invasion, and induction in apoptosis. The role of OA in suppressing TGF‐β1‐induced EMT in HCC cells was confirmed by the decreased levels of N‐cadherin, vimentin, MMP2, MMP9, Twist1, and PAI‐1 expression, while the E‐cadherin level was raised upon treating SMMC‐7721 cells with OA. OA was also found to participate in inhibiting TGF‐β1/Smad signaling by inducing HDAC1 degradation. This in turn would hamper HDAC1‐mediated deacetylation of C/EBPβ, leading to an increase in NAG‐1 expression, and subsequently amelioration in HCC. Notably, cotreated OA (50 µM) significantly induced NAG‐1 expression in the cytoplasm and nucleus of HCC cells, increasing the acetylation of the C/EBPβ transcription factor, while decreasing HDAC1 protein.

Sulforaphane (SFN), an isothiocyanate compound, is obtained from cruciferous vegetables, specifically broccoli. It has proven to be an effective antitumor drug in a variety of malignancies [127]. Wu et al. [128] proposed the promising role of SFN in treating HCC. SFN was found to inhibit TGF‐β1‐induced EMT. It has been demonstrated that upon treating cell line HepG2 with SFN (vehicle, 10, 20, 40, 60, and 80 µM) for 48 h, a significant suppression in proliferation was reported in HepG2 cells in a dose‐dependent manner, and the IC50 of SFN in HepG2 cells was reported to be 40.05 µM. Additionally, an arrest in the cell cycle at the G0/G1 phase and an increase in apoptosis were observed following treatment HepG2 cells with SFN (40 µM). Furthermore, SFN (40 µM) increased rate of apoptosis and induced an arrest in the cell cycle at the G0/G1 phase. Furthermore, SFN (40 µM) attenuated the characteristic morphological changes of EMT caused by (10 ng/mL) TGF‐β, inhibiting the formation of fibroblast‐like mesenchymal cells, reducing the expression of vimentin while significantly prompting the expression of E‐cadherin in HepG2In addition, the application of SFN (40 µM) resulted in a considerable reduction in the number of migratory HepG2 cells, suggesting that SFN has a favorable inhibitory impact on the migration and invasion of cells.

Sal B is a prominent phenolic compound obtained from *Salvia miltiorrhiza Bunge* (Labiatae). Sal has been shown to be a successful treatment for hepatic conditions [129]. Smad3 tends to be an essential downstream target that mediates the malignant impacts of TGF‐β1. The kinase TGF‐β1 receptor type I (TpRI)/pSmad3C pathway has been implicated in suppressing tumor hyperplasia by preventing epithelial cell proliferation. Conversely, the JNK/pSmad3L pathway has been shown to facilitate signaling that fosters tumor cell invasion and carcinogenesis. A previous study showed that Sal B had effects against hepatocarcinogenesis provoked by diethyl nitrosamine (DEN). The study attributed the ameliorative effects of Sal B to its effects on the phosphorylation site of Smad3 C‐terminal. This study emphasized that pSmad3C plays a significant role in the favorable effects of Sal B in combating liver cancer. In order to clarify the mechanisms by which Sal B acts, HepG2 cells were genetically modified by introducing three plasmids. These plasmids contained Smad3 WT (wild‐type Smad3 with intact phosphorylation site), Smad3 EPSM (mutated Smad3 with phosphorylation site mutation in the linker region), and Smad3 3S‐A (mutated Smad3 with phosphorylation site mutation in the C terminus). Indeed, upon cotreatment of these transfected HepG2 cells with Sal B at 50 pmol/L, there was marked reduction in cell proliferation and migration, while acceleration in apoptosis. In addition, the findings of the study indicated that treatment with Sal B modified the upregulation of pSmad3L in Smad3 3S‐A cells caused by TGF‐β1 and significantly boosted the upregulation of pSmad3C in TGF‐β1‐treated Smad3 EPSM–HepG2 cells. Furthermore, in the HepG2 cells transfected with the three different plasmid types, treatment with Sal B and TGF‐β1 impacted p21 protein expression similarly to pSmad3C protein expression and PAI‐1 and c‐Myc protein expression similarly to pSmad3L expression. Overall, this study found that the ameliorative effects of Sal B on hepatocarcinogenesis were due to the elevation of Smad3 phospho‐isoform conversion. Specifically, it shifted the pathway from the oncogenic JNK/pSmad3L/c‐Myc pathway to the tumor‐suppressive TpRI/pSmad3C/p21 pathway [130]. Moreover, an earlier study used HepG2 liver cancer cells, demonstrating the potential antitumor effects of *Stellera chamaejasme* L. (ESC) extracts on EMT induced by TGF‐β. It has demonstrated that ESC suppressed the EMT in HEP G2 liver cells by inhibiting Smad2 signaling pathway. The pretreatment of HepG2 cells with ESC (0.2–5 µg/mL) significantly reversed TGF‐β‐indued cell scattering, migration, invasion, inducing inhibition in vimentin, while upregulating E‐cadherin levels. Also, ESC was reported to reverse the nucleus translocation of Smad2 in hepG2 cells [131].

The neolignan ailanthoidol (ATD) has been identified by researchers as possessing antioxidant, anti‐inflammatory, and antiadipogenic effects. This extract is obtained from the bark of the Zanthoxylum ailanthoidol plant [132, 133, 134]. The effect of ATD on hepatoblastoma cell progression promoted by TGF‐β was explored by using human hepatoma HepG2 [135]. According to this study, the positive impacts of ATD on the progress of hepatoblastoma cells were ascribed to its capacity to inhibit two signaling pathways activated by TGF‐β1: the P38MARK pathway and the Smad 2/3 pathway. This inhibition resulted in the reduction of proteins related with the emergence of liver cancer. The findings of this study confirmed antitumor potential of ATD toward HepG2 hepatoblastoma cell progression induced by (10 ng/mL) of TGF‐β1 for 24 h. It was observed that the IC_50_ of ATD for 48 h on HepG2 cells was close to 100 µM. Cotreatment with ATD (25 and 50 µM) effectively suppressed cell invasion, colony formation, and reversed cell scattering of HepG2 cells. In addition, the inhibitory impact of ATD on the buildup of integrin α3, N‐cadherin, vimentin, and MMP2 caused by TGF‐β1, as well as the phosphorylation of both p38 MAPK and Smad2 signaling pathway, was detected in hepatoblastoma cells. This suggests that ATD has potential in reducing the severity of liver cancer.

Echinacoside (ECH) is the primary constituent of Cistanches Herba, a traditional Chinese medicinal herb sometimes referred to as “desert ginseng” [136]. The beneficial antitumor impacts of ECH on liver cancer was ascribed to its modulatory effects on modulation of the miR‐503‐3p/TGF‐β1/Smad axis in liver cancer [137]. ECH (5, 10, and 20 mg/mL) was found to markedly inhibit cell proliferation and induce apoptosis, as demonstrated by the decline induced in number, cell viability, and arrest of the S phase of HepG2 and Huh7 colony cells. Further, the suppressing effect of ECH on the TGF‐β1/Smad signaling pathway was demonstrated by reducing the expression levels of both TGF‐β1 and Smad3 protein levels while upregulating that of Smad7 expression.  It is important to highlight that miR‐503‐3p has been identified as a possible regulator of TGF‐β1. ECH was discovered to be involved in upregulating the expression levels of miR‐503‐3p in HepG2 and Huh7 cells, hence contributing to the hindering of cell invasion and migration.

Jiedu recipe (JR) is composed of valvate actinidia root, salvia chinensis, pseudobulbus cremastrae seu pleiones, and endothelium corneum gigeriae galli. An earlier study proposed the effect of JR on the inhibition of proliferation and metastasis of HCC [138]. According to the study, JR (0.1, 0.5, and 1 mg/mL) significantly inhibited both Smad‐dependent and Smad‐independent pathways, resulting in suppression of EMT caused by TGF‐β1 (10 ng/mL). It was found that JR (0.1, 0.5, and 1 mg/mL) significantly inhibited hepatoma cell proliferation and migration in SMMC‐7721 and Huh7 cells; that was verified by a notable decrease in cell viability and the number of migrated cells. JR also inhibited the phosphorylation of Smad2/3, Akt, ERK, JNK, and p38 MAPK, leading to suppression of EMT in HCC, as confirmed by an elevation in cadherin expression levels and decrease in expression levels of vimentin, N‐cadherin, and MMP2/9.

Solanine, a weakly basic glycoside, was reported to have a wide range of anticancer effects. Solanine has found to play a role in suppressing cell growth, triggering cell death, halting the cell cycle, promoting self‐degradation of cells, enhancing the effectiveness of chemotherapy and radiation therapy, preventing the transformation of cells from epithelial to mesenchymal, suppressing the migration of tumors, and inhibiting angiogenesis. Gao et al. [139] used HCC cell line H22 to demonstrate the protective impacts of solanine on TGF‐β expression. Notably, among the liver cancer cell lines, HepG2, SMMC‐7721, Hep3B, BEL‐7402, and H22, H22 cell line was proved to be the highest one in expression of TGF‐β, comparing with normal liver cells. Cotreated with solanine (0.4, 2, and 10 µmol/L) considerably reduced the amount of TGF‐β in the H22 cell line with a dose‐dependent relationship.

Table 3 summarizes in vitro effects of various natural compounds on the TGF‐β signaling pathway in hepatocellular carcinoma cell lines.

**TABLE 3:** In vitro effects of natural compounds on TGF‐β pathway in hepatocellular carcinoma cell lines.

Compound (chemical class)	Cancer cell lines	Tested concentrations	Mechanism/signaling pathways	Results (effect on cancer cells)	References
Chinese Dragon's blood EtOAc extract (polyphenol‐rich extract)	HepG2, SK‐HEP‐1	0, 10, 20, 40, 80 µg/mL	↓Smad3, ↑MAPK signaling, ↑p38, ERK, PARP phosphorylation, ↓N‐cadherin, Slug	↓Cell viability ↓migration ↓Invasion ↑Apoptosis	[118, 120]
Tanshinone IIA (diterpenoid quinone)	Bel‐7404, SMMC‐7721, Bel‐7402	0, 5, 10, 20, 40, 80, 160 µM	↓Smad2/3 phosphorylation, ↑Smad7, ↓N‐cadherin, ↓Ki67, ↓cyclin D1, ↓Bcl2	↓Cell proliferation ↓Migration ↓invasion ↑Apoptosis	[121]
Oroxylin A (flavonoid)	SMMC‐7721	50 µM	↓TGF‐β1/Smad signaling, ↑HDAC1 degradation, ↑NAG‐1, ↓N‐cadherin, ↓vimentin, ↓MMP 2, ↓MMP9, ↓Twist1, ↓PAI‐1	↓Cell migration ↓invasion ↑Apoptosis	[126]
Sulforaphane (isothiocyanate)	HepG2	10, 20, 40, 60, 80 µM	↓TGF‐β1‐induced EMT ↓vimentin, ↑E‐cadherin ↓Fibroblast‐like mesenchymal cells	↓Cell proliferation ↓Migration ↓Invasion ↑Apoptosis ↑Cell cycle arrest at G0/G1	[128]
Salvianolic acid B (polyphenol)	HepG2	50 pmol/L	↓Smad3 phosphorylation, ↑pSmad3C, ↓pSmad3L, ↑p21, ↓c‐Myc, ↓PAI‐1	↓Cell proliferation, ↓Migration ↑Apoptosis	[130]
*Stellera chamaejasme* L. extract (natural plant extract)	HepG2	0.2–5 µg/mL	↓Smad2 nuclear translocation, ↓Vimentin, ↑E‐cadherin	↓Cell scattering ↓migration ↓Invasion	[131]
Ailanthoidol (lignan)	HepG2	25, 50 µM	↓p38 MAPK and Smad2 signaling pathways, ↓integrin α3, ↓N‐cadherin, ↓vimentin, ↓MMP 2	↓Cell invasion, ↓colony formation, ↓Cell scattering	[135]
Echinacoside (phenylethanoid glycoside)	HepG2, Huh7	5, 10, 20 mg/mL	↑miR‐503‐3p, ↓TGF‐β1, Smad3, ↑Smad7	↓Cell proliferation ↓Viability ↓Migration ↑Apoptosis	[137]
Jiedu recipe (Traditional Chinese formula)	SMMC‐7721, Huh7	0.1, 0.5, 1 mg/mL	↓Smad2/3, Akt, ERK, JNK, p38 MAPK signaling, ↓N‐cadherin, vimentin, MMP2/9, ↑E‐cadherin	↓Cell proliferation, migration; suppression of EMT	[138]
Solanine (steroidal alkaloid)	H22, HepG2, SMMC‐7721, Hep3B, BEL‐7402	0.4, 2, 10 µmol/L	↓TGF‐β expression	↓Cell growth, ↑Apoptosis, ↑Cell cycle arrest	[139]

Abbreviations: ATD, ailanthoidol; CDBEE, Chinese Dragon's blood EtOAc extract; ECH, echinacoside; EMT, epithelial–mesenchymal transition; ERK, extracellular signal‐regulated kinase; ESC, Stellera chamaejasme L. extract; G0/G1, Gap 0/Gap 1 phase; HCC, hepatocellular carcinoma; HDAC1, histone deacetylase 1; HepG2, SK‐HEP‐1, SMMC‐7721, Bel‐7404, Bel‐7402, Huh7, various liver cancer cell lines; JR, Jiedu recipe; MAPK, mitogen‐activated protein kinase; miR‐503‐3p, microRNA 503‐3p; MMP2/9, matrix metalloproteinase 2/9; NAG‐1, nonsteroidal anti‐inflammatory drug‐activated gene 1; OXA, oroxylin A; PAI‐1, plasminogen activator inhibitor‐1; PARP, poly (ADP‐ribose) polymerase; pSmad3C, phosphorylated Smad3 C‐terminal; pSmad3L, phosphorylated Smad3 linker; SFN, sulforaphane; Smad, mothers against decapentaplegic homolog; TanIIA, tanshinone IIA; TGF‐β1, transforming growth factor‐beta 1.

#### 3.1.4 Colorectal Cancer

Oxyresveratrol is a plant‐based derivative of reservatrol that may inhibit human CRC cell migration by maintaining EMT through the regulation of the TGF‐β/SMAD signaling route. This pathway has anti‐inflammatory and cancer‐preventive roles in the early stages of inflammation or cancer, but promotes tumor development in the later stages as mentioned before, thus, oxyresveratrol affect TGF‐β/SMAD signaling pathway to inhibit cancer development; however, the exact mechanism is still not known in CRC. It is also showed that oxyresveratrol inhibits EMT by mediating Snail/E‐cadherin and by regulating TGF expression in HCT116 cells and TGF‐β induced HT‐29 cells. For HCT116 cells, after 24 h using 70 µM can significantly inhibit metastasis and after 48 h, this inhibition enhanced 17%; however, for TGF‐β induced HT‐29 cells, after 24 h, 70 µM can inhibit metastasis 14% and after 48 h inhibit metastasis by 30%.

Ursolic acid, a naturally occurring active chemical frequently used in traditional Chinese medicine, is tested on HCT116 and HCT‐8 cells. It has been shown to have potent anticancer activities against a number of malignancies. Previous research demonstrated that ursolic acid increased apoptosis in CRC cells while inhibiting cellular growth and angiogenesis. In addition, another investigation revealed that after 24 h of treatment with ursolic acid, the values for HCT‐8 and HCT116 cells were 25.2 and 37.2 µM, respectively. After 48 h, HCT116 and HCT‐8 cells exhibited 28.0 and 19.4 µM, respectively. The results showed a decrease in CRC cell invasion by modifying the TGF‐β1/zinc finger E‐box‐binding homeobox/miR‐200c signaling scheme [140].

SFN, an isothiocyanate compound, is predominantly present in the inert storage form as glucoraphanin, with broccoli being a primary source of this important compound, belonging to the Brassicaceae family [127]. Mechanical damage, such as biting, chewing, or slicing, to broccoli and other cruciferous vegetables results in the release and hydrolysis of glucoraphanin by the plant enzyme myrosinase, yielding the biologically active SFN. Exposure of myrosinase to high temperatures during cooking can lead to its degradation and loss of activity, thereby compromising SFN synthesis [141, 142]. Consequently, consuming raw cruciferous vegetables rather than cooking them can considerably enhance the bioavailability of SFN and its consequent therapeutic effects. To ensure optimal SFN production, it is advisable to consider natural extracts containing myrosinase‐activated glucoraphanin [142]. Regarding SFN anticancer activity, a study demonstrated that the reserve of cell growth by SFN is closely connected with a dose‐dependent reduction in the protein expression and enzymatic activity of the proto‐oncogenic ornithine decarboxylase. This outcome appears to stem from reduced levels of the c‐myc transcription factor and its transactivation activity, which directly regulates the expression of ornithine decarboxylase. These effects are attributed to SFN‐induced activation of the TGF‐β/Smad signaling pathway [143].

Baicalin, derived from *Scutellaria baicalensis* Georgi, has preventive properties against liver [144], nonalcoholic steatohepatitis [145], and gastrointestinal illnesses [144]. Baicalin's beneficial effect involves modulating downstream immune response and apoptosis pathways caused by oxidative damage and inflammation. Baicalin's therapeutic benefits on NAFLD/NASH and cholestasis due to the scavenging of ROS capabilities as baicalin is linked to important oxidative stress regulators such as PI3K/Akt/NRF2, heme oxygenase‐1 (HO‐1), Keap‐1, and NF‐κB. Also, it could alleviate that liver disorders by targeting IL‐1β, IL‐6, TNF‐α, MIP‐1α, and MIP‐2, as well as TGF‐β1/Smads, STAT3, and NF‐κB. Not only the liver disorders could be suppressed via baicalin but also CRC could be suppressed through its apoptotic roles via the regulation of Bax/Bcl‐2/caspase‐3/caspase‐9 pathway [144]. Regarding CRC, treatment with baicalin leads to G1 phase cell cycle arrest and induces cell death through a mechanism independent of p53. Additionally, it inhibits both endogenous and exogenous TGF‐β1‐induced EMT in CRC by disrupting the TGF‐β/Smad signaling pathway [146]. Lately, a study revealed that baicalin decrease exogenous and endogenous TGF‐β1‐induced EMT in CRC by hindering the TGF‐β/SMAD route in HCT‐166 cells and HCT‐116 cells using doses from 50 to 200 µM [144, 146].

Oxymatrine is the main ingredient of a Chinese herb Sophora flavescens Ait, that is widely used in traditional Chinese therapy [147]. It inhibits the formation of CRC and its migration through the ruling of the level of expression of TGF‐β1, Smad4, and pSmad2 by inhibiting P38‐dependent expression of PAI‐1. And these findings are based on a study that investigated the influence of oxymatrine on the division of RKO cells and discovered that after treating RKO cells with various concentrations of oxymatrine for 24, 48, and 72 h, the cytotoxicity of oxymatrine was assessed and demonstrated a decrease in RKO cell division in a concentration‐related and influenced by time means [148].

**FIGURE 4:**
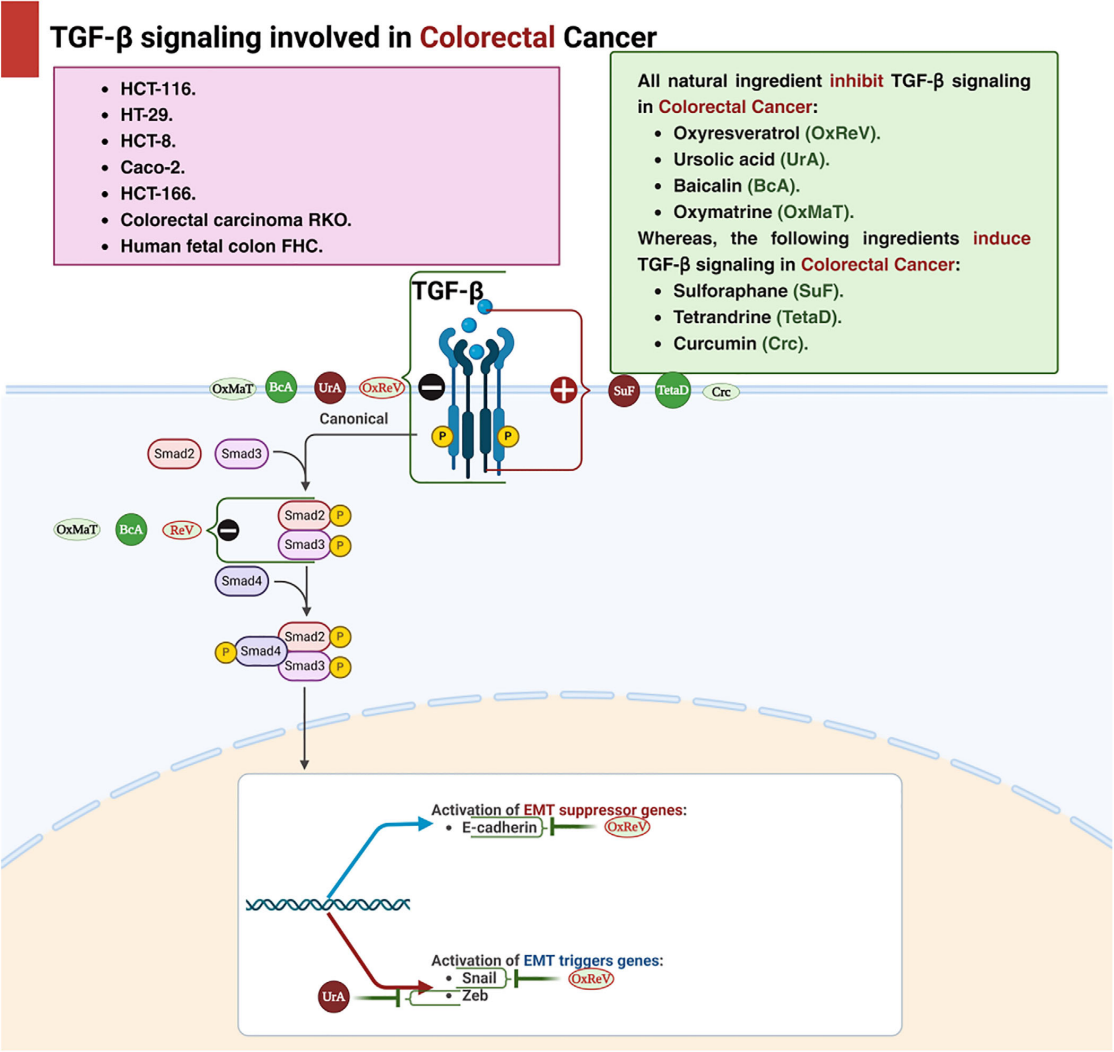
Natural products targeting TGF‐β pathway in treatment of colorectal cancer. This schematic illustrates the involvement of TGF‐β signaling in colorectal cancer and the effects of various natural compounds on this pathway. Colorectal cancer cell lines included in the study are HCT‐116, HT‐29, HCT‐8, Caco‐2, HCT‐166, colorectal carcinoma RKO, and human fetal colon FHC. The canonical TGF‐β signaling pathway involves Smad2/3 phosphorylation, forming a complex with Smad4, which translocates to the nucleus to regulate gene expression. Natural ingredients that inhibit TGF‐β signaling in colorectal cancer include oxyresveratrol (OxReV), ursolic acid (UrA), baicalin (BcA), and oxymatrine (OxMaT). These compounds contribute to the activation of EMT suppressor genes like E‐cadherin and inhibition of EMT trigger genes such as Snail and Zeb. Conversely, compounds like sulforaphane (SuF), tetrandrine (TetaD), and curcumin (Crc) induce TGF‐β signaling, influencing various cellular processes. BcA, baicalin; Crc, curcumin; E‐cadherin, epithelial cadherin; EMT, epithelial–mesenchymal transition; HCT‐116, HT‐29, HCT‐8, Caco‐2, HCT‐166, RKO, FHC, colorectal cancer cell lines; OxMaT, oxymatrine; OxReV, oxyresveratrol; Smad, mothers against decapentaplegic homolog; Snail, zinc finger protein SNAI1; SuF, sulforaphane; TetaD, tetrandrine; TGF‐β, transforming growth factor‐beta; UrA, ursolic acid; Zeb, zinc finger E‐box‐binding homeobox.

Tetrandrine, a bisbenzylisoquinoline alkaloid derived from Stephania tetrandra, has traditionally been used as an antihypertensive and anti‐inflammatory agent, with recent studies also indicating its cardioprotective effects [149]. In addition, tetrandrine showed an anticancer activity regarding CRC as it suppressed the growth of colon cancer IL‐6 induced HCT116 cells and triggered apoptosis by upregulating the expression of TGF‐β1 through the downregulation of the MMP‐2 activity and restoration of the E‐cadherin gene promoter activity [149, 150]. And in another research, it has been revealed to diminish the signaling pathway PI3K/Akt by the upregulation of TGF‐β1 and the reduction of PTEN phosphorylation [151] (Table 4 and Figure 4).

**TABLE 4:** In vitro effects of natural compounds on TGF‐β pathway in colorectal cancer cell lines.

Compound (chemical class)	Cancer cell lines	Tested concentrations	Mechanism/signaling pathways	Results (effect on cancer cells)	References
Oxyresveratrol (polyphenol)	HCT116 HT‐29	70 µM	↓EMT ↓Snail/E‐cadherin regulates TGF‐β/Smad signaling	↓Metastasis by 17% in HCT116 after 48 h ↓Metastasis by 30% in HT‐29 after 48 h	[140]
Ursolic acid (triterpenoid)	HCT116, HCT‐8	25.2, 37.2 µM (24 h); 28.0, 19.4 µM (48 h)	↑Apoptosis, ↓cell growth, angiogenesis, modifies TGF‐β1/ZEB/miR‐200c signaling	↓Cell invasion	[140]
Sulforaphane (isothiocyanate)	Not specified (CRC)	Not specified	Activates TGF‐β/Smad signaling, ↓c‐myc transcription factor	↓Cell growth by reducing ornithine decarboxylase activity	[143]
Baicalin (flavonoid)	HCT‐166, HCT‐116	50–200 µM	Modulates Bax/Bcl‐2/caspase pathway, inhibits TGF‐β/Smad signaling	G1 phase cell cycle arrest ↑Apoptosis ↓EMT	[144, 146]
Oxymatrine (alkaloid)	RKO	Not specified	Inhibits P38‐dependent expression of PAI‐1, regulates TGF‐β1, Smad4, pSmad2	↓Cell division ↓Migration	[148]
Tetrandrine (alkaloid)	HCT116	Not specified	↑TGF‐β1, ↓MMP‐2 activity, ↑E‐cadherin gene promoter activity, ↓PI3K/Akt signaling	↓Cell growth ↑Apoptosis ↓PTEN phosphorylation	[149, 150]

Abbreviations: CRC, colorectal cancer; EMT, epithelial–mesenchymal transition; HCT116, HT‐29, HCT‐8, HCT‐166, RKO, various colorectal cancer cell lines; MMP‐2, matrix metalloproteinase‐2; PAI‐1, plasminogen activator inhibitor‐1; PI3K/Akt, phosphoinositide 3‐kinase/protein kinase B; PTEN, phosphatase and tensin homolog; SFN, sulforaphane; Smad, mothers against decapentaplegic homolog; TGF‐β1, transforming growth factor‐beta 1; ZEB, zinc finger E‐box‐binding homeobox.

#### 3.1.5 Prostate Cancer

Thymoquinone (TQ), a prominent component found in the cells of black seed oil (*Nigella sativa*), has been demonstrated to have anticancer properties by suppressing the TGF‐β/Smad2/3 signaling pathway. Many research provided multiple pieces of evidence showing that TQ effectively stopped the process of EMT in PCa. TQ effectively prevents the growth of PCa cells when present at cellular concentrations of 10 µM or higher, inhibiting cell proliferation by more than 90%. Moreover, the antimetastatic effect was demonstrated by the substantial suppression of both the migratory and invasive capacities of PCa. The efficacy of TQ in inhibiting EMT was demonstrated through the utilization of two sophisticated methodologies: polymerase chain reaction (PCR) and western blotting. TQ at concentrations of 2.5, 5.0, and 10 µM for 24 h increased the mRNA expression and protein levels of E‐cadherin in a manner that depended on the concentration. In parallel, mRNA expression and protein levels of vimentin and Slug were markedly reduced. To confirm the effectiveness of the anti‐TGF‐β action, Kou et al. [152] used two different methods. The classical group exhibited a significant decrease in mRNA expression and protein levels of TGF‐β, Smad2, and Smad3 in the presence of TQ. The second method involved introducing TGF‐β into DU‐145 and PC3 cells using plasmid transfection. Remarkably, TQ completely counteracted all effects of TGF‐β in the transfected cells. The antimetastatic effect of TQ is primarily mediated by the TGF‐β/Smad2/3 signaling pathway [152].

Nutraceuticals like curcumin reduce cancer progression. In an encouraging study, Katta et al. [153] investigated the gene expression responses linked with curcumin in two types of PCa cell lines: hormone‐responsive (LNCaP) and hormone‐independent (C4‐2B), which are highly metastatic. Curcumin administration had the most significant effect at 12 h. Additionally, most affected genes reverted to regular expression within 24–48 h posttreatment. Katta et al. [153] used Genomatix Pathway system analysis to identify genes affected by short‐term curcumin therapy. A total of 19 genes were upregulated: HO‐1, Ewing sarcoma breakpoint region 1, cyclic AMP‐dependent transcription factor, sestrin‐2, ferritin heavy chain 1 (FTH1), glutamate–cysteine ligase modifier subunit, AF4/FMR2 family member 4, E74 like ETS transcription factor 3, RIT1, DRE1, DDIT3, cytoplasmic polyadenylation element binding protein 4 (CPEB4), CAMP responsive element modulator (CREM), Baculoviral IAP repeat‐containing protein 4 (BIRC4), RIO kinase 3, phosphoinositide‐3‐kinase regulatory subunit 3, ubiquitin conjugating enzyme E2 H, and chromosome 6 open reading frame 62. On the other hand, 12 genes were downregulated: DEAD/H‐box helicase 11, carnitine O‐octanoyltransferase, prostate transmembrane protein, androgen induced 1 (PMEPA1), hypoxia‐inducible gene 2, chromosome 1 open reading frame 116, aurora kinase B, DNA fragmentation factor subunit beta, glypican‐6, chromosome 15 open reading frame 20, adrenoceptor beta 2, solute carrier family 16 member 6, and MYC proto‐oncogene, basic helix‐loop‐helix transcription factor (MYC). Network analysis showed that curcumin‐treated cells had downregulated MYC, the gene with the most significant genetic connections with other genes. HMOX1 and ATF‐3, the other two prevalent genes, were upregulated in both cells. Curcumin treatment in LNCaP cells impacted signaling pathways, revealing three downregulated genes (RAF1, BCL6, IGF1R) and two upregulated genes (PTEN, EGFR1). In curcumin‐treated LNCaP cells, significant alterations were observed in the signaling pathways governing apoptosis, cell cycle arrest, stress response genes, and DNA‐dependent transcription. Curcumin‐treated C4‐2B cells showed four downregulated genes: SOX4 (SRY (sex determining region Y)‐Box 4, EGFR, WT1 (Wilms tumor 1), E2F2 (E2F transcription factor 2), and one upregulated gene: MALAT1 (metastasis associated lung adenocarcinoma transcript 1). According to pathway analysis, apoptosis, cell cycle, and G1/S mitotic cell cycle pathways in C4‐2B cells were the most affected signaling pathways. Katta et al. [153] found that the administration of curcumin resulted in the activation of BMP receptor signaling, PTEN‐mediated cell cycle arrest, induction of apoptosis, and enhancement of cell–cell adhesion pathways, while downregulating TGF‐β receptor, WNT, AP‐1 transcription factor networking, NF‐κB, and PI3K/Akt/mTOR pathways in LNCaP cells. Curcumin treatment suppressed TGF‐β receptor signaling, Wnt signaling, and FOXM1 transcription factor network in metastatic C4‐2B cells while upregulating IL‐6, PTEN‐dependent cell cycle arrest, apoptosis, and G_2_/M checkpoint pathways. Both less aggressive LNCaP and highly metastatic C4‐2B cells showed downregulation of the TGF‐β receptor signaling pathway after 48 h of therapy. After 48 h of curcumin administration, only four genes (FTH1, CPEB4, C6ORF61, and PMEPA1) were discovered to be regulated, indicating their long‐term impact. The genes (FTH1, CPEB4, and C6ORF61) were upregulated, and the PMEPA1 gene was downregulated [153].

In this study, Liu et al. [154] investigated how red clove (RC) isoflavones affect the protein expression of dehydro‐epiandrosterone (DHEA) metabolic enzymes in 6S stromal cells. RC isoflavones completely suppressed the expression of 3β‐HSD AGG protein induced by TGF‐β1, while RC isoflavones did not reduce the expression of HSD‐17 β1 protein induced by TGF‐β1. The RC isoflavones possess molecular structures that closely resemble those of steroid hormones. They can bind to DHEA metabolic enzymes, impacting their stability and functions. Nevertheless, the introduction of 4‐DIONE to 6S cells for 48 h did not modify the production of testosterone, regardless of the presence or absence of RC isoflavones. This indicates that the protein expression and activity of HSD‐17b5 remained unaltered by RC isoflavones. Via nongenomic mechanisms, Liu et al. [154] determined that the modulation of protein expression of 3β‐HSD AGG and HSD‐17β1 by TGF‐β1 and RC occurs. Liu et al. [154] discovered interactions between TGF‐βRs and 3b‐HSD AGG or HSD‐17b1AGG in 6S cells, both in their natural state and when treated with RC isoflavones alone. However, when TGF‐β1 is present, it prevents these interactions from occurring. In addition, the administration of RC therapy counteracted the suppressive impact of TGF‐β1 on the connections between TGF‐βRs and 3β‐HSD AGG, but not with HSD‐17b1 AGG [154].

Metabolites of hesperidin include hesperetin. Hesperidin is an orange and lemon flavanone. Hesperetin affected apoptosis in silico and in vitro in PCa studies. However, hesperetin's effects have yet to be compared with other metastatic PCa factors. The characteristics of hesperetin revealed its impact on TGF‐β‐induced invasion and migration in PC3 cells. According to Dalpatraj et al. [155], hesperetin lowers PC3 cell viability and morphology at doses ranging from 50 to 500 µM for 24 and 48 h. Additionally, hesperetin significantly reduces PC3 cell viability, even after TGF‐β stimulation. Using 200 µM hesperetin dramatically lowers cell viability to about 50% in TGF‐β‐induced PC3 cells. Flow cytometry research reveals that hesperetin inhibits TGF‐β‐induced PC3 cell cycle in the S and G_2_/M stages. Nearly 50% of hesperetin‐treated cells were arrested in both phases. Hesperetin's influence on cell migration was examined by Dalpatraj et al. [155] using the scratch assay. Interestingly, hesperetin decreased TGF‐β‐induced PC3 cell migration and limited scratched wound closure by at least 20%. Matrigel invasion assay indicated that hesperetin inhibited PC3 cell invasion by 50%. To determine the TGF‐β signaling pathway, Dalpatraj et al. [155] found a decrease in *p*‐smad3 protein levels and an increase in *p*‐c‐Jun levels. N‐ and E‐cadherin mRNA transcription and protein levels were assessed for confirmation. N‐ and E‐cadherin levels plummeted and rose, respectively. Hesperetin primarily impacts TGF‐β signaling via the typical Smad cascade. H3K4me3 and H3K27me3 levels increased after hesperetin treatment, raising concerns regarding histone methylation. When given with TGF‐β, hesperetin lowered H3K9me3 levels, but when given alone, it considerably boosted them [155].

Quercetin exhibits diverse anticancer properties, including reduction of cell growth, kinase activity, apoptosis induction, differentiation, and suppression of MMPs secretion. Additionally, there have been reports indicating a decrease in the adherence of tumor cells, their invasive behavior, metastasis, and angiogenesis. Therefore, Baruah et al. [156] were motivated to assess the influence of quercetin on phenomena such as tumor advancement and infiltration. Baruah et al. [156] demonstrated that the growth of PC3 cells was suppressed in a manner that depended on both the dosage (ranging from 5 to 60 µM) and the duration of exposure (24, 48, and 72 h). Quercetin has a notable impact on every phase of the cell cycle, causing a halt in cell advancement and proliferation and finally leading to an elevated rate of cell death in the population. In addition, it was observed that quercetin triggers cell death by activating caspase‐3 at doses of 20 and 25 µM after 24 h of treatment. Remarkably, annexin V labeling revealed a noteworthy rise in the proportion of apoptotic cells as the period (24 and 48 h) and dosage (20 and 25 µM) of quercetin increased. Baruah et al. [156] utilized a wound‐healing experiment to demonstrate that quercetin effectively inhibited cell migration, resulting in the absence of gap closure.

Furthermore, the capacity of PC3 to form colonies was significantly suppressed by the presence of quercetin (20 µM). To comprehend the fundamental process via which quercetin controlled EMT, Baruah et al. [156] employed real‐time PCR to quantify the transcriptional repressors Twist, Snail, and Slug as important components of the Wnt signaling pathway. It was shown that quercetin effectively reduced the expression of all these indicators. The real‐time PCR technique was utilized to detect the genetic expression of N‐cadherin, E‐cadherin, and vimentin in relation to EMT. Quercetin restored the expression of E‐cadherin while simultaneously decreasing the expression of N‐cadherin and vimentin. Quercetin therapy resulted in the downregulation of β‐catenin and cyclin D1, as the real‐time PCR data demonstrated [156].

Regarding extracellular remodeling components, MMP‐9 expression was reduced by exposure to quercetin. Regarding IAP proteins, it was found that quercetin exposure resulted in a decrease in the expression of survivin. To assess the tumor's capabilities under low oxygen settings, the administration of quercetin resulted in a considerable decrease in the expression of HIF‐1α. This reduction depended on the dosage and duration of therapy [156].

A promising PCa anti‐EMT trial by Pollard et al. [157] examined cardenolide medicines (digitoxin and oleandrin). This study included in vitro, transplanted PAIII tumor cells into Lobund–Wistar rats and bioinformatic analysis, proving the results’ validity. According to Pollard et al. [157], both cardenolide medicines reduce NF‐κB signaling in PC3 and Hela cells, affecting baseline and TNF‐α‐dependent processes. Concerning EMT signaling, 10 and 25 nM digitoxin dramatically inhibit vimentin expression while increasing E‐Cadherin expression. EMT signaling reversals via digitoxin are dose dependent and statistically significant at 25 nM concentration. Digitoxin decreases PC3 cell survival, growth rate, and TGF‐βR2 protein expression, regardless of TNF‐α administration. Surprisingly, digitoxin inhibits TNF‐α/NF‐κB signaling in PC3 cells, TGF‐βR2 signaling, and EMT signaling via E‐cadherin and vimentin. Digitoxin decreases PC3 cell density and may alter its structure. Also, RNA sequencing analysis was used to establish the impact of digitoxin on HSPB1 and RBFOX2 mRNA expression and ratio. Interestingly, digitoxin reduces both HSPB1 and the HSPB1/RBFOX2 ratio, supporting the idea that it suppresses EMT in human PC3 cells [157]. The main effect of digitoxin on targeted genes is an inflammatory response, as shown by a gene ontology study. Gene ontology analysis for digitoxin shows granulocyte/agranulocyte adhesion and diapedesis, leukocyte extravasation, circadian rhythm, tight junction (EMT), and cAMP‐mediated signaling. In addition, Pollard et al. [157] identified 17 NF‐κβ‐driven genes using digitoxin. Digitoxin suppresses all 14 except Ada. Additionally, digitoxin significantly reduced TGF‐βR2 mRNA levels in rat tumors. Digitoxin inhibition suggests a functional similarity between NF‐κβ signaling to TGF‐βR2 in prostate PC3 cancer cells and NF‐κβ signaling. Intriguingly, Pollard et al. [157] found nine EMT‐associated mRNA genes out of 101. Only seven mRNAs are decreased by digitoxin. Besides TGF‐βR2, they include Zeb2, Timp3, Cdh11, TGF‐βR2, WT1, Axl, and Epas1. Aldh1a and Sfrp4 are two of nine digitoxin‐induced mRNAs [157].

Osthole or 7‐methoxy‐8‐(3‐methyl‐2‐butenyl) coumarin inhibits cancer growth and metastasis. According to Wen et al. [158], osthole concentration in the 20–80 µM range inhibited wound‐closure and invasive abilities. Furthermore, osthole did not affect vitality after 24‐h treatment at the highest dosage of 80 µM. Treatment of DU145 cells with osthole (60 µM) for 24 h did not change apoptotic rates concerning the cell cycle. Osthole decreased cancer cell invasion at a non‐ or low‐cytotoxic concentration, showing that it inhibits AIPC cell motility. Osthole treatment in DU145 cells led to increased expression of (E‐cad and β‐catenin) and decreased (N‐cadherin). However, neither DU‐145 nor PC3 cells showed any effect on the activities of ECM degradation‐related proteins (MMP‐2 and MMP‐9). Osthole therapy drastically lowered Snail expression in DU145 cells but had little or no effect on Twist and Slug. Osthole also lowered Snail binding to the E‐cad promoter in DU145 cells. Osthole treatment of Du145 cells for 4–12 h reduced TGF‐β expression at the 4th hour and Akt, JNK1/2, and ERK1/2 at the 8th hour. Osthole's inhibitory action against JNK1/2 and ERK1/2 activities was amplified by pretreatment of Du145 cells with Ly294002, a PI3K/Akt inhibitor, demonstrating that Akt signals the JNK and ERK pathways. Wen et al. [158] found that osthole inhibited the transcriptional repressor of E‐cadherin, Snail, and cell‐invasive capacity in AIPC cells via inhibiting the TGF‐β/Akt/MAPK signaling cascade. Based on the miRNA study, osthole therapy downregulated miR‐146a, ‐22‐3p (most impacted), and ‐23a‐3p, which may target E‐cad. In Du145 cells, osthole suppresses miR‐23a‐3p and inhibits E‐cad production by binding to the human E‐cad gene's 3′UTR. According to Wen et al. [158], osthole may reduce AIPC cell motility via decreasing TGF‐β‐mediated miR‐23a‐3p expression.

Extensive research has consistently demonstrated the significant anticancer properties of genistein, a highly prevalent bioactive isoflavone molecule found in soy products, particularly concerning PCa. Paradoxically, lower doses of genistein, which are considered important for nutrition, can potentially stimulate cancer growth and metastasis. The proliferation of PC3 cells exhibited a notable rise at a concentration of 1 µM of genistein, reaching its maximum level at 10 µM. However, at genistein concentrations exceeding 10 µM, the proliferation rate saw a sharp decline. Terzioglu‐Usak et al. [159] found that the impact of genistein on cell migration did not diminish uniformly across all concentrations. Cell migration was considerably inhibited at 24 and 48 h when exposed to 10‐ and 50 µM genistein doses. Moreover, the suppressive impact of genistein was even more noticeable after 48 h of therapy. In relation to the advancement of the cell cycle, Terzioglu‐Usak et al. [159] discovered that the expression of CDK4 was increased at concentrations of 0.5 and 2.5 µM but decreased at concentrations of 5 and 10 µM of genistein. Furthermore, the genes responsible for cell proliferation, RPS6KA1 and RPS6KA2, decreased expression at a concentration of 0.5 µM. The expression of RPS6KA3 and 4 was increased at a dose of 2.5 µM of genistein. Curiously, MAPK3 was reduced at normal levels (0.5–5 µM), but MAPK8 was reduced at a dosage of 50 µM. MAPK14 and TAF1 were downregulated only at a concentration of 0.5 µM. Genistein at concentrations of 10 and 50 µM downregulated HIPK 2, another factor influencing cell growth. TGF‐βR1B and 2B expression was significantly reduced by 50 µM genistein. The ligands ACVR1 and BMPR1B were only downregulated by 50 µM genistein. Regarding apoptotic signals, it was shown that CSNK2A1 was only increased at a concentration of 50 µM genistein, whereas CSNK2A2 was increased at concentrations of 0.5, 5, and 10 µM of genistein. ATM increased at 5 and 50 µM, but ATR was only upregulated at 50 µM. CHUK gene expression was increased at a concentration of 50 µM genistein but decreased at concentrations of 2.5, 5, and 10 µM genistein. ABL1 was significantly increased at doses of 0.5, 2.5, and 50 µM of genistein. PRKDC exhibited a decrease in expression at a dose of 0.5 µM and an increase in expression at a concentration of 5 µM of genistein. In general, the effects of genistein on PC3 cells can be categorized into two phases based on the dosage: physiological (0.5, 2.5, 5, and 10 µM) and pharmacological (50 µM) concentrations of genistein [159].

Apigenin was tested in PC3 and bone metastasis androgen‐refractory LNCaP cells by Mirzoeva et al. [160]. Initially, apigenin reduced TGF‐β1‐induced VEGF expression at 25 and 50 µM. Additionally, apigenin (25 and 50 µM) reduces TGF‐β1‐induced Smad2/3 phosphorylation and nuclear translocation. Apigenin inhibited VEGF production less when Smad2 or Smad3 proteins were knocked down. According to Mirzoeva et al. [160], apigenin suppressed Smad2/3‐mediated VEGF expression. In addition apigenin at 25 and 50 µM reduced TGF‐β1‐induced c‐Src phosphorylation at Tyr416 in PC3‐M and LNCaP C4‐2B cells. Apigenin reverses TGF‐β1‐induced activation of c‐Src substrates (FAK and Akt). Apigenin dose‐dependently reduced FAK and Akt phosphorylation on Tyr576/577 and Ser473. Mirzoeva et al. [160] found that c‐Src inhibits the TGF‐β/ Smad2/3/VEGF pathway by blocking apigenin's effects in cells.

Silibinin, derived from the seed extract of milk thistle, is widely employed as a hepatoprotective treatment for acute and chronic conditions. In their study, Ting et al. [161] proposed that after 72 h, silibinin could lower IL‐6 levels in a conditioned medium dependent on the dosage. In addition, PrSCs were treated with silibinin at concentrations of up to 100 µM for 24 h. This treatment led to statistically insignificant reductions in cell counts and death. Remarkably, silibinin at concentrations ranging from 30 to 90 µM effectively suppresses the expression of α‐SMA in PrSCs induced by controlled conditioned media (CCM). Silibinin eliminated the activation of α‐SMA induced by CCM and TGF‐β1, reducing it to its original baseline levels. Silibinin significantly reduced the migration of PrSCs produced by CCM.

Furthermore, the expression of both constitutive and TGF‐β1‐induced α‐SMA was entirely inhibited by silibinin. Employing a cell viability experiment showed that treatment with silibinin at a concentration of 90 µM led to a marginal decrease in cell count and an increase in cell death. Ting et al. [161] observed that the presence of TGF‐β2 in CCM was reduced in a dose‐dependent manner by silibinin in PrSCs and DU‐145. TGF‐β2 expression was either undetectable or very low in the LNCaP, C4‐2B, and 22Rv1 PCa cell lines. Remarkably, the activation of α‐SMA is eliminated 2 h after the addition of TGF‐β2 when treated simultaneously with silibinin. Based on these findings, it is evident that silibinin has a time‐dependent effect on the suppression of α‐SMA activation in PrSCs. In summary, the administration of silibinin prevented the transition of prostate stromal cells into a CAF by explicitly targeting the production of TGF‐β2 in PCa cells. The administration of silibinin resulted in a direct reduction of α‐SMA expression in CAF cells [161].

**FIGURE 5:**
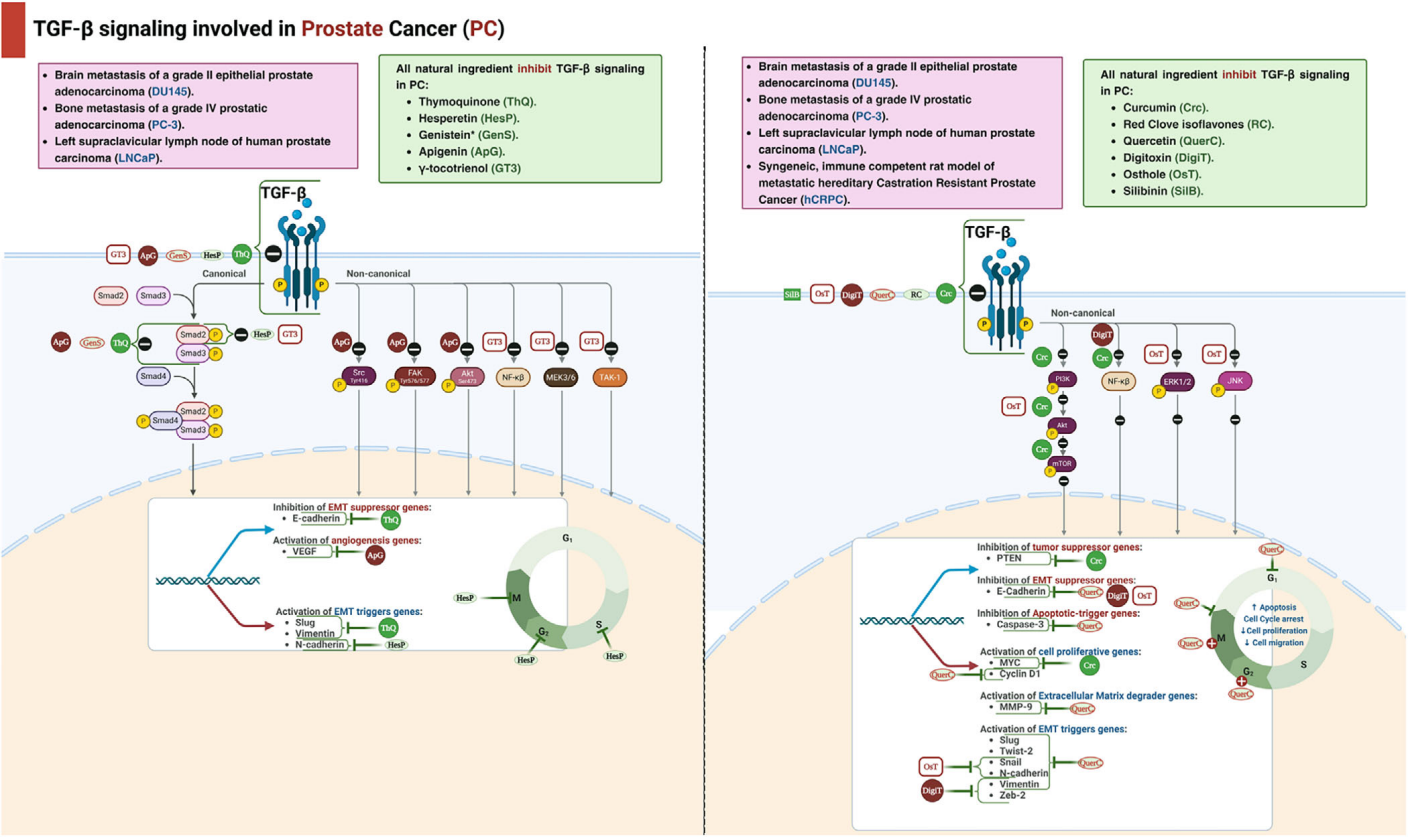
Natural products targeting TGF‐β pathway in treatment of prostate cancer. This figure shows the impact of various natural products on the TGF‐β signaling pathway in prostate cancer (PC). Panel A includes cell lines DU145, PC‐3, and LNCaP, and highlights compounds thymoquinone (ThQ), hesperetin (HesP), genistein (GenS), apigenin (ApG), and γ‐tocotrienol (GT3) that inhibit TGF‐β signaling. These compounds affect EMT suppressor genes like E‐cadherin and EMT trigger genes like slug, vimentin, and N‐cadherin, angiogenesis genes like VEGF, and key signaling molecules like Src, FAK, Akt, NF‐κB, MEK3/6, and TAK‐1. Panel B continues with additional cell lines including a rat model of metastatic hereditary castration‐resistant prostate cancer (hCRPC) and shows compounds curcumin (Crc), red clove isoflavones (RC), quercetin (QuerC), digitoxin (DigiT), osthole (OsT), and silibinin (SilB). These compounds inhibit tumor suppressor genes like PTEN, apoptotic trigger genes like caspase‐3, cell proliferative genes like MYC and cyclin D1, extracellular matrix degrader genes like MMP‐9, and EMT trigger genes. Akt, protein kinase B; ApG, apigenin; Crc, curcumin; DigiT, digitoxin; DU145, brain metastasis of grade II epithelial prostate adenocarcinoma; E‐cadherin, epithelial cadherin; EMT, epithelial–mesenchymal transition; FAK, focal adhesion kinase; GT3, γ‐tocotrienol; HesP, hesperetin; hCRPC, metastatic hereditary castration‐resistant prostate cancer; LNCaP, left supraclavicular lymph node of human prostate carcinoma; MEK3/6, mitogen‐activated protein kinase 3/6; NF‐κB, nuclear factor kappa‐light‐chain‐enhancer of activated B cells; N‐cadherin, neural cadherin; OsT, osthole; PC, prostate cancer; PC‐3, bone metastasis of grade IV prostatic adenocarcinoma; PTEN, phosphatase and tensin homolog; QuerC, quercetin; RC, red clove isoflavones; SilB, silibinin; Slug, zinc finger protein SNAI1; Smad, mothers against decapentaplegic homolog; Src, proto‐oncogene tyrosine‐protein kinase src; TAK‐1, transforming growth factor beta‐activated kinase 1; TGF‐β, transforming growth factor‐beta; ThQ, thymoquinone; VEGF, vascular endothelial growth factor; vimentin, intermediate filament protein.

Micronutrient antioxidants, including vitamin E, help eliminate harmful free radicals and potentially prevent oxidative harm to the prostate epithelium [162]. In their study, Campbell et al. [163] found that the γ and δ isoforms of tocopherol exhibited more efficacy in inhibiting growth than AT. All examined vitamin E isoforms exhibited enhanced growth inhibition when the concentration and duration were increased. A concentration of 80 µM was necessary to accomplish suppression of proliferation using AT. Among the tocopherols, DT exhibited more extraordinary performance than AT and GT, consistently achieving maximal growth inhibition at all tested timeframes and doses. Remarkably, α‐tocopherol stimulates the proliferation of LNCaP, while the γ and δ isoforms elicit growth suppression. Furthermore, γ‐tocotrienol explicitly suppresses cell proliferation in PC3. Remarkably, γ‐tocotrienol regulates the activity of the PPAR‐γ nuclear receptor in PC3 cells. However, GT3 does not directly bind to the PPAR‐γ receptor. γ‐Tocotrienol can increase the production of 15‐S‐HETE (a ligand for PPAR‐γ) and enhance the genetic expression of 15‐LOX. According to Campbell et al. [163], the growth inhibition caused by γ‐tocotrienol partly depends on PPAR‐γ. Furthermore, γ‐tocotrienol suppresses the production of TGF‐β2 in PC3 cells. Furthermore, at 24 h, the concentration of 3 µM γ‐tocotrienol resulted in the downregulation of p‐Smad2, while the total Smad remained steady. These downregulatory effects occurred downstream of TGF‐β2. Afterward, γ‐Tocotrienol prevents NF‐κB activation. In their study, Campbell et al. [163] discovered that nuclear total p38 and phosphorylated p38 levels were reduced. When PC3 cells are exposed to 5 µM GT3 for 24 h, the levels of MKK3/6 and TAK‐1 are reduced [163] (Table 5 and Figure 5).

**TABLE 5:** In vitro effects of natural compounds on TGF‐β pathway in prostate cancer cell lines.

Compound (chemical class)	Cancer cell lines	Tested concentrations	Mechanism/signaling pathways	Results (effect on cancer cells)	References
Thymoquinone (quinone derivative)	DU‐145 PC3	2.5, 5.0, 10 µM	↓TGF‐β/Smad2/3 ↑E‐cadherin ↓Vimentin ↓Slug	↓Cell proliferation, ↓Migration, ↓Invasion; ↑E‐cadherin	[152]
Curcumin (polyphenol)	LNCaP C4‐2B	Not specified	↓TGF‐β receptor, ↓Wnt ↓AP‐1, ↓NF‐κB ↓PI3K/Akt/mTOR ↑PTEN BMP receptor signaling	↑Apoptosis, ↑cell cycle arrest, ↓Cell–cell adhesion; ↓MYC, ↓TGF‐β receptor	[153]
Red clover isoflavones (isoflavonoid)	6S stromal cells	Not specified	Suppresses 3β‐HSD AGG protein induced by TGF‐β1 No effect on HSD‐17β1	Counteracts suppressive effect of TGF‐β1 on 3β‐HSD AGG, no effect on testosterone	[154]
Hesperetin (flavanone)	PC3	50–500 µM	↓p‐smad3, ↑p‐c‐Jun ↓N‐cadherin, ↑E‐cadherin affects cell cycle at S and G2/M stages	↓Cell viability, migration, invasion; cell cycle arrest	[155]
Quercetin (flavonoid)	PC3	5–60 µM	Activates caspase‐3, ↓β‐catenin, ↓cyclin D1, ↓N‐cadherin, ↓vimentin ↑E‐cadherin	↓Cell proliferation, migration, colony formation ↑Apoptosis	[156]
Digitoxin (cardiac glycoside)	PC3 Hela	10, 25 nM	Inhibits NF‐κB, TGF‐βR2, ↑E‐cadherin, ↓vimentin, ↓HSPB1 ↓HSPB1/RBFOX2 ratio	↓Cell survival, growth rate, migration, ↓invasion ↓EMT	[157]
Osthole (coumarin derivative)	DU145	20–80 µM	↓TGF‐β/Akt/MAPK, ↓Snail, ↓miR‐23a‐3p; ↑E‐cadherin, ↑β‐catenin	↓Cell invasion ↓Migration No effect on cell viability	[158]
Genistein (isoflavone)	PC3	0.5–50 µM	↓TGF‐βR1B, TGF‐βR2B, ACVR1, BMPR1B, ↑ATM, ATR, ABL1, CSNK2A1, CSNK2A2, PRKDC	↓Cell proliferation, migration; affects cell cycle genes	[159]
Apigenin (flavone)	PC3 LNCaP	25, 50 µM	↓Smad2/3 phosphorylation, nuclear translocation, c‐Src, FAK, Akt phosphorylation	↓VEGF production ↓Cell migration ↓Invasion	[160]
Silibinin (flavonolignan)	DU‐145 PrSCs	30–90 µM	↓α‐SMA, TGF‐β2	↓Cell migration, CAF formation; no significant effect on cell viability	[161]
γ‐Tocotrienol (tocotrienol, vitamin E derivative)	PC3 LNCaP	3–80 µM	↓TGF‐β2, ↓p‐Smad2, ↓NF‐κB, ↓p38, ↓MKK3/6, •TAK‐1	↓Cell proliferation ↓Survival ↓Growth rate	[163]

Abbreviations: ABL1, ABL proto‐oncogene 1; ACVR1, activin a receptor type 1; Akt, protein kinase B; AP‐1, activator protein 1; ATM, ataxia telangiectasia mutated; ATR, ataxia telangiectasia and Rad3‐related; AURKB, aurora kinase B; BCL, B‐cell lymphoma; BIRC4, baculoviral IAP repeat containing 4; BMP, bone morphogenetic protein; C6orf62, chromosome 6 open reading frame 62; CAF, cancer‐associated fibroblast; CPEB4, cytoplasmic polyadenylation element binding protein 4; CSNK2A1, casein kinase 2 alpha 1; CSNK2A2, casein kinase 2 alpha 2; DU145, LNCaP, PC3, C4‐2B, 6S stromal cells, PrSCs, prostate cancer cell lines; E‐cadherin, epithelial cadherin; EGFR, epidermal growth factor receptor; EMT, epithelial–mesenchymal transition; FAK, focal adhesion kinase; FTH1, ferritin heavy chain 1; GT3, gamma‐tocotrienol; HSPB1, heat shock protein beta‐1; HSPB1/RBFOX2, ratio of HSPB1 to RBFOX2; MAPK, mitogen‐activated protein kinase; MKK3/6, mitogen‐activated protein kinase 3/6; MMP, matrix metalloproteinase; NF‐κB, nuclear factor kappa‐light‐chain‐enhancer of activated B cells; PC3, DU‐145, LNCaP, PrSCs, prostate cancer cell lines; PI3K, phosphoinositide 3‐kinase; PRKDC, protein kinase DNA‐activated catalytic polypeptide; PTEN, phosphatase and tensin homolog; RAF1, Raf‐1 proto‐oncogene; RBFOX2, RNA binding fox‐1 homolog 2; RIOK3, RIO kinase 3; Smad, mothers against decapentaplegic homolog; Snail, zinc finger protein SNAI1; SRY, sex determining region Y; TAF1, TATA‐box binding protein associated factor 1; TAK‐1, transforming growth factor beta‐activated kinase 1; TGF‐β1, transforming growth factor‐beta 1; TGF‐βR1B, transforming growth factor‐beta receptor 1B; TGF‐βR2B, transforming growth factor‐beta receptor 2B; Twist, basic helix‐loop‐helix transcription factor; VEGF, vascular endothelial growth factor; vimentin, intermediate filament protein; Zeb, zinc finger E‐box‐binding homeobox.; α‐SMA, alpha‐smooth muscle actin.

#### 3.1.6 Breast Cancer

Resveratrol, a natural antioxidant compound found in grapes, berries, and peanuts, has shown promising potential in overcoming tamoxifen resistance in hormone‐dependent breast cancer; research indicates that resveratrol works synergistically with tamoxifen, inducing apoptosis in MCF‐7/TR cells. Its chemosensitizing effects are linked to its regulation of the Smad pathway and endogenous TGF‐β production. Specifically, at a concentration of 50 µM, resveratrol reduced TGF‐β production and Smad pathway activity, reversing EMT in MCF‐7/TR cells. This reversal, marked by the return of epithelial markers and a decrease in mesenchymal markers, helped restore the cells' sensitivity to tamoxifen. Thus, resveratrol appears to be a promising therapeutic agent for overcoming acquired tamoxifen resistance by modulating the TGF‐β/Smad pathway and EMT. [164].

The study highlights the effectiveness of *Withania somnifera* root extract (sWRE) in inhibiting metastasis, potentially through mechanisms involving the disruption of vimentin and anti‐EMT effects. The research shows that orally administered sWRE has significant antimetastatic activity, comparable to pure withaferin A (WA), in mouse models. At specific doses, sWRE significantly reduces the formation of metastatic lung nodules without causing significant toxicity, mirroring the effects of WA. Additionally, sWRE inhibits cell motility and invasion in vitro at concentrations lower than its cytotoxic threshold, indicating a mechanism distinct from its antiproliferative effects. The study suggests that sWRE's inhibition of cell motility may involve the disruption of vimentin, a protein associated with aggressive cancers and metastasis. Furthermore, sWRE shows promise in preventing EMT induction and inhibiting invasion in certain cell models. The correlation between sWRE's efficacy and vimentin expression indicates its potential utility against vimentin‐positive tumors, especially in aggressive and metastatic breast cancers. Future research will focus on understanding the pharmacokinetic and pharmacodynamic properties of sWRE to support its potential clinical use as an antimetastatic agent [165].

This study explores caffeine's ability to normalize active breast stromal fibroblasts, reducing their procarcinogenic and metastatic potential. At a concentration of 200 µM, caffeine upregulates key tumor suppressor genes such as p16, p21, p53, and Cav‐1, which are typically downregulated in CAFs. This normalization decreases the expression and secretion of procarcinogenic factors like SDF‐1, MMP‐2, and TGF‐β from CAF cells. Additionally, caffeine impedes the migratory and invasive abilities of CAFs by inhibiting the Erk1/2 and Akt pathways through the upregulation of their common inhibitor, PTEN. Caffeine also reduces the expression of proangiogenic factors like VEGF‐A and HIF‐1α in active breast stromal fibroblasts, thereby inhibiting angiogenesis. The sustained effects of caffeine on CAFs suggest potential epigenetic alterations, indicating its value in cancer prevention or treatment by targeting the TME. The caffeine concentrations used in this study are achievable through daily coffee consumption, underscoring the potential practical relevance of these findings [166].

This study examines WA's multifaceted mechanisms in inducing apoptosis and inhibiting EMT in breast cancer cells. WA promotes apoptosis by upregulating proapoptotic proteins like Bim and downregulating antiapoptotic proteins such as survivin and Mcl‐1. It induces mitochondrial dysfunction by inhibiting complex III of mitochondrial respiration, leading to ROS generation and Bak activation. Additionally, WA modulates MAPK pathways (JNK, ERK, and p38 MAPK) to trigger apoptosis, partly through the inhibition of ERK and p38 MAPK. WA also reverses EMT features induced by TNF‐α and TGF‐β in noncancerous mammary epithelial cells (MCF‐10A), as shown by reduced vimentin expression. Moreover, WA inhibits cell migration by inducing E‐cadherin, an epithelial marker essential for maintaining epithelial integrity. These findings underscore WA's potential as an anticancer agent by targeting key pathways involved in apoptosis and EMT suppression [167].

Thymoquinone (TQ) acts as an important radio‐sensitizer in breast cancer therapy by reducing radiation‐induced toxicity and invasion while inhibiting metastatic progression driven by TGF‐β signaling [168]. The study shows that radiation increases TGF‐β expression, which enhances the migration and invasion of breast cancer cells via the EMT process. Presensitization with TQ restores the balance of TGF‐β and its downstream molecules, suppressing TGF‐β‐induced EMT by downregulating transcriptional repressors such as Snail, Slug, and Twist, which target E‐cadherin transcription. Additionally, TQ reverses the expression of epithelial and mesenchymal markers, restoring E‐cadherin levels and reducing the expression of integrin αV, MMP9, and MMP2 induced by radiation. Furthermore, TQ enhances the apoptotic efficacy in low‐dose irradiated cancer cells by modulating antiapoptotic (Bcl2) and tumor suppressor (p53) proteins, thereby inhibiting metastatic invasion and migration. This study highlights TQ's potential in preventing radiation‐induced EMT and inducing apoptosis in breast cancer cells, supporting its future clinical investigation in chemo‐radiation therapy [152].

Arjunolic acid (AA) shows significant cytotoxic effects against cancer cells, including Ehrlich ascites carcinoma (EAC), leading to reduced cell viability and increased toxicity. This effect is associated with improved animal survival and reduced tumor volume. AA also has antioxidant properties, as it reduces oxidative stress evidenced by lower malondialdehyde (MDA) levels, a marker of oxidative damage. Additionally, AA affects the TGF‐β signaling pathway, which plays an important role in tumor progression and metastasis. While EAC cells exhibit increased TGF‐β1 signaling indicative of advanced cancer, AA treatment inhibits this signaling. AA also restores normal cytokine balance by modulating levels of TNF‐α and IL‐10, which are disrupted in EAC. Furthermore, AA enhances apoptosis by increasing caspase‐3 activity, suggesting its potential therapeutic benefits in cancer treatment through mechanisms such as cytotoxicity, antioxidant effects, cytokine balance modulation, and promotion of apoptosis [169].

Curcumin was studied for its potential to inhibit EMT in MCF‐7 breast cancer cells exposed to endoxifen. Previous research showed that curcumin could reduce vimentin expression, lower TGF‐β1 levels, and increase E‐cadherin expression in cells undergoing EMT. In this study, chronic exposure to endoxifen induced EMT, marked by increased cell viability and changes in EMT markers. Although curcumin treatment initially reduced cell viability when combined with endoxifen, this effect decreased over time. Furthermore, curcumin did not suppress EMT markers such as vimentin or TGF‐β1 mRNA expression, nor did it restore E‐cadherin levels. The study concludes that while curcumin may lower breast cancer cell viability, it is not effective in preventing EMT activation caused by prolonged endoxifen exposure, possibly due to differences in treatment duration and the prooxidant effects of curcumin at higher doses [170].

3,3′‐Diindolylmethane (DIM), derived from indole‐3‐carbinol in cruciferous vegetables, shows potential cancer‐preventive properties. Research has highlighted DIM's ability to inhibit breast cancer development at various stages. Notably, DIM's effect on EMT, an important step in cancer metastasis, was explored. DIM treatment inhibited TNF‐α/TGF‐β‐regulated EMT in human breast cancer cells without affecting cell viability or proliferation. It significantly increased E‐cadherin protein expression in MCF‐7 cells, possibly reversing the loss of epithelial characteristics associated with EMT. Additionally, DIM was found to modulate Smad and non‐Smad signaling pathways induced by TNF‐α/TGF‐β, indicating its role in suppressing EMT markers and potentially inhibiting breast cancer metastasis [171].

**FIGURE 6:**
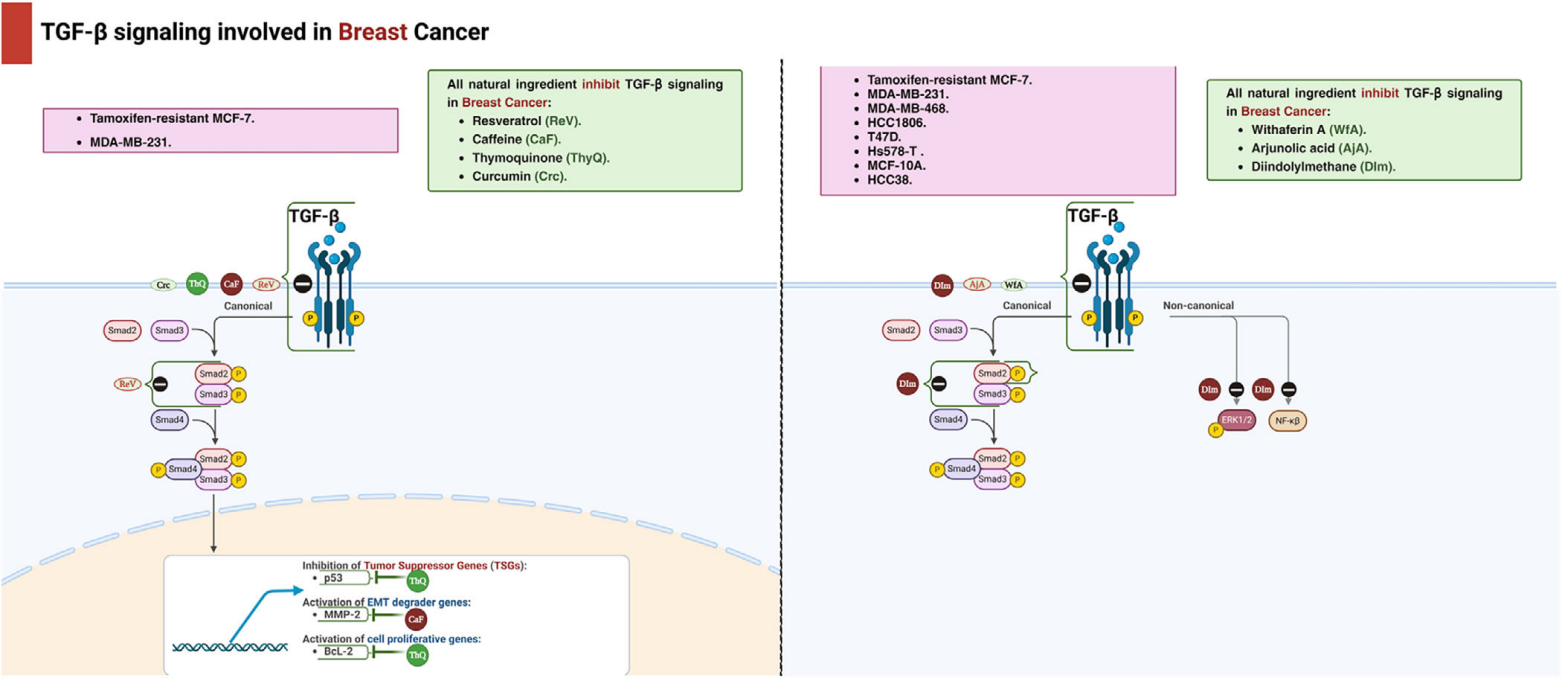
Natural products targeting TGF‐β pathway in treatment of breast cancer. This figure illustrates the involvement of TGF‐β signaling in breast cancer and highlights the effects of various natural ingredients that inhibit TGF‐β signaling. The study includes tamoxifen‐resistant MCF‐7 and MDA‐MB‐231 cell lines in panel A and additional cell lines such as MDA‐MB‐468, HCC1806, T47D, Hs578‐T, MCF‐10A, and HCC38 in panel B. Natural ingredients such as resveratrol (ReV), caffeine (CaF), thymoquinone (ThQ), and curcumin (Crc) in panel A, and withaferin A (WfA), arjunolic acid (AjA), and diindolylmethane (DIm) in panel B inhibit TGF‐β signaling in breast cancer. In panel A, the canonical TGF‐β signaling pathway is shown, where these natural ingredients inhibit the phosphorylation of Smad2 and Smad3, thereby preventing their activation and subsequent nuclear translocation. This inhibition leads to the suppression of tumor suppressor genes (TSGs) like p53, activation of EMT degrader genes like MMP‐2, and activation of cell proliferative genes like Bcl‐2. Panel B continues with the canonical pathway, showing similar inhibition by the listed natural ingredients. Additionally, noncanonical pathways involving ERK1/2 and NF‐κB are inhibited by diindolylmethane (DIm), further preventing TGF‐β induced signaling that promotes cancer progression. AjA, arjunolic acid; Bcl‐2, B‐cell lymphoma 2; CaF, caffeine; Crc, curcumin; DIm, diindolylmethane; E‐cadherin, epithelial cadherin; EMT, epithelial–mesenchymal transition; ERK1/2, extracellular signal‐regulated kinase 1/2; MMP‐2, matrix metallopeptidase 2; NF‐κB, nuclear factor kappa‐light‐chain‐enhancer of activated B cells; p53, tumor protein p53; ReV, resveratrol; Smad, mothers against decapentaplegic homolog; TGF‐β, transforming growth factor‐beta; ThQ, thymoquinone; WfA, withaferin A.

**FIGURE 7:**
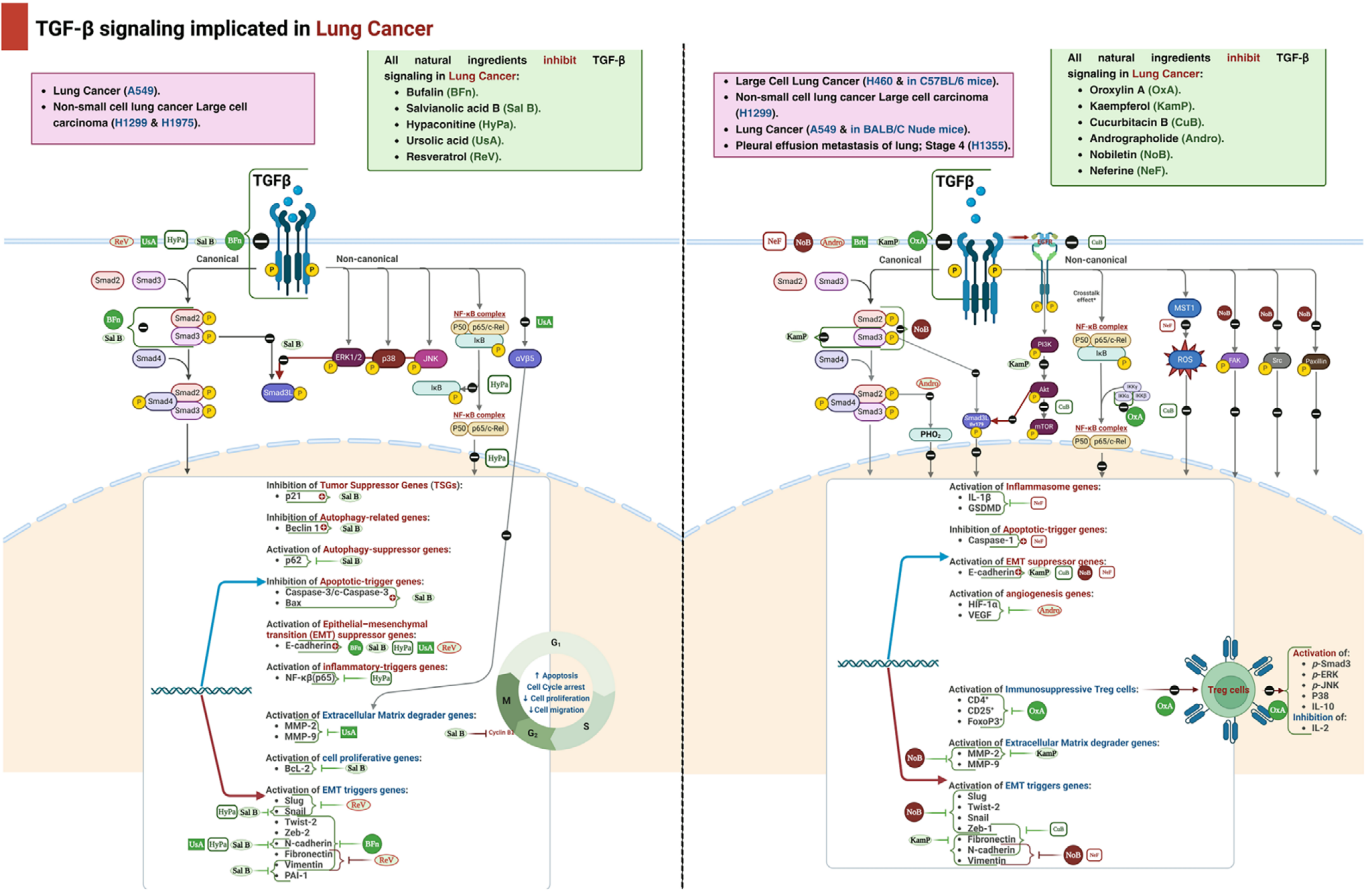
Natural products targeting TGF‐β pathway in treatment of lung cancer. The schematic illustrates the TGF‐β signaling pathway implicated in lung cancer, highlighting the inhibitory effects of various natural products. The natural compounds that inhibit TGF‐β signaling in different lung cancer cell lines (A549, H1299, H1975, H460, H1355, and C57BL/6 mice) are depicted. The canonical and noncanonical pathways are shown, detailing the downstream signaling events and their impact on tumor suppressor genes (TSGs), autophagy‐related genes, apoptotic‐trigger genes, epithelial–mesenchymal transition (EMT) genes, inflammatory‐trigger genes, extracellular matrix degrader genes, cell proliferative genes, and immunosuppressive Treg cells. The natural products bufalin (BFn), salvianolic acid B (Sal B), hypaconitine (HyPa), ursolic acid (UsA), resveratrol (ReV), oroxylin A (OXA), kaempferol (KamP), cucurbitacin B (CuB), andrographolide (Andro), nobiletin (NoB), and neferine (NeF) inhibit various targets within these pathways. A549, human lung carcinoma cell line; Akt, protein kinase B; Andro, andrographolide; BFn, bufalin; Bax, Bcl‐2‐associated X protein; Beclin1, autophagy protein beclin‐1; Bcl‐2, B‐cell lymphoma 2; caspase‐1, cysteine‐aspartic acid protease 1; caspase‐3, cysteine‐aspartic acid protease 3; CD25, interleukin‐2 receptor alpha chain; CD4, cluster of differentiation 4; CuB, cucurbitacin B; E‐cadherin, epithelial cadherin; EMT, epithelial–mesenchymal transition; ERK1/2, extracellular signal‐regulated kinase 1/2; FAK, focal adhesion kinase; FoxP3, Forkhead box P3; GSDMD, gasdermin D; H1355, human lung cancer cell line; H1975, human non‐small cell lung cancer cell line; H1299, human non‐small cell lung cancer cell line; HyPa, hypaconitine; IκB, inhibitor of nuclear factor kappa‐B; IL‐1β, interleukin‐1 beta; IL‐2, interleukin‐2; IL‐10, interleukin‐10; JNK, c‐Jun N‐terminal kinase; KamP, kaempferol; MAPK, mitogen‐activated protein kinase; MMP‐2, matrix metalloproteinase‐2; MMP‐9, matrix metalloproteinase‐9; MST1, macrophage stimulating 1; NeF, neferine; NF‐κB, nuclear factor kappa‐light‐chain‐enhancer of activated B cells; NoB, nobiletin; P38, mitogen‐activated protein kinase P38; PAI‐1, plasminogen activator inhibitor‐1; PHO2, prolyl hydroxylase 2; PI3K, phosphoinositide 3‐kinase; P‐Smad3, phosphorylated Smad3; ReV, resveratrol; ROS, reactive oxygen species; Sal B, salvianolic acid B; Smad2, mothers against decapentaplegic homolog 2; Smad3, mothers against decapentaplegic homolog 3; Smad4, mothers against decapentaplegic homolog 4; Snail, zinc finger protein SNAI1; TGF‐β, transforming growth factor beta; Treg cells, regulatory T cells; Twist2, twist‐related protein 2; UsA, ursolic acid; VEGF, vascular endothelial growth factor; Zeb2, zinc finger E‐box‐binding homeobox 2.

**FIGURE 8:**
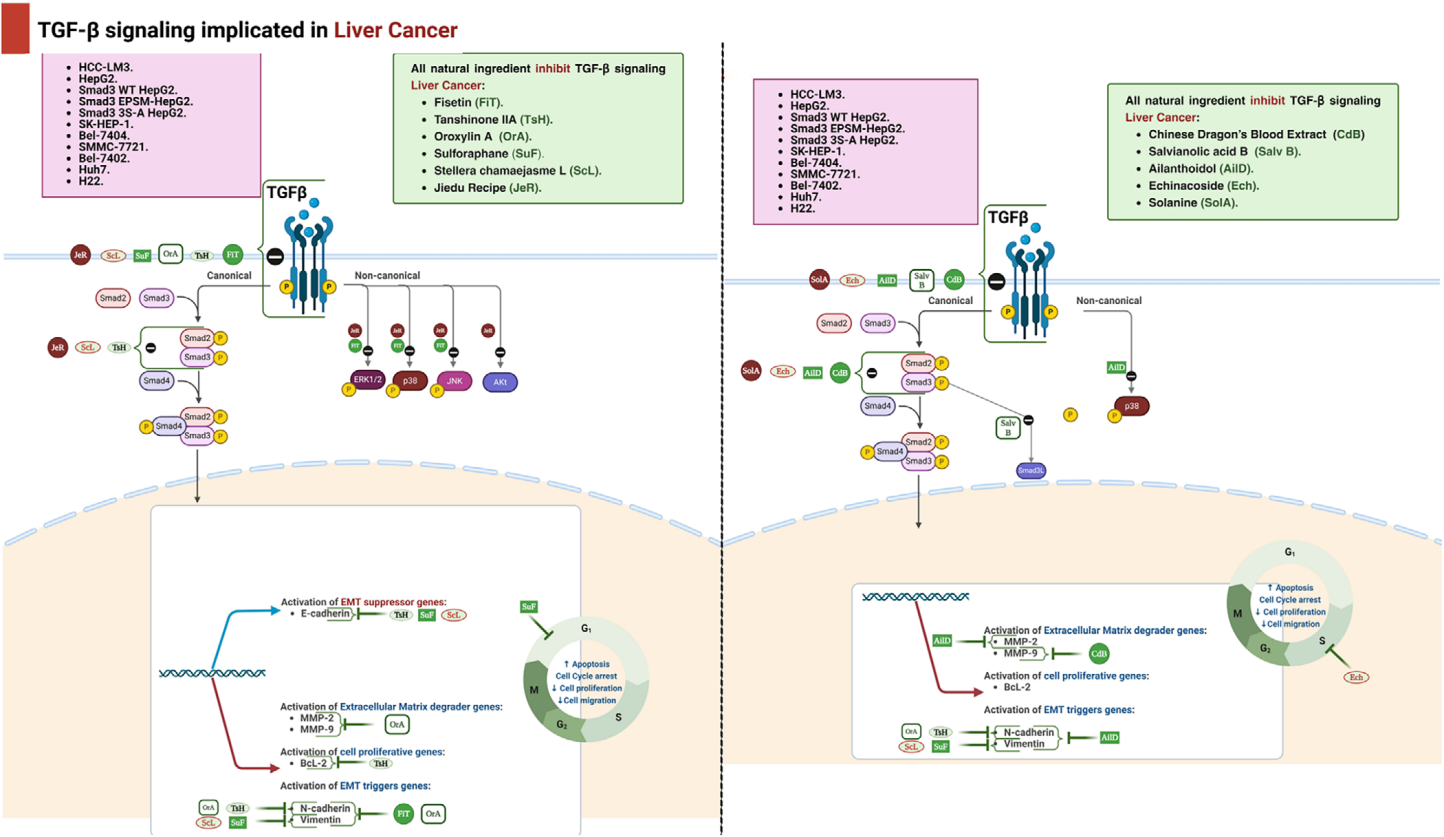
Natural products targeting TGF‐β pathway in treatment of liver cancer. The schematic illustrates the TGF‐β signaling pathway implicated in liver cancer, emphasizing the inhibitory effects of various natural products. The natural compounds that inhibit TGF‐β signaling in different liver cancer cell lines (HCC‐LM3, HepG2, SK‐HEP‐1, SMMC‐7721, Bel‐7402, Huh7, H22) are depicted. The canonical and noncanonical pathways are shown, detailing the downstream signaling events and their impact on epithelial–mesenchymal transition (EMT) suppressor genes, extracellular matrix degrader genes, cell proliferative genes, and apoptotic‐trigger genes. Natural products such as fisetin (FiT), tanshinone IIA (TsH), oroxylin A (OrA), sulforaphane (SuF), Stellera chamaejasme L (ScL), Jiedu recipe (JeR), Chinese dragon's blood extract (CdB), salvanolic acid B (Salv B), ailanthoidol (AllD), echinacoside (Ech), and solanine (SolA) inhibit various targets within these pathways. AllD, ailanthoidol; Akt, akinase; Bel‐7402, human liver cancer cell line; Bel‐7404, human liver cancer cell line; Bcl‐2, B‐cell lymphoma 2; CdB, Chinese Dragon's blood extract; E‐cadherin, epithelial cadherin; Ech, echinacoside; EMT, epithelial–mesenchymal transition; ERK1/2, extracellular signal‐regulated kinase 1/2; FiT, fisetin; H22, human liver cancer cell line; HCC‐LM3, human liver cancer cell line; HepG2, human liver cancer cell line; JNK, c‐Jun N‐terminal kinase; JeR, Jiedu recipe; MAPK, mitogen‐activated protein kinase; MMP‐2, matrix metalloproteinase‐2; MMP‐9, matrix metalloproteinase‐9; N‐cadherin, neural cadherin; p38, mitogen‐activated protein kinase p38; P‐Smad3, phosphorylated Smad3; ScL, Stellera chamaejasme L; SK‐HEP‐1, human liver cancer cell line; Smad2, mothers against decapentaplegic homolog 2; Smad3, mothers against decapentaplegic homolog 3; Smad4, mothers against decapentaplegic homolog 4; Smad3 EPSM, mutant Smad3 with phosphorylation site mutation in the linker region; Smad3 3S‐A, mutant Smad3 with phosphorylation site mutation in the C terminus; SMMC‐7721, human liver cancer cell line; SolA, solanine; SuF, sulforaphane; TGF‐β, transforming growth factor beta; TsH, tanshinone IIA.

Tables 6, 7, 8 and Figures 6, 7, 8 summarize the effects of various natural products on TGF‐β signaling in breast cancer, demonstrating their potential as therapeutic agents in modulating cancer progression and metastasis.

**TABLE 6:** Natural products targeting TGF‐β pathway in breast cancer.

Compound (chemical class)	Cancer cell lines	Tested concentrations	Mechanism/signaling pathways	Results (effect on cancer cells)	References
Resveratrol (polyphenol)	MCF‐7/TR	50 µM	TGF‐β/Smad pathway	↓TGF‐β production, reversed EMT, restored tamoxifen sensitivity	[164]
*Withania somnifera* root extract (steroidal lactones)	Metastatic lung nodules	Specific doses not provided	Disruption of vimentin, anti‐EMT effects	↓Formation of metastatic lung nodules, ↓Cell motility ↓Invasion	[165]
Caffeine (xanthine alkaloid)	Cancer‐associated fibroblasts (CAFs)	200 µM	Erk1/2 and Akt pathways	↑Tumor suppressor genes, ↓Procarcinogenic factors, ↓Migratory and invasive abilities	[166]
Withaferin A (steroidal lactone)	MCF‐10A	Not specified	MAPK pathways (JNK, ERK, and p38 MAPK)	↑Apoptosis, reversed EMT features, ↓Cell migration	[167]
Thymoquinone (quinone derivative)	Not specified	Not specified	TGF‐β signaling	↓TGF‐β‐induced EMT, ↓Snail, ↓Slug, ↓Twist, ↑apoptotic efficacy	[152]
Arjunolic acid (triterpenoid)	Ehrlich ascites carcinoma (EAC)	Not specified	TGF‐β signaling	↓TGF‐β1 signaling, ↑Apoptosis, restored normal cytokine balance	[169]
Curcumin (polyphenol)	MCF‐7	Not specified	TGF‐β/EMT markers	↓Cell viability, not effective in preventing EMT caused by prolonged endoxifen exposure	[170]
3,3′‐diindolylmethane (DIM) (indole derivative)	MCF‐7	Not specified	Smad and non‐Smad signaling pathways	↑E‐cadherin expression, ↓TNF‐α/TGF‐β‐regulated EMT	[171]

Abbreviations: AjA, arjunolic acid; CaF, caffeine; CAFs, cancer‐associated fibroblasts; Crc, curcumin; DIM, 3,3′‐diindolylmethane; EAC, Ehrlich ascites carcinoma; EMT, epithelial–mesenchymal transition; MAPK, mitogen‐activated protein kinases; ReV, resveratrol; sWRE, *Withania somnifera* root extract; TGF‐β, transforming growth factor‐beta); ThQ, thymoquinone); WfA, withaferin A; ↑, increase; ↓, decrease.

**TABLE 7:** In vivo studies on natural products targeting TGF‐β pathway in cancer models.

Compound (chemical class)	Animal model	Dosage	Mechanisms	Results	References
Ethanolic olive leaf extract (polyphenol‐rich extract)	Male albino leukemic mice (ENU‐induced leukemia)	200 mg/kg, orally, 4 weeks	Modulation of immune response, ↓GF‐β levels	↑IFN‐γ ↑Granzyme ↑Perforin ↓TGF‐β l	[173]
Halofuginone (alkaloid)	Transgenic APL mice	150 µg/kg/day, 21 days	Antiangiogenic, antiproliferative, proapoptotic actions	↓VEGF, HGF, HIF‐1α, ANGPT‐1 and ‐2; ↑TIMP2, CXCL10; ↓Tumor burden, ↓c‐myc, ↑p15, ↑p21, ↑TGF‐β1, ↑p‐Smad3, ↑TGF‐βRII	[95, 96]
Oroxylin A (flavonoid)	C57BL/6 mice with Lewis and H460 tumor cells	60 mg/kg, intragastrically	Inhibition of Treg cells, ↓Foxp3, ↓CD4 ↓mRNA	↓Treg cells, ↓tumor growth	[101]
Cucurbitacin B (triterpenoid)	Mice with lung cancer metastasis	0.25 mg/kg and 0.5 mg/kg, intratracheally	Inhibition of metastasis	↓Lung cancer dissemination	[109]
Nobiletin (flavonoid)	Nude mice with A549 cells	20 and 40 mg/kg, orally	Modulation of EMT markers ↑E‐cadherin ↓N‐cadherin	↓Metastasis	[113]
Neferine (alkaloid)	BALB/c nude mice with A549 cells	10, 20, 30 mg/kg/day, intraperitoneally	Control of MST1 expression, TGF‐β modulation ↑ROS,	Pyroptosis, ↓cell proliferation, metastasis, and EMT	[114]
Digitoxin (cardiac glycoside)	Mice with metastatic prostate cancer	—	↓NF‐κB ↓EMT signaling	↓Metastasis, no primary tumor recurrence	[157]
Osthole (coumarin derivative)	Mice with prostate gland malignancies	30 and 100 mg/kg, in vivo	Inhibition of EMT markers	↓Growth and metastasis	[128]
Fisetin (flavonoid)	BALB/c nude mice with liver cancer HCC‐LM3 cells	20, 40, 80 mg/kg, intraperitoneally, 7 weeks	Reduction of TGF‐β1 and Ki‐67	↓Tumor weight, prolonged survival	[26]
CDBEE (polyphenol‐rich extract)	Nude mice with HepG2 cells	250 mg/kg, intragastrically	Inhibition of Smad3 expression	↓Tumorigenicity, ↓ Ki67, MMP9, no weight loss or organ damage	[120]
Tanshinone IIA (diterpenoid quinone)	BALB/c nude mice with Bel‐7404 cells	10 mg/kg/day, intraperitoneally	Regulation of Smad7/YAP interaction, TGF‐β pathway	↓Tumor volume, ↓Ki67, ↓Bcl2 ↓YAP ↑SMAD7	[121]
Oroxylin A (flavonoid)	BALB/c nude mice with SMMC‐7721 cells	—	NAG‐1 mediated inhibition of TGF‐β‐induced Smad signaling	↓Lung mass colonization, prolonged survival, ↓E‐cadherin, ↑Vimentin ↑Twist1	[126]
Sulforaphane (isothiocyanate)	BALB/c athymic nude mice with HepG2 cells	50 mg/kg, intraperitoneally, every 2 days	Inhibition of tumor growth	↓Tumor growth, ↑ tumor volume in control mice	[128]
Salvianolic acid B (polyphenol)	C57BL/6J mice (WT and HT) with DEN administration	15 mg/kg, orally, 23 weeks	Targeting C‐terminal of Smad3	↓Tumor growth, ↑pSmad3C,↑ p21, ↑PAI‐1, ↓pSmad3L, ↑α‐SMA	[130]
Solanine (steroidal alkaloid)	Female nude mice with H22 cells	37.5 mg/kg, intraperitoneally	Reduction of Treg cells, TGF‐β/Smad signaling	↓Tumor growth, metastasis, proliferation, ↓Treg cells, TGF‐β, IL‐10, Foxp3, p‐Smad2, p‐Smad3	[139]

Abbreviations: APL, acute promyelocytic leukemia; CDBEE, Chinese Dragon's blood EtOAc extract; CDKIs, cyclin‐dependent kinase inhibitors; CuB, cucurbitacin B; DEN, diethyl nitrosamine; EMT, epithelial–mesenchymal transition; EOLE, ethanolic olive leaf extract; HGF, hepatic growth factor; HT, heterozygous; MMP9, matrix metalloproteinase 9; MST1, macrophage‐stimulating 1; NAG‐1, nonsteroidal anti‐inflammatory drug‐activated gene‐1; NF‐κB, nuclear factor kappa‐light‐chain‐enhancer of activated B cells; NOD/SCID, nonobese diabetic severe combined immune deficiency; OXXXA, oroxylin A; Sal B, salvianolic acid B; SFN, sulforaphane; Smad, mothers against decapentaplegic; Smad3, small mothers against decapentaplegic 3; Smad7, small mothers against decapentaplegic 7; TGF‐β, transforming growth factor beta; TIMP2, tissue inhibitor of metalloproteinases 2; Treg cells, regulatory T cells; VEGF, vascular endothelial growth factor; WT, wild type.

**TABLE 8:** Clinical trials of natural compounds targeting the TGF‐β pathway in cancer treatment.

Cancer type	Treatment	Patient group/dosage	Duration	Mechanism/signaling pathways	Results	References
Acute myeloid leukemia with myelodysplasia‐related changes (AML‐MRC)	Green tea extract (GT)	Older individuals ineligible for aggressive chemotherapy or bone marrow transplants; 1000 mg/day	At least 6 months or until disease progression	↑Perforin+/granzyme B+ NK cells; ↑CD8+ T cells; ↓TGF‐β; ↓Treg cells; ↓ROS	↑Immune profile, ↑Survival rate, ↓Disease progression	[179]
Chronic lymphocytic leukemia (CLL)	Green tea extract (GT)	Stage 0 CLL patients and healthy individuals; 4 capsules/day (1 month), then 6 capsules/day (5 months)	6 months	↓Lymphocytosis, ↓Treg cells, ↓TGF‐β, ↓IL‐10	↓Lymphocytosis, ↓Treg cells, ↓disease progression	[180]
Breast cancer	Curcumin	Patients receiving radiotherapy; 6 g daily	14–21 days presurgery	↓Radiation‐induced inflammatory markers; potential modulation of TGF‐β signaling	↓Inflammation, potential enhancement of therapeutic response, ↓radiotherapy adverse effects	(NCT01740323) [181]
Lung cancer (high‐risk former smokers)	Sulforaphane	Former smokers at high risk for lung cancer; 120 micromoles daily	12 months	↓Cell proliferation markers (e.g., Ki‐67), ↑Apoptosis (caspase‐3 activation) through TGF‐β modulation	↓Bronchial dysplasia progression; potential protective effect	[182]

Abbreviations: AML‐MRC, acute myeloid leukemia with myelodysplasia‐related changes; CLL, chronic lymphocytic leukemia; GT, green tea; IL‐10, interleukin 10; NK cells, natural killer cells; ROS, reactive oxygen species; TGF‐β, transforming growth factor beta; Treg cells, regulatory T cells. ↑, increase; ↓, decrease.

### 3.2 In Vivo Studies

Ethanolic olive leaf extract (EOLE) is a traditional herbal remedy that has cytotoxic, anti‐inflammatory, and immune‐modulatory properties [172]. This study assessed the anticancer and immune‐modulatory effects by utilizing a mouse model of leukemia induced by ethyl‐nitroso‐urea. In this experiment, male albino leukemic mice were administered 200 mg/kg of EOLE for a duration of 4 weeks. Leukemic mice that received EOLE treatment exhibited enhanced overall immune response, as seen by elevated levels of interferon‐γ (IFN‐γ), granzyme, and perforin, along with reduced levels of serum TGF‐β [173]. In many solid tumor types, the alkaloid HF has demonstrated antiangiogenic, antiproliferative, and proapoptotic actions [174]. It has been observed that increased angiogenesis and VEGF are associated with the progression of AML. The researchers utilized a mouse model of acute promyelocytic leukemia (APL) to examine the in vivo impact of HF. Leukemic cells from transgenic mice with human chorionic gonadotropin–promyelocytic leukemia/retinoic acid receptor alpha (HCG‐PML/RARA) were transplanted into mice with nonobese diabetic severe combined immune deficiency. The mice were then treated with HF at a dosage of 150 µg/kg/day for a period of 21 days. The results revealed that HF decrease the number of VEGF+ cells and the expression of the VEGF gene. Additionally, HF treatment also lowered the levels of several proangiogenic factors, including HGF, HIF‐1α, ANGPT‐1, and ‐2. Conversely, HF treatment led to an increase in the expression of the antiangiogenic factors TIMP2 and CXCL10. Consequently, HF treatment effectively reduced the tumor burden in APL mice. Moreover, the study showed that in mice with APL, the regulation of TGF‐β signaling by HF is essential for preventing the formation angiogenesis [95]. Another mouse model, which has been transplanted with APL, likewise utilizes HF. HF was intraperitoneally given to APL transplant mice for a duration of 21 days, with a dosage of 150 µg/kg/day. The data indicate that HF therapy leads to cell cycle arrest and death via activating the TGF‐β signaling pathway, which is mediated by elevated levels of TGF‐β1, phosphorylation of Smad 3, and expression of TGF‐βRII. The findings indicated a decrease in the expression of c‐myc and an increase in the expression of the CDKIs p15 and p21. These changes may have had a role in inhibiting the growth by TGF‐β and inducing apoptosis. Finally, these findings are consistent with the activation of the TGF‐β pathway generated by HF [96].

The impact of OA on tumor development and the number of Treg cells was examined through the intragastric administration of a dosage of 60 mg/kg every other day. This administration continued until palpable tumors occurred or for a duration of 2 weeks following the injection of 8 × 105 Lewis and 3 × 106 H460 tumor cells subcutaneously in the right suprascapular area of C57BL/6 mice. The administration of OA resulted in a decrease in the mRNA expression of Foxp3 and CD4 in xenografts, leading to a considerable reduction in the fraction of Treg cells [101].

The in vivo investigation demonstrated that CuB effectively suppressed lung cancer metastasis when administered intratracheally (0.25 and 0.5 mg/kg) for 14 days, after 4 days of intravenous injection of B16‐F10‐Luc cells (5 × 10^5^/mice) into the mice [109]. An in vivo model was created by intravenously injecting A549 cells into nude mice through the tail vein. Once daily, nobiletin was supplied orally at doses of 20 and 40 mg/kg. Nobiletin significantly suppressed metastasis and promoted the expression of E‐cadherin protein, while inhibiting N‐cadherin [113]. BALB/c nude mice were subcutaneously injected with A549 cells in either the right or left axilla of each mouse. Neferine was given intraperitoneally at a dosage of 10, 20, or 30 mg/kg/day. Neferine controls the expression of MST1 by reducing the levels of TGF‐β, which leads to the generation of ROS and affects pyroptosis, cell proliferation, metastasis, and EMT in NSCLC [114].

Regarding in vivo examination, Pollard et al. [157] suggested that digitoxin might stop metastasis seeding on day 10 and allow only established metastases to survive and proliferate. Digitoxin also slowed the growth of implanted metastases, whether seeded from the primary tumor or injected intravenously. The postmortem examination on day 80 of digitoxin‐treated survivors indicated no primary tumor recurrence following excision [157]. At 30 and 100 mg/kg dosages, Wen et al. [158] found that the growth and metastasis of transplanted prostate gland malignancies were significantly inhibited in vivo by osthole.

The beneficial impact of Fis on liver cancer cell growth was demonstrated by utilizing a hepatoma model in which 6‐week‐old male, athymic BALB/C nude mice were implanted with liver cancer HCC‐LM3 cells (3 × 10^6^ cells) orthotopically. Then, tumor‐bearing mice received Fis (20, 40, and 80 mg/kg) daily intraperitoneally for 7 weeks. Fis was found to reduce the weight of tumors and prolong the survival time of mice. In addition, the levels of TGF‐β1 and Ki‐67 were considerably lower in tumor tissue specimens obtained from mice treated with Fis [117].

Chen et al. [120] demonstrated the effect of CDBEE on hepatocellular carcinoma. HepG2 cells were subcutaneously injected into the right posterior back region of male BALB/c nude mice. A daily intragastric injection of 250 mg/kg CDBEE was reported to reduce the tumorigenicity of human hepatomas HepG2 cells in nude mic. Moreover, the nude mice did not significantly lose weight. Furthermore, the liver, spleen, kidney, lung, or heart of nude mice showed no signs of evident CDBEE‐related damage. Importantly, anti‐HCC effect of CDBEE was mostly attributed to inhibition of Smad3 expression, which is considered an important element of the TGF‐β/Smad pathway. Furthermore, a decrease in Ki67 and MMP9 levels in nude mice supported the suppressive action of CDBEE on proliferation and metastasis.

In vivo study demonstrated the suppressing effect of TanIIA on liver cancer cell growth by using Balb/c nude mice that were injected with Bel‐7404 cells. Then, after 14 days, mice were given injections of either diluted DMSO or TanIIA (10 mg/kg/d) [121]. TanIIA was proven to contribute to regulation of the Smad 7/yes‐associated protein (YAP) interaction. As Smad 7 is considered the negative regulator of the TGF‐β signaling pathway, that ends up blocking Smad2/3 phosphorylation. YAP is an oncogene member of the Hippo pathway that contributes to liver tumorigenesis. The antitumor effects TanIIA showed were demonstrated by a decline in tumor volume and protein expression of Ki6, Bcl2, and YAP. Meanwhile, SMAD7 expression was upregulated in TanIIA‐treated groups as compared with the DMSO‐treated group.

A lung metastasis model included BALB/c nude mice were intravenously infected with shCon/shNAG‐1SMMC‐7721 cells (knock‐down of NAG‐1) demonstrated the antimetastasis potential of OA [126]. The abovementioned study highlighted the important role that NAG‐1 plays in blocking the TGF‐β‐induced Smad signaling pathway, which has been linked to the antimetastatic properties that OA has in HCC. Importantly, it was shown that the repressive effect of OA on metastasis will cease to exist upon NAG‐1 knockdown. This was confirmed by bodyweight analysis results and survival curves, which revealed that upon OA treatment, there was a reduction in lung mass colonization, a rescue in the loss of body weight, and a prolongation in survival time in the control group, whereas OA failed to do so in sh*NAG‐1* cell implanted mice. Furthermore, a lower expression of E‐cadherin and a higher expression of vimentin and Twist1 were also noticed in the sh*NAG‐1* group, as compared with the control group. All these findings suggest an indispensable role for NAG‐1 in OA's inhibition of HCC metastasis.

Additionally, another study proposed the suppressing effect of SFN on the growth of HCC cells by injecting Balb/c athymic nude mice with HepG2 cells subcutaneously in the right flank. SFN (50 mg/kg) was administered intraperitoneally to nude mice every 2 days for 13 days. The findings of this study revealed that HepG2 cell‐derived xenograft tumors grew slowly in the SFN‐treated mice compared with the progressive growth that was induced in the control mice, represented by a larger tumor volume [128].

In vivo study illustrated the beneficial impacts of Sal B toward hepatocarcinogenesis specifically by targeting a phosphorylation site on the C‐terminal of Smad3. The study utilized WT C57BL/6J mice with the genotype pSmad3C+/+ and Smad3 C‐terminal phosphorylation site‐heterozygous mutant (HT) mice with the genotype pSmad3C+/' to explore the impact of Sal B on hepatocarcinogenesis following DEN administration. The study discovered that administering Sal B (15 mg/kg) orally for 23 weeks effectively reduced the rate of growth of liver cancer induced by DEN, as evidenced by a marked rise in expression of pSmad3C, p21, and PAI‐1, and a decrease in that of pSmad3L, and protein levels in the liver tissues of mice, particularly in those of WT mice. Additionally, Sal B reduced α‐SMA and glutathione S‐transferase P was also reported [130].

Treg cells were reported to be involved in the secretion of TGF‐β, resulting in inhibition of the body's antitumor immunity. Importantly, Gao et al. [139] proposed the impact of solanine on CD4+CD25+ Treg cells and linked these beneficial effects to the antitumor effect that solanine exerted in HCC. Female nude mice with SPF grade were subcutaneously injected with H22 cells. It was found that intraperitoneal injection of solanine at a dose of 37.5 mg/kg significantly inhibited the growth, metastasis, and proliferation of tumor cells. Besides, solanine reduced the number of Treg cells, TGF‐β, and IL‐10 and, importantly, suppressed the expression of Foxp3, p‐Smad2, and p‐Smad3. Overall, solanine has been found to decrease the number of local CD4+CD25+Foxp3+ Treg cells in mice and boost the body's immune response against tumors by blocking the TGF‐β/Smad signaling pathway.

The preclinical studies covered in this section demonstrate that natural compounds can target the TGF‐β pathway in both in vitro and in vivo models, providing a strong foundation for developing these agents as complementary cancer therapies. These findings support continued investigation into their translational potential, particularly for applications in cancer types where TGF‐β signaling is a key driver of disease progression.

## 4 Clinical Studies of Natural Compounds Targeting the TGF‐β Pathway in Cancer

Polyphenols are naturally occurring compounds found in a variety of foods such as red fruits, vegetables, cereals, oils, and particularly in tea, which is the second most consumed beverage in the world after water [1, 175, 176]. These compounds show significant potential for beneficial biological effects in combating malignancies, diabetes, and cardiovascular disease [177]. GT extract, which is high in polyphenols, has been found to alter the process of cell growth, cell death, and other characteristics associated with the development of cancer [178].

An evaluation was conducted to determine the effect of using GT extract on the treatment of older individuals with AML and have myelodysplasia‐related changes (AML‐MRC) who are ineligible for aggressive chemotherapy or bone marrow transplants. During the phase II study, 10 eligible patients were administered oral doses of GT extract (1000 mg/day), either alone or in combination with low‐dose cytarabine chemotherapy, for a minimum of 6 months or until disease progression. Enhancements in the patients’ immune profiles and an increase in the survival rate were seen. After 30 days, an enhanced cytotoxic and active phenotype was observed, characterized by an increase in perforin+/granzyme B+ NK cells and total and naïve/effector cluster differentiation 8+ (CD8+) T cells. In addition, there was a decrease in the immunosuppressive characteristics: GT reduced the levels of TGF‐β, the frequencies of Treg cells, and ROS. GT has been found to inhibit the progression of the disease by bolstering the immune system's surveillance of cancer cells [179].

A separate phase II clinical investigation evaluated the efficacy of GT extract as a therapy. A total of 12 patients diagnosed with stage 0 chronic lymphocytic leukemia (CLL) and 12 healthy individuals were given GT extract orally. In the first month, they took 4 capsules per day, and for the following 5 months, they took six capsules each day. The study's findings indicate that 80% of the evaluable patients exhibited decreased levels of lymphocytosis and Treg cells in their general circulation. It is noteworthy that the levels of TGF‐β and IL‐10 in the bloodstream dropped in both the patients and the control group over the duration of GT consumption. Our observations indicate that GT has the potential to alter the levels of circulating Treg cells in CLL patients with an early stage of the disease via inhibiting TGF‐β signaling. Ultimately, through boosting the immune system's ability to detect and monitor cancer cells, GT can result in the control of an excessive increase in lymphocytes and the inhibition of the advancement of the disease [180].

Another phase II clinical trial (NCT01740323) assessed the effect of curcumin as an adjunct therapy in breast cancer patients receiving radiotherapy. Patients were administered 6 g of curcumin daily, and the study primarily evaluated curcumin's impact on radiation‐induced inflammatory markers, including TGF‐β signaling elements. Results suggested that curcumin reduced inflammation and potentially modulated TGF‐β activity, thus enhancing the therapeutic response and reducing adverse effects of radiotherapy in breast cancer patients (NCT01740323) [181].

A clinical trial (NCT03232138) studied SFN in former smokers at high risk for lung cancer, administering 120 micromoles of SFN daily for 12 months. This double‐blind, placebo‐controlled study evaluated the impact on bronchial dysplasia, a premalignant change in the bronchial epithelium. The trial found that SFN significantly modulated markers related to cell proliferation (e.g., Ki‐67) and increased apoptosis (caspase‐3 activation) in bronchial biopsies, indicating a possible protective effect through TGF‐β pathway modulation (NCT03232138) [182]

These trials provide a foundation for using natural compounds as adjunct therapies in cancer treatment, particularly through TGF‐β pathway modulation, which could enhance therapeutic efficacy while minimizing side effects.

## 5 Therapeutic Limitations, Challenges, and Clinical Gaps

Natural products targeting the TGF‐β pathway have demonstrated promising anticancer properties; however, several limitations hinder their clinical application. One significant limitation is the variability in bioavailability and pharmacokinetics of these compounds. Many natural products exhibit poor solubility, stability, and absorption, leading to insufficient therapeutic concentrations at the target sites. For example, compounds such as ginsenosides and EGCG show significant anticancer activities in vitro but face challenges in achieving effective systemic concentrations in vivo. Another limitation is the potential for off‐target effects and toxicity. While natural products are often perceived as safe, their complex chemical structures can interact with multiple cellular pathways, leading to unintended effects. The diverse biological activities of compounds like HF and bufalin require careful dosage optimization to minimize adverse reactions. Additionally, the lack of standardized extracts and the presence of multiple active ingredients in natural products complicate the evaluation of their safety profiles.

A major challenge in the therapeutic application of natural products is the inconsistency in clinical efficacy due to variability in source materials and extraction methods; the quality and concentration of bioactive compounds can differ significantly between batches, leading to inconsistent therapeutic outcomes [183]. This variability poses a challenge for clinical trials, where reproducibility and standardization are critical. Moreover, the complexity of the TGF‐β signaling pathway itself presents challenges. The dual role of TGF‐β in cancer, acting as both a tumor suppressor in early stages and a promoter in advanced stages, complicates the therapeutic targeting. Effective modulation of this pathway requires precise control over the context‐specific actions of TGF‐β, which is difficult to achieve with broad‐acting natural compounds. Despite extensive preclinical studies, there is a significant gap in clinical trials evaluating the efficacy of natural products targeting the TGF‐β pathway. Most studies remain at the in vitro or animal model stages, and few have progressed to human trials. This gap highlights the need for well‐designed clinical trials to establish the safety, efficacy, and dosage parameters of these compounds in cancer patients. Furthermore, there is a lack of comprehensive understanding of the mechanisms of action of many natural products at the molecular level. Detailed mechanistic studies are essential to elucidate how these compounds interact with the TGF‐β pathway and other signaling networks within the TME. Bridging this gap requires collaborative efforts between chemists, biologists, and clinical researchers to develop targeted therapies that can be effectively translated into clinical practice.

## 6 Conclusion and Future Perspectives

Natural products targeting the TGF‐β signaling pathway hold significant promise in cancer therapy, offering potential advantages in terms of efficacy and reduced side effects compared with conventional treatments. The dual role of TGF‐β in cancer progression—as both a tumor suppressor and promoter depending on the stage and context—presents a unique therapeutic challenge and opportunity. This review has highlighted the diverse range of natural compounds that modulate the TGF‐β pathway across various cancer types, with compounds such as ginsenosides, HF, EGCG, and bufalin demonstrating notable anticancer activities by influencing cell proliferation, apoptosis, angiogenesis, and immune responses. These natural products have shown promising results in preclinical studies, effectively inhibiting tumor growth, metastasis, and immune evasion by targeting key components of the TGF‐β pathway. Despite these promising findings, translating the therapeutic potential of natural products into clinical practice remains challenging. Issues such as variability in bioavailability, inconsistent pharmacokinetics, and potential off‐target effects complicate their development as reliable cancer therapies. Moreover, the complex nature of the TGF‐β pathway, with its context‐dependent effects in cancer, further complicates precise therapeutic targeting. Effective modulation of this pathway requires nuanced control over TGF‐β’s actions in specific cellular environments, which is often difficult to achieve with broad‐acting natural compounds. This underscores the need for advanced formulations and delivery systems that can improve bioavailability, stability, and specificity of these compounds in clinical settings. Future research should prioritize overcoming these limitations through innovative approaches. Developing advanced delivery systems, such as nanoparticles and targeted drug delivery carriers, could significantly enhance the therapeutic potential of natural products by increasing their stability and bioavailability. Additionally, identifying novel natural compounds with specific TGF‐β targeting capabilities will be critical. Employing advanced screening techniques, structural characterization, and in‐depth mechanistic studies will aid in uncovering new natural compounds and in optimizing their activity for precise TGF‐β pathway modulation. Furthermore, combining natural product‐based agents with existing cancer therapies could improve treatment outcomes and address drug resistance. Such combination therapies require a comprehensive understanding of interactions between natural compounds and conventional treatments, highlighting the need for interdisciplinary research to maximize therapeutic synergy. Rigorous clinical trials are essential to validate the therapeutic potential of natural products targeting the TGF‐β pathway. These trials should focus on establishing safety, efficacy, and optimal dosages in cancer patients, supported by mechanistic studies to elucidate how these compounds interact with TGF‐β and other pathways within the TME. Additionally, such trials could explore patient‐specific factors, given that individual responses to natural compounds may vary. By building a strong clinical evidence base, researchers can better define the role of natural products as complementary therapies in cancer care, laying the groundwork for personalized and targeted treatment options. In conclusion, natural products represent a promising avenue for cancer therapy targeting the TGF‐β pathway, with the potential to provide safer, more affordable, and effective complementary treatments. Addressing current therapeutic limitations and clinical gaps through rigorous research and clinical evaluation will be key to integrating these compounds into conventional cancer treatment regimens. Such integration aims to offer new therapeutic options that enhance patient quality of life, improve clinical outcomes, and reduce the overall burden of cancer on healthcare systems.

## Author Contributions

Reham Hassan Mekky, Mohammed E. Abo‐El Fetoh, Safaa A Faheem, Abdullah F. Radwan, Mariam H. Fawzy, Aya M. Mustafa, Mohamed A. Said, Daniela Calina, Javad Sharifi‐Rad, and William C. Cho made a significant contribution to the work reported, whether that is in the conception, study design, execution, acquisition of data, analysis, and interpretation, or all these areas. That is revising or critically reviewing the article; giving final approval of the version to be published; agreeing on the journal to which the article has been submitted; and, confirming to be accountable for all aspects of the work. All authors have read and approved the final manuscript.

## Ethics Statement

The authors have nothing to report.

## Conflicts of Interest

The authors wish to confirm that there are no known conflicts of interest associated with this publication and there has been no significant financial support for this work that could have influenced its outcome.

## Data Availability

The authors have nothing to report.

## References

[1] G. E. Chaudhry , A. Md Akim , Y. Y. Sung , and T. M. T. Sifzizul , “Cancer and Apoptosis: The Apoptotic Activity of Plant and Marine Natural Products and Their Potential as Targeted Cancer Therapeutics,” Frontiers in Pharmacology 13 (2022): 842376. 36034846 10.3389/fphar.2022.842376 PMC9399632

[2] P. Costa , S. L. A. Sales , D. P. Pinheiro , et al., “Epigenetic Reprogramming in Cancer: From Diagnosis to Treatment,” Frontiers in Cell and Developmental Biology 11 (2023): 1116805. 36866275 10.3389/fcell.2023.1116805 PMC9974167

[3] A. R. da Cunha , K. Compton , R. X. Xu , et al., “The Global, Regional, and National Burden of Adult Lip, Oral, and Pharyngeal Cancer in 204 Countries and Territories a Systematic Analysis for the Global Burden of Disease Study 2019,” Jama Oncology 9, no. 10 (2023): 1401–1416. 37676656 10.1001/jamaoncol.2023.2960 PMC10485745

[4] B. Liu , H. Zhou , L. Tan , K. T. H. Siu , X. Y. Guan , “Exploring Treatment Options in Cancer: Tumor Treatment Strategies,” Signal Transduction and Targeted Therapy 9, no. 1 (2024): 175, 10.1038/s41392-024-01856-7. 39013849 PMC11252281

[5] U. Anand , A. Dey , A. K. S. Chandel , et al., “Cancer Chemotherapy and Beyond: Current Status, Drug Candidates, Associated Risks and Progress in Targeted Therapeutics,” Genes & Diseases 10, no. 4 (2023): 1367–1401. 37397557 10.1016/j.gendis.2022.02.007 PMC10310991

[6] M. Ammad , Z. Javed , H. Sadia , et al., “Advancements in Long Non‐coding RNA‐based Therapies for Cancer: Targeting, Delivery, and Clinical Implications,” Medical Oncology 41, no. 11 (2024): 292. 39428417 10.1007/s12032-024-02534-y

[7] D. C. Attri , P. Dhyani , V. L. Trivedi , et al., “Current Evidence on Molecular Mechanisms of Andrographolide in Cancer,” Current Medicinal Chemistry (2024). 10.2174/0109298673295496240530100728 38867528

[8] R. Bala , R. Madaan , S. Chauhan , et al., “Revitalizing allicin for Cancer Therapy: Advances in Formulation Strategies to Enhance Bioavailability, Stability, and Clinical Efficacy,” Naunyn‐Schmiedeberg's Archives of Pharmacology 397, no. 2 (2024): 703–724. 37615709 10.1007/s00210-023-02675-3

[9] E. Batlle and J. Massagué , “Transforming Growth Factor‐β Signaling in Immunity and Cancer,” Immunity 50, no. 4 (2019): 924–940. 30995507 10.1016/j.immuni.2019.03.024 PMC7507121

[10] S. Liu , J. Ren , and P. Ten Dijke , “Targeting TGFβ Signal Transduction for Cancer Therapy,” Signal Transduction and Targeted Therapy 6, no. 1 (2021): 8. 33414388 10.1038/s41392-020-00436-9 PMC7791126

[11] A. B. Baba , B. Rah , G. R. Bhat , et al., “Transforming Growth Factor‐Beta (TGF‐β) Signaling in Cancer‐A Betrayal within,” Frontiers in Pharmacology 13 (2022): 791272. 35295334 10.3389/fphar.2022.791272 PMC8918694

[12] T. Matsuoka and M. Yashiro , “The Role of the Transforming Growth Factor‐β Signaling Pathway in Gastrointestinal Cancers,” Biomolecules 13, no. 10 (2023): 1551. 37892233 10.3390/biom13101551 PMC10605301

[13] N. A. Kuburich , T. Sabapathy , B. R. Demestichas , J. J. Maddela , den P. Hollander , and S. A. Mani , “Proactive and Reactive Roles of TGF‐β in Cancer,” Seminars in Cancer Biology 95 (2023): 120–139. 37572731 10.1016/j.semcancer.2023.08.002 PMC10530624

[14] R. Ge and G. M. Huang , “Targeting Transforming Growth Factor Beta Signaling in Metastatic Osteosarcoma,” Journal of Bone Oncology 43 (2023): 100513. 38021074 10.1016/j.jbo.2023.100513 PMC10666000

[15] R. Derynck , S. J. Turley , and R. J. Akhurst , “TGFβ Biology in Cancer Progression and Immunotherapy,” Nature Reviews Clinical Oncology 18, no. 1 (2021): 9–34. 10.1038/s41571-020-0403-1 PMC9721352 32710082

[16] Y. Zhang , L. Zhou , G. Cheng , et al., “Cordyceps sinensis Ameliorates Idiopathic Pulmonary Fibrosis in Mice via Inhibiting Mitochondrion‐mediated Oxidative Stress,” MedComm – Future Medicine 3, no. 3 (2024): e91.

[17] P. J. Sime , Z. Xing , F. L. Graham , K. G. Csaky , and J. Gauldie , “Adenovector‐mediated Gene Transfer of Active Transforming Growth Factor‐beta1 Induces Prolonged Severe Fibrosis in Rat Lung,” Journal of Clinical Investigation 100, no. 4 (1997): 768–776. 9259574 10.1172/JCI119590 PMC508247

[18] N. Inui , S. Sakai , and M. Kitagawa , “Molecular Pathogenesis of Pulmonary Fibrosis, With Focus on Pathways Related to TGF‐β and the Ubiquitin‐Proteasome Pathway,” International Journal of Molecular Sciences 22, no. 11 (2021): 6107. 34198949 10.3390/ijms22116107 PMC8201174

[19] D. Xiang , J. Zou , X. Zhu , et al., “Physalin D Attenuates Hepatic Stellate Cell Activation and Liver Fibrosis by Blocking TGF‐β/Smad and YAP Signaling,” Phytomedicine 78 (2020): 153294. 32771890 10.1016/j.phymed.2020.153294

[20] Y. Isaka , “Targeting TGF‐β Signaling in Kidney Fibrosis,” International Journal of Molecular Sciences 19, no. 9 (2018): 2532. 30150520 10.3390/ijms19092532 PMC6165001

[21] H. Khalil , O. Kanisicak , V. Prasad , et al., “Fibroblast‐specific TGF‐β‐Smad2/3 Signaling Underlies Cardiac Fibrosis,” Journal of Clinical Investigation 127, no. 10 (2017): 3770–3783. 28891814 10.1172/JCI94753 PMC5617658

[22] L. Sun , M. Xiu , S. Wang , et al., “Lipopolysaccharide Enhances TGF‐β1 Signalling Pathway and Rat Pancreatic Fibrosis,” Journal of Cellular and Molecular Medicine 22, no. 4 (2018): 2346–2356. 29424488 10.1111/jcmm.13526 PMC5867168

[23] H. Lin , B. Dong , L. Qi , et al., “Inhibitory Smads Suppress Pancreatic Stellate Cell Activation Through Negative Feedback in Chronic Pancreatitis,” Annals of Translational Medicine 9, no. 5 (2021): 384. 33842605 10.21037/atm-20-4282 PMC8033383

[24] M. Shou , H. Zhou , and L. Ma , “New Advances in Cancer Therapy Targeting TGF‐β Signaling Pathways,” Molecular Therapy Oncology 31 (2023): 100755. 10.1016/j.omto.2023.100755 PMC10746361 38144669

[25] B.‐G. Kim , E. Malek , S. H. Choi , J. J. Ignatz‐Hoover , and J. J. Driscoll , “Novel Therapies Emerging in Oncology to Target the TGF‐β Pathway,” Journal of Hematology & Oncology 14 (2021): 1–20. 33823905 10.1186/s13045-021-01053-x PMC8022551

[26] S. Liu , A. R. Khan , X. Yang , B. Dong , J. Ji , and G. Zhai , “The Reversal of Chemotherapy‐induced Multidrug Resistance by Nanomedicine for Cancer Therapy,” Journal of Controlled Release 335 (2021): 1–20. 33991600 10.1016/j.jconrel.2021.05.012

[27] F. Gsottberger , C. Meier , A. Ammon , et al., “Targeted Inhibition of Protein Synthesis Renders Cancer Cells Vulnerable to Apoptosis by Unfolded Protein Response,” Cell Death & Disease 14, no. 8 (2023): 561. 37626037 10.1038/s41419-023-06055-w PMC10457359

[28] Y. Guo , M. Ashrafizadeh , M. M. Tambuwala , J. Ren , G. Orive , and G. Yu , “P‐glycoprotein (P‐gp)‐driven Cancer Drug Resistance: Biological Profile, Non‐coding RNAs, Drugs and Nanomodulators,” Drug Discovery Today 29, no. 11 (2024): 104161. 39245345 10.1016/j.drudis.2024.104161

[29] K. Gach‐Janczak , J. Drogosz‐Stachowicz , A. Janecka , K. Wtorek , and M. Mirowski , “Historical Perspective and Current Trends in Anticancer Drug Development,” Cancers (Basel) 16, no. 10 (2024): 1878. 38791957 10.3390/cancers16101878 PMC11120596

[30] A. L. Parker , M. Benguigui , J. Fornetti , et al., “Current Challenges in Metastasis Research and Future Innovation for Clinical Translation,” Clinical & Experimental Metastasis 39, no. 2 (2022): 263–277. 35072851 10.1007/s10585-021-10144-5 PMC8971179

[31] A. Naeem , P. Hu , M. Yang , et al., “Natural Products as Anticancer Agents: Current Status and Future Perspectives,” Molecules (Basel, Switzerland) 27, no. 23 (2022): 8367. 36500466 10.3390/molecules27238367 PMC9737905

[32] F. C. Cadoná , R. F. Dantas , G. H. de Mello , and F. P. Silva Jr , “Natural Products Targeting Into Cancer Hallmarks: An Update on Caffeine, Theobromine, and (+)‐catechin,” Critical Reviews in Food Science and Nutrition 62, no. 26 (2022): 7222–7241. 33890518 10.1080/10408398.2021.1913091

[33] P. Chunarkar‐Patil , M. Kaleem , R. Mishra , et al., “Anticancer Drug Discovery Based on Natural Products: From Computational Approaches to Clinical Studies,” Biomedicines 12, no. 1 (2024): 201. 38255306 10.3390/biomedicines12010201 PMC10813144

[34] S. Motyka , K. Jafernik , H. Ekiert , et al., “Podophyllotoxin and Its Derivatives: Potential Anticancer Agents of Natural Origin in Cancer Chemotherapy,” Biomedicine & Pharmacotherapy 158 (2023): 114145. 36586242 10.1016/j.biopha.2022.114145

[35] D. Kitic , B. Miladinovic , M. Randjelovic , et al., “Anticancer and Chemopreventive Potential of Morinda Citrifolia L. bioactive Compounds: A Comprehensive Update,” Phytotherapy Research 38, no. 4 (2024): 1932–1950. 38358681 10.1002/ptr.8137

[36] L. Hardt , Y. Mahamat‐Saleh , D. Aune , and S. Schlesinger , “Plant‐Based Diets and Cancer Prognosis: A Review of Recent Research,” Current Nutrition Reports 11, no. 4 (2022): 695–716. 36138327 10.1007/s13668-022-00440-1 PMC9750928

[37] M. L. Coêlho , M. T. Islam , G. Laylson da Silva Oliveira , et al., “Cytotoxic and Antioxidant Properties of Natural Bioactive Monoterpenes Nerol, Estragole, and 3,7‐Dimethyl‐1‐Octanol,” Advances in Pharmacological and Pharmaceutical Sciences 2022 (2022): 8002766–8002766. 36465700 10.1155/2022/8002766 PMC9712021

[38] R. Hossain , P. Ray , C. Sarkar , et al., “Natural Compounds or Their Derivatives Against Breast Cancer: A Computational Study,” BioMed Research International 2022 (2022): 5886269–5886269. 35837379 10.1155/2022/5886269 PMC9276515

[39] G. Peron , A. Mastinu , S. I. Peña‐Corona , et al., “Silvestrol, a Potent Anticancer Agent With Unfavourable Pharmacokinetics: Current Knowledge on Its Pharmacological Properties and Future Directions for the Development of Novel Drugs,” Biomedicine & Pharmacotherapy 177 (2024): 117047. 38959604 10.1016/j.biopha.2024.117047

[40] M. Kamle , S. Pandhi , S. Mishra , et al., “Camptothecin and Its Derivatives: Advancements, Mechanisms and Clinical Potential in Cancer Therapy,” Medical Oncology 41, no. 11 (2024): 263. 39382779 10.1007/s12032-024-02527-x

[41] K. A. Mansour , A. Elbermawi , A. A. Al‐Karmalawy , M. F. Lahloub , and M. El‐Neketi , “Cytotoxic Effects of Extracts Obtained From Plants of the Oleaceae family: Bio‐guided Isolation and Molecular Docking of New Secoiridoids From Jasminum Humile,” Pharmaceutical Biology 60, no. 1 (2022): 1374–1383. 35961303 10.1080/13880209.2022.2098346 PMC9377236

[42] Z. Javed , K. Khan , J. Herrera‐Bravo , et al., “Myricetin: Targeting Signaling Networks in Cancer and Its Implication in Chemotherapy,” Cancer Cell International 22, no. 1 (2022): 239. 35902860 10.1186/s12935-022-02663-2 PMC9336020

[43] D. Liu , A. P. van der Zalm , J. Koster , et al., “Predictive Biomarkers for Response to TGF‐ β Inhibition in Resensitizing Chemo(radiated) Esophageal Adenocarcinoma,” Pharmacological Research 207 (2024): 107315. 39059615 10.1016/j.phrs.2024.107315

[44] M. H. Barcellos‐Hoff and J. L. Gulley , “Molecular Pathways and Mechanisms of TGFβ in Cancer Therapy,” Clinical Cancer Research 29, no. 11 (2023): 2025–2033. 36598437 10.1158/1078-0432.CCR-21-3750 PMC10238558

[45] R. Derynck , S. J. Turley , and R. J. Akhurst , “TGFbeta Biology in Cancer Progression and Immunotherapy,” Nature Reviews Clinical Oncology 18, no. 1 (2021): 9–34. 10.1038/s41571-020-0403-1 PMC9721352 32710082

[46] M. Turati , A. Mousset , N. Issa , A. Turtoi , and R. Ronca , “TGF‐β Mediated Drug Resistance in Solid Cancer,” Cytokine & Growth Factor Reviews 71‐72 (2023): 54–65. 10.1016/j.cytogfr.2023.04.001 37100675

[47] A. Gazzillo , M. A. Polidoro , C. Soldani , B. Franceschini , A. Lleo , and M. Donadon , “Relationship Between Epithelial‐to‐Mesenchymal Transition and Tumor‐Associated Macrophages in Colorectal Liver Metastases,” International Journal of Molecular Sciences 23, no. 24 (2022): 16197. 36555840 10.3390/ijms232416197 PMC9783529

[48] A. O. Giarratana , C. M. Prendergast , M. M. Salvatore , and K. M. Capaccione , “TGF‐β Signaling: Critical Nexus of Fibrogenesis and Cancer,” Journal of Translational Medicine 22, no. 1 (2024): 594. 38926762 10.1186/s12967-024-05411-4 PMC11201862

[49] Z. Deng , T. Fan , C. Xiao , et al., “TGF‐β Signaling in Health, Disease, and Therapeutics,” Signal Transduction and Targeted Therapy 9, no. 1 (2024): 61. 38514615 10.1038/s41392-024-01764-w PMC10958066

[50] Y. Yang , W. L. Ye , R. N. Zhang , et al., “The Role of TGF‐beta Signaling Pathways in Cancer and Its Potential as a Therapeutic Target,” Evidence‐Based Complementary and Alternative Medicine 2021 (2021): 6675208. 34335834 10.1155/2021/6675208 PMC8321733

[51] M. Abbastabar , M. Kheyrollah , K. Azizian , et al., “Multiple Functions of p27 in Cell Cycle, Apoptosis, Epigenetic Modification and Transcriptional Regulation for the Control of Cell Growth: A Double‐edged Sword Protein,” Dna Repair 69 (2018): 63–72. 30075372 10.1016/j.dnarep.2018.07.008

[52] C. Petritsch , H. Beug , A. Balmain , and M. Oft , “TGF‐beta Inhibits p70 S6 Kinase via Protein Phosphatase 2A to Induce G(1) Arrest,” Genes & Development 14, no. 24 (2000): 3093–3101. 11124802 10.1101/gad.854200 PMC317138

[53] Q. Wang , F. Xiong , G. Wu , et al., “SMAD Proteins in TGF‐β Signalling Pathway in Cancer: Regulatory Mechanisms and Clinical Applications,” Diagnostics (Basel) 13, no. 17 (2023): 2769. 37685308 10.3390/diagnostics13172769 PMC10487229

[54] A. Glaviano , A. S. C. Foo , H. Y. Lam , et al., “PI3K/AKT/mTOR Signaling Transduction Pathway and Targeted Therapies in Cancer,” Molecular Cancer 22, no. 1 (2023): 138. 37596643 10.1186/s12943-023-01827-6 PMC10436543

[55] M. Kammoun , J. Piquereau , L. Nadal‐Desbarats , et al., “Novel Role of Tieg1 in Muscle Metabolism and Mitochondrial Oxidative Capacities,” Acta Physiologica (Oxford) 228, no. 3 (2020): e13394. 10.1111/apha.13394 31560161

[56] A. B. Baba , B. Rah , G. R. Bhat , et al., “Transforming Growth Factor‐Beta (TGF‐beta) Signaling in Cancer‐A Betrayal within,” Frontiers in Pharmacology 13 (2022): 791272. 35295334 10.3389/fphar.2022.791272 PMC8918694

[57] M. Zhao , L. Mishra , and C. X. Deng , “The Role of TGF‐β/SMAD4 Signaling in Cancer,” International Journal of Biological Sciences 14, no. 2 (2018): 111–123. 29483830 10.7150/ijbs.23230 PMC5821033

[58] J. Dardare , A. Witz , J. L. Merlin , P. Gilson , and A. Harlé , “SMAD4 and the TGFβ Pathway in Patients With Pancreatic Ductal Adenocarcinoma,” International Journal of Molecular Sciences 21, no. 10 (2020): 3534. 32429474 10.3390/ijms21103534 PMC7278913

[59] V. W. Xue , J. Y. Chung , C. A. G. Cordoba , et al., “Transforming Growth Factor‐beta: A Multifunctional Regulator of Cancer Immunity,” Cancers (Basel) 12, no. 11 (2020): 3099. 33114183 10.3390/cancers12113099 PMC7690808

[60] B. G. Kim , E. Malek , S. H. Choi , J. J. Ignatz‐Hoover , and J. J. Driscoll , “Novel Therapies Emerging in Oncology to Target the TGF‐beta Pathway,” Journal of Hematology & Oncology 14, no. 1 (2021): 55. 33823905 10.1186/s13045-021-01053-x PMC8022551

[61] R. Vogelmann , M. D. Nguyen‐Tat , K. Giehl , G. Adler , D. Wedlich , and A Menke . TGFbeta‐induced Downregulation of E‐cadherin‐based Cell‐cell Adhesion Depends on PI3‐kinase and PTEN. Journal of Cell Science 2005;118(Pt 20):4901–4912. 16219695 10.1242/jcs.02594

[62] Q. Zhang , B. T. Helfand , T. L. Jang , et al. Nuclear Factor‐kappaB‐mediated Transforming Growth Factor‐beta‐induced Expression of vimentin Is an Independent Predictor of Biochemical Recurrence After Radical Prostatectomy. Clinical Cancer Research 2009;15(10):3557–3567. 19447876 10.1158/1078-0432.CCR-08-1656

[63] Y. Wu , X. Zhang , M. Salmon , X. Lin , and Z. E. Zehner , “TGFbeta1 regulation of Vimentin Gene Expression During Differentiation of the C2C12 Skeletal Myogenic Cell Line Requires Smads, AP‐1 and Sp1 family Members,” Biochimica Et Biophysica Acta 1773, no. 3 (2007): 427–439. 17270292 10.1016/j.bbamcr.2006.11.017 PMC1855268

[64] J. Xu , S. Lamouille , and R. Derynck , “TGF‐beta‐induced Epithelial to Mesenchymal Transition,” Cell Research 19, no. 2 (2009): 156–172. 19153598 10.1038/cr.2009.5 PMC4720263

[65] M. Adorno , M. Cordenonsi , M. Montagner , et al., “A Mutant‐p53/Smad Complex Opposes p63 to Empower TGFbeta‐induced Metastasis,” Cell 137, no. 1 (2009): 87–98. 19345189 10.1016/j.cell.2009.01.039

[66] T. Shimo , T. Nakanishi , T. Nishida , et al., “Involvement of CTGF, a Hypertrophic Chondrocyte‐specific Gene Product, in Tumor Angiogenesis,” Oncology 61, no. 4 (2001): 315–322. 11721179 10.1159/000055339

[67] J. Wang , Y. Wang , Y. Wang , Y. Ma , Y. Lan , and X. Yang , “Transforming Growth Factor Beta‐regulated microRNA‐29a Promotes Angiogenesis Through Targeting the Phosphatase and Tensin Homolog in Endothelium,” Journal of Biological Chemistry 288, no. 15 (2013): 10418–10426. 23426367 10.1074/jbc.M112.444463 PMC3624424

[68] A. Safina , E. Vandette , and A. V. Bakin , “ALK5 promotes Tumor Angiogenesis by Upregulating Matrix Metalloproteinase‐9 in Tumor Cells,” Oncogene 26, no. 17 (2007): 2407–2422. 17072348 10.1038/sj.onc.1210046

[69] E. Vivier , E. Tomasello , M. Baratin , T. Walzer , and S. Ugolini , “Functions of Natural Killer Cells,” Nature Immunology 9, no. 5 (2008): 503–510. 18425107 10.1038/ni1582

[70] S. Regis , A. Dondero , F. Caliendo , C. Bottino , and R. Castriconi , “NK Cell Function Regulation by TGF‐beta‐Induced Epigenetic Mechanisms,” Frontiers in Immunology 11 (2020): 311. 32161594 10.3389/fimmu.2020.00311 PMC7052483

[71] Z. Li , D. Li , A. Tsun , and B. Li , “FOXP3+ regulatory T Cells and Their Functional Regulation,” Cellular & Molecular Immunology 12, no. 5 (2015): 558–565. 25683611 10.1038/cmi.2015.10 PMC4579651

[72] M. Gachpazan , H. Kashani , S. M. Hassanian , et al., “Therapeutic Potential of Targeting Transforming Growth Factor‐beta in Colorectal Cancer: Rational and Progress,” Current Pharmaceutical Design 25, no. 38 (2019): 4085–4089. 31692434 10.2174/1381612825666191105114539

[73] J. Y. Son , S. Y. Park , S. J. Kim , et al., “EW‐7197, a Novel ALK‐5 Kinase Inhibitor, Potently Inhibits Breast to Lung Metastasis,” Molecular Cancer Therapeutics 13, no. 7 (2014): 1704–1716. 24817629 10.1158/1535-7163.MCT-13-0903

[74] S. Herbertz , J. S. Sawyer , A. J. Stauber , et al., “Clinical Development of galunisertib (LY2157299 monohydrate), a Small Molecule Inhibitor of Transforming Growth Factor‐beta Signaling Pathway,” Drug Design, Development and Therapy 9 (2015): 4479–4499. 26309397 10.2147/DDDT.S86621 PMC4539082

[75] Y. Tie , F. Tang , D. Peng , Y. Zhang , and H. Shi , “TGF‐beta Signal Transduction: Biology, Function and Therapy for Diseases,” Molecular Biomed 3, no. 1 (2022): 45. 10.1186/s43556-022-00109-9 PMC9761655 36534225

[76] M. S. Gordon , R. Ilaria Jr. , D. P. de Alwis , et al., “A Phase I Study of Tasisulam Sodium (LY573636 sodium), a Novel Anticancer Compound, Administered as a 24‐h Continuous Infusion in Patients With Advanced Solid Tumors,” Cancer Chemotheraphy and Pharmacology 71, no. 1 (2013): 21–27. 10.1007/s00280-012-1917-8 23228983

[77] M. Tojo , Y. Hamashima , A. Hanyu , et al., “The ALK‐5 Inhibitor A‐83‐01 Inhibits Smad Signaling and Epithelial‐to‐mesenchymal Transition by Transforming Growth Factor‐beta,” Cancer Science 96, no. 11 (2005): 791–800. 16271073 10.1111/j.1349-7006.2005.00103.x PMC11159601

[78] S. Matsuyama , M. Iwadate , M. Kondo , et al., “SB‐431542 and Gleevec Inhibit Transforming Growth Factor‐beta‐induced Proliferation of human Osteosarcoma Cells,” Cancer Research 63, no. 22 (2003): 7791–7798. 14633705

[79] C. J. Martin , A. Datta , C. Littlefield , et al., “Selective Inhibition of TGFbeta1 Activation Overcomes Primary Resistance to Checkpoint Blockade Therapy by Altering Tumor Immune Landscape,” Science Translational Medicine 12, no. 536 (2020): eaay8456. 32213632 10.1126/scitranslmed.aay8456

[80] J. C. Morris , A. R. Tan , T. E. Olencki , et al., “Phase I Study of GC1008 (fresolimumab): A human Anti‐transforming Growth Factor‐beta (TGFbeta) Monoclonal Antibody in Patients With Advanced Malignant Melanoma or Renal Cell Carcinoma,” PLoS ONE 9, no. 3 (2014): e90353. 24618589 10.1371/journal.pone.0090353 PMC3949712

[81] A. W. Tolcher , J. D. Berlin , J. Cosaert , et al., “A Phase 1 Study of Anti‐TGFbeta Receptor Type‐II Monoclonal Antibody LY3022859 in Patients With Advanced Solid Tumors,” Cancer Chemotheraphy and Pharmacology 79, no. 4 (2017): 673–680. 10.1007/s00280-017-3245-5 PMC5893148 28280971

[82] K. M. Moore , G. J. Thomas , S. W. Duffy , et al., “Therapeutic Targeting of Integrin alphavbeta6 in Breast Cancer,” JNCI: Journal of the National Cancer Institute 106, no. 8 (2014): dju169. 24974129 10.1093/jnci/dju169 PMC4151855

[83] A. Bandyopadhyay , F. Lopez‐Casillas , S. N. Malik , et al., “Antitumor Activity of a Recombinant Soluble Betaglycan in human Breast Cancer Xenograft,” Cancer Research 62, no. 16 (2002): 4690–4695. 12183427

[84] P. Fenaux , U. Platzbecker , G. J. Mufti , et al., “Luspatercept in Patients With Lower‐Risk Myelodysplastic Syndromes,” New England Journal of Medicine 382, no. 2 (2020): 140–151. 31914241 10.1056/NEJMoa1908892

[85] M. E. Gleave and B. P. Monia , “Antisense Therapy for Cancer,” Nature Reviews Cancer 5, no. 6 (2005): 468–479. 15905854 10.1038/nrc1631

[86] K. H. Schlingensiepen , R. Schlingensiepen , A. Steinbrecher , et al., “Targeted Tumor Therapy With the TGF‐beta 2 Antisense Compound AP 12009,” Cytokine & Growth Factor Reviews 17, no. 1‐2 (2006): 129–139. 16377233 10.1016/j.cytogfr.2005.09.002

[87] F. Jaschinski , T. Rothhammer , P. Jachimczak , C. Seitz , A. Schneider , and K. H. Schlingensiepen , “The Antisense Oligonucleotide Trabedersen (AP 12009) for the Targeted Inhibition of TGF‐beta2,” Current Pharmaceutical Biotechnology 12, no. 12 (2011): 2203–2213. 21619536 10.2174/138920111798808266

[88] P. Lampropoulos , A. Zizi‐Sermpetzoglou , S. Rizos , A. Kostakis , N. Nikiteas , and A. G. Papavassiliou , “TGF‐beta Signalling in Colon Carcinogenesis,” Cancer Letters 314, no. 1 (2012): 1–7. 22018778 10.1016/j.canlet.2011.09.041

[89] P. Hau , P. Jachimczak , and U. Bogdahn , “Treatment of Malignant Gliomas With TGF‐beta2 Antisense Oligonucleotides,” Expert Review of Anticancer Therapy 9, no. 11 (2009): 1663–1674. 19895249 10.1586/era.09.138

[90] S. Khatua , S. Nandi , A. Nag , et al., “Homoharringtonine: Updated Insights Into Its Efficacy in Hematological Malignancies, Diverse Cancers and Other Biomedical Applications,” European Journal of Medical Research 29, no. 1 (2024): 269. 38704602 10.1186/s40001-024-01856-x PMC11069164

[91] J. Chen , Q. Mu , X. Li , et al., “Homoharringtonine Targets Smad3 and TGF‐β Pathway to Inhibit the Proliferation of Acute Myeloid Leukemia Cells,” Oncotarget 8, no. 25 (2017): 40318–40326. 28454099 10.18632/oncotarget.16956 PMC5522237

[92] B. Li , J. Zhao , C. Z. Wang , et al., “Ginsenoside Rh2 Induces Apoptosis and Paraptosis‐Like Cell Death in Colorectal Cancer Cells Through Activation of p53,” Cancer Letters 301, no. 2 (2011): 185–192. 21194832 10.1016/j.canlet.2010.11.015 PMC3022099

[93] Q. R. Hu , Y. Pan , H. C. Wu , et al., “The Ways for Ginsenoside Rh2 to Fight Against Cancer: The Molecular Evidences in Vitro and in Vivo,” Journal of Ginseng Research 47, no. 2 (2023): 173–182. 36926617 10.1016/j.jgr.2022.09.011 PMC10014223

[94] D. Wang , M. Tian , Y. Fu , et al., “Halofuginone Inhibits Tumor Migration and Invasion by Affecting Cancer‐associated Fibroblasts in Oral Squamous Cell Carcinoma. Original Research,” Frontiers in Pharmacology 13 (2022): 1056337. 36506509 10.3389/fphar.2022.1056337 PMC9726898

[95] P. A. Assis , L. L. De Figueiredo‐Pontes , A. S. G. Lima , et al., “Halofuginone Inhibits Phosphorylation of SMAD‐2 Reducing Angiogenesis and Leukemia Burden in an Acute Promyelocytic Leukemia Mouse Model,” Journal of Experimental & Clinical Cancer Research 34, no. 1 (2015): 65. 26099922 10.1186/s13046-015-0181-2 PMC4486128

[96] L. L. de Figueiredo‐Pontes , P. A. Assis , B. A. A. Santana‐Lemos , et al., “Halofuginone Has Anti‐Proliferative Effects in Acute Promyelocytic Leukemia by Modulating the Transforming Growth Factor Beta Signaling Pathway,” PLoS ONE 6, no. 10 (2011): e26713. 22053203 10.1371/journal.pone.0026713 PMC3203897

[97] M. Satoh , Y. Takemura , H. Hamada , Y. Sekido , and S. Kubota , “EGCG Induces human Mesothelioma Cell Death by Inducing Reactive Oxygen Species and Autophagy,” Cancer Cell International 13, no. 1 (2013): 19. 23432995 10.1186/1475-2867-13-19 PMC3605250

[98] B. Goker , C. Caliskan , H. Onur Caglar , et al., “Synergistic Effect of Ponatinib and Epigallocatechin‐3‐gallate Induces Apoptosis in Chronic Myeloid Leukemia Cells Through Altering Expressions of Cell Cycle Regulatory Genes,” Journal of B.U.ON 19, no. 4 (2014): 992–998. 25536607

[99] L. Zhao , S. Liu , X. Che , et al., “Bufalin Inhibits TGF‐β‐induced Epithelial‐to‐mesenchymal Transition and Migration in human Lung Cancer A549 Cells by Downregulating TGF‐β Receptors,” International Journal of Molecular Medicine 36, no. 3 (2015): 645–652. 26133118 10.3892/ijmm.2015.2268 PMC4533784

[100] K.‐S. Chung , S.‐H. Cho , J.‐S. Shin , et al., “Ginsenoside Rh2 Induces Cell Cycle Arrest and Differentiation in human Leukemia Cells by Upregulating TGF‐β Expression,” Carcinogenesis 34, no. 2 (2012): 331–340. 23125221 10.1093/carcin/bgs341

[101] L. Shen , L.‐L. Zhang , H. Li , et al., “Oroxylin A Inhibits the Generation of Tregs in Non‐small Cell Lung Cancer,” Oncotarget 8, no. 30 (2017): 49395. 28472762 10.18632/oncotarget.17218 PMC5564777

[102] Z. Faisal , A. Mazhar , S. A. Batool , et al., “Exploring the Multimodal Health‐promoting Properties of resveratrol: A Comprehensive Review,” Food Science & Nutrition 12, no. 4 (2024): 2240–2258. 38628180 10.1002/fsn3.3933 PMC11016399

[103] H. Wang , H. Zhang , L. Tang , et al., “Resveratrol Inhibits TGF‐β1‐induced Epithelial‐to‐mesenchymal Transition and Suppresses Lung Cancer Invasion and Metastasis,” Toxicology 303 (2013): 139–146. 23146760 10.1016/j.tox.2012.09.017

[104] G. Han , Y. Wang , T. Liu , et al., “Salvianolic Acid B Acts Against Non‑Small Cell Lung Cancer A549 Cells via Inactivation of the MAPK and Smad2/3 Signaling Pathways,” Molecular Medicine Reports 25, no. 5 (2022): 1–11. 35348194 10.3892/mmr.2022.12700 PMC8985201

[105] F. Hai‐Tao , Z. Wen‐Wen , L. Jin‐Jian , W. Yi‐Tao , and C. Xiu‐Ping , “Hypaconitine Inhibits TGF‐β1‐induced Epithelial–mesenchymal Transition and Suppresses Adhesion, Migration, and Invasion of Lung Cancer A549 Cells,” Chinese Journal of Natural Medicines 15, no. 6 (2017): 427–435. 28629532 10.1016/S1875-5364(17)30064-X

[106] E. Jo , S. J. Park , Y. S. Choi , W.‐K. Jeon , and B.‐C. Kim , “Kaempferol Suppresses Transforming Growth Factor‐β1–induced Epithelial‐to‐mesenchymal Transition and Migration of A549 Lung Cancer Cells by Inhibiting Akt1‐mediated Phosphorylation of Smad3 at Threonine‐179,” Neoplasia 17, no. 7 (2015): 525–537. 26297431 10.1016/j.neo.2015.06.004 PMC4547409

[107] J. S. Ruan , H. Zhou , L. Yang , et al., “Ursolic Acid Attenuates TGF‐β1‐Induced Epithelial–Mesenchymal Transition in NSCLC by Targeting Integrin αVβ5/MMPs Signaling,” Oncology Research 27, no. 5 (2019): 593. 28911340 10.3727/096504017X15051723858706 PMC7848462

[108] S. Dai , C. Wang , X. Zhao , et al., “Cucurbitacin B: A Review of Its Pharmacology, Toxicity, and Pharmacokinetics,” Pharmacological Research 187 (2023): 106587. 36460279 10.1016/j.phrs.2022.106587

[109] R. Yuan , Q. Fan , X. Liang , et al., “Cucurbitacin B Inhibits TGF‐β1‐induced Epithelial–mesenchymal Transition (EMT) in NSCLC Through Regulating ROS and PI3K/Akt/mTOR Pathways,” Chinese Medicine 17, no. 1 (2022): 24. 35183200 10.1186/s13020-022-00581-z PMC8858510

[110] G. De Rubis , K. R. Paudel , G. Liu , et al., “Berberine‐loaded Engineered Nanoparticles Attenuate TGF‐β‐induced Remodelling in human Bronchial Epithelial Cells,” Toxicology in Vitro 92 (2023): 105660. 37591407 10.1016/j.tiv.2023.105660

[111] R. Hossain , C. Quispe , J. Herrera‐Bravo , et al., “Neurobiological Promises of the Bitter Diterpene Lactone Andrographolide,” Oxidative Medicine and Cellular Longevity 2022 (2022): 3079577. 35154564 10.1155/2022/3079577 PMC8825670

[112] H.‐H. Lin , C.‐W. Tsai , F.‐P. Chou , et al., “Andrographolide Down‐regulates Hypoxia‐inducible Factor‐1α in human Non‐small Cell Lung Cancer A549 Cells,” Toxicology and Applied Pharmacology 250, no. 3 (2011): 336–345. 21134392 10.1016/j.taap.2010.11.014

[113] C. Da , Y. Liu , Y. Zhan , K. Liu , and R. Wang , “Nobiletin Inhibits Epithelial‐mesenchymal Transition of human Non‐small Cell Lung Cancer Cells by Antagonizing the TGF‐β1/Smad3 Signaling Pathway,” Oncology Reports 35, no. 5 (2016): 2767–2774. 26986176 10.3892/or.2016.4661

[114] P. C. Zhong , Z. W. Liu , Q. C. Xing , J. Chen , and R. P. Yang , “Neferine Inhibits the Development of Lung Cancer Cells by Downregulating TGF‐β to Regulate MST1/ROS‐induced Pyroptosis,” The Kaohsiung Journal of Medical Sciences 39, no. 11 (2023): 1106–1118. 37698291 10.1002/kjm2.12752 PMC11895869

[115] M.‐W. Byun , “Schizonepeta tenuifolia Ethanol Extract Exerts Anti‐inflammatory Activity Through the Inhibition of TLR4 Signaling in Lipopolysaccharide‐stimulated Macrophage Cells,” Journal of Medicinal Food 17, no. 3 (2014): 350–356. 24650252 10.1089/jmf.2013.2928

[116] J. Li , Y. Cheng , W. Qu , et al., “Fisetin, a Dietary Flavonoid, Induces Cell Cycle Arrest and Apoptosis Through Activation of p53 and Inhibition of NF‐kappa B Pathways in Bladder Cancer Cells,” Basic & Clinical Pharmacology & Toxicology 108, no. 2 (2011): 84–93. 21054790 10.1111/j.1742-7843.2010.00613.x

[117] X.‐F. Liu , H.‐J. Long , X.‐Y. Miao , G.‐L. Liu , and H.‐L. Yao , “Fisetin Inhibits Liver Cancer Growth in a Mouse Model: Relation to Dopamine Receptor,” Oncology Reports 38, no. 1 (2017): 53–62. 28560391 10.3892/or.2017.5676 PMC5492805

[118] D.‐R. Pang , X.‐Q. Su , Z.‐X. Zhu , et al., “Flavonoid Dimers From the Total Phenolic Extract of Chinese Dragon's Blood, the Red Resin of Dracaena Cochinchinensis,” Fitoterapia 115 (2016): 135–141. 27769819 10.1016/j.fitote.2016.10.004

[119] Q. Yuan , C. Qu , and C. Liu , “Summary of the Modern Clinical Application of Resina Draconis,” Medicinal Plant 4, no. 8 (2013): 64.

[120] X. Chen , Y. Zhao , A. Yang , et al., “Chinese Dragon's Blood EtOAc Extract Inhibits Liver Cancer Growth Through Downregulation of Smad3,” Frontiers in Pharmacology 11 (2020): 530347. 10.3389/fphar.2020.00669 PMC7237706 32477135

[121] L. Ma , H. Jiang , X. Xu , et al., “Tanshinone IIA Mediates SMAD7‐YAP Interaction to Inhibit Liver Cancer Growth by Inactivating the Transforming Growth Factor Beta Signaling Pathway,” Aging (Albany NY) 11, no. 21 (2019): 9719. 31711043 10.18632/aging.102420 PMC6874425

[122] L. Lu , Q. Guo , and L. Zhao , “Overview of Oroxylin A: A Promising Flavonoid Compound,” Phytotherapy Research 30, no. 11 (2016): 1765–1774. 27539056 10.1002/ptr.5694

[123] H.‐B. Li and F. Chen , “Isolation and Purification of Baicalein, Wogonin and Oroxylin A From the Medicinal Plant Scutellaria baicalensis by High‐speed Counter‐current Chromatography,” Journal of Chromatography A 1074, no. 1‐2 (2005): 107–110. 15941045 10.1016/j.chroma.2005.03.088

[124] Y. Sun , N. Lu , Y. Ling , et al., “Oroxylin A Suppresses Invasion Through Down‐regulating the Expression of Matrix Metalloproteinase‐2/9 in MDA‐MB‐435 human Breast Cancer Cells,” European Journal of Pharmacology 603, no. 1‐3 (2009): 22–28. 19100732 10.1016/j.ejphar.2008.12.008

[125] L. Wei , Y. Yao , K. Zhao , et al., “Oroxylin A Inhibits Invasion and Migration Through Suppressing ERK/GSK‐3β Signaling in Snail‐expressing Non‐small‐cell Lung Cancer Cells,” Molecular Carcinogenesis 55, no. 12 (2016): 2121–2134. 26741501 10.1002/mc.22456

[126] T.‐X. Huo , X.‐P. Wang , Z. Yu , et al., “Oroxylin A Inhibits the Migration of Hepatocellular Carcinoma Cells by Inducing NAG‐1 Expression,” Acta Pharmacologica Sinica 43, no. 3 (2022): 724–734. 34117368 10.1038/s41401-021-00695-4 PMC8888648

[127] M. Asif Ali , N. Khan , N. Kaleem , et al., “Anticancer Properties of sulforaphane: Current Insights at the Molecular Level,” Frontiers in Oncology 13 (2023): 1168321. 37397365 10.3389/fonc.2023.1168321 PMC10313060

[128] J. Wu , J. Han , B. Hou , C. Deng , H. Wu , and L. Shen , “Sulforaphane Inhibits TGF‐β‐induced Epithelial‐mesenchymal Transition of Hepatocellular Carcinoma Cells via the Reactive Oxygen Species‐dependent Pathway,” Oncology Reports 35, no. 5 (2016): 2977–2983. 26935987 10.3892/or.2016.4638

[129] N. Zhang , Y. Hu , C. Ding , et al., “Salvianolic Acid B Protects Against Chronic Alcoholic Liver Injury via SIRT1‐mediated Inhibition of CRP and ChREBP in Rats,” Toxicology Letters 267 (2017): 1–10. 27989594 10.1016/j.toxlet.2016.12.010

[130] Y. Gong , D. Li , L. Li , et al., “Smad3 C‐terminal Phosphorylation Site Mutation Attenuates the Hepatoprotective Effect of Salvianolic Acid B Against Hepatocarcinogenesis,” Food and Chemical Toxicology 147 (2021): 111912. 33290806 10.1016/j.fct.2020.111912

[131] X.‐N. Liu , S. Wang , Q. Yang , Y.‐J. Wang , D.‐X. Chen , and X.‐X. Zhu , “ESC Reverses Epithelial Mesenchymal Transition Induced by Transforming Growth Factor‐β via Inhibition of Smad Signal Pathway in HepG2 Liver Cancer Cells,” Cancer Cell International 15 (2015): 1–10. 26692820 10.1186/s12935-015-0265-2 PMC4676109

[132] Y.‐J. Lee , E.‐S. Kao , C.‐Y. Chu , W.‐L. Lin , Y.‐H. Chiou , and T.‐H. Tseng , “Inhibitory Effect of Ailanthoidol on 12‐O‐tetradecanoyl‐phorbol‐13‐acetate‐induced Tumor Promotion in Mouse Skin,” Oncology Reports 16, no. 4 (2006): 921–927. 16969515

[133] J. K. Kim and J. G. Jun , “Ailanthoidol Suppresses Lipopolysaccharide‐stimulated Inflammatory Reactions in RAW264. 7 Cells and Endotoxin Shock in Mice,” Journal of Cellular Biochemistry 112, no. 12 (2011): 3816–3823. 21826708 10.1002/jcb.23312

[134] J.‐H. Park , J.‐G. Jun , and J.‐K. Kim , “Anti‐Adipogenic Activity of Ailanthoidol on 3T3‐L1 Adipocytes,” Biomedical Science Letters 20, no. 2 (2014): 62–69.

[135] T. Tseng , H. Lee , and Y. Lee , “Ailanthoidol, a Neolignan, Suppresses TGF‐β1‐induced HepG2 Hepatoblastoma Cell Progression,” Biomedicines 9 (2021): 1110. 34572296 10.3390/biomedicines9091110 PMC8472484

[136] C.‐W. Lu , S.‐K. Huang , T.‐Y. Lin , and S.‐J. Wang , “Echinacoside, an Active Constituent of Herba Cistanche, Suppresses Epileptiform Activity in Hippocampal CA3 Pyramidal Neurons,” The Korean Journal of Physiology & Pharmacology: Official Journal of the Korean Physiological Society and the Korean Society of Pharmacology 22, no. 3 (2018): 249. 29719447 10.4196/kjpp.2018.22.3.249 PMC5928338

[137] W. Li , J. Zhou , Y. Zhang , et al., “Echinacoside Exerts Anti‐tumor Activity via the miR‐503‐3p/TGF‐β1/Smad Aixs in Liver Cancer,” Cancer Cell International 21, no. 1 (2021): 304. 34112163 10.1186/s12935-021-01890-3 PMC8191129

[138] S. Liang , Y. Zou , J. Gao , et al., “The Chinese Medicine, Jiedu Recipe, Inhibits the Epithelial Mesenchymal Transition of Hepatocellular Carcinoma via the Regulation of smad2/3 Dependent and Independent Pathways,” Evidence‐Based Complementary and Alternative Medicine 2018 (2018): 9780826. 10.1155/2018/5629304 PMC6106903 30174709

[139] J. Gao , Y. Ying , J. Wang , and Y. Cui , “Solanine Inhibits Immune Escape Mediated by Hepatoma Treg Cells via the Tgfβ/Smad Signaling Pathway,” BioMed Research International 2020 (2020): 9749631. 33204731 10.1155/2020/9749631 PMC7655262

[140] L. Zhang , Q. Y. Cai , J. Liu , et al., “Ursolic Acid Suppresses the Invasive Potential of Colorectal Cancer Cells by Regulating the TGF‐beta1/ZEB1/miR‐200c Signaling Pathway,” Oncology Letters 18, no. 3 (2019): 3274–3282. 31452805 10.3892/ol.2019.10604 PMC6676672

[141] L. L. Coutinho , T. C. T. Junior , and M. C. Rangel , “Sulforaphane: An Emergent Anti‐cancer Stem Cell Agent,” Frontiers in Oncology 13 (2023): 1089115. 36776295 10.3389/fonc.2023.1089115 PMC9909961

[142] A. Mahn and A. Castillo , “Potential of Sulforaphane as a Natural Immune System Enhancer: A Review,” Molecules (Basel, Switzerland) 26, no. 3 (2021): 752. 33535560 10.3390/molecules26030752 PMC7867070

[143] B. M. Kaminski , S. M. Loitsch , M. J. Ochs , et al., “Isothiocyanate Sulforaphane Inhibits Protooncogenic Ornithine Decarboxylase Activity in Colorectal Cancer Cells via Induction of the TGF‐beta/Smad Signaling Pathway,” Molecular Nutrition & Food Research 54, no. 10 (2010): 1486–1496. 20603835 10.1002/mnfr.201000105

[144] Q. Hu , W. Zhang , Z. Wu , et al., “Baicalin and the Liver‐gut System: Pharmacological Bases Explaining Its Therapeutic Effects,” Pharmacological Research 165 (2021): 105444. 33493657 10.1016/j.phrs.2021.105444

[145] X. Guan , S. Shen , J. Liu , et al., “Protective Effecs of Baicalin Magnesium on Non‐alcoholic Steatohepatitis Rats Are Based on Inhibiting NLRP3/Caspase‐1/IL‐1beta Signaling Pathway,” BMC Complementary Medicine and Therapies 23, no. 1 (2023): 72. 36879310 10.1186/s12906-023-03903-2 PMC9987046

[146] B. Yang , H. Bai , Y. Sa , P. Zhu , and P. Liu , “Inhibiting EMT, Stemness and Cell Cycle Involved in Baicalin‐induced Growth Inhibition and Apoptosis in Colorectal Cancer Cells,” Journal of Cancer 11, no. 8 (2020): 2303–2317. 32127957 10.7150/jca.37242 PMC7052934

[147] Y. Xiong , J. Wang , H. Zhu , L. Liu , and Y. Jiang , “Chronic Oxymatrine Treatment Induces Resistance and Epithelial‑Mesenchymal Transition Through Targeting the Long Non‐coding RNA MALAT1 in Colorectal Cancer Cells,” Oncology Reports 39, no. 3 (2018): 967–976. 29328404 10.3892/or.2018.6204 PMC5802036

[148] X. Wang , C. Liu , J. Wang , Y. Fan , Z. Wang , and Y. Wang , “Oxymatrine Inhibits the Migration of human Colorectal Carcinoma RKO Cells via Inhibition of PAI‐1 and the TGF‐beta1/Smad Signaling Pathway,” Oncology Reports 37, no. 2 (2017): 747–753. 27959430 10.3892/or.2016.5292 PMC5355745

[149] S. C. Tsai , W. C. Wu , and J. S. Yang , “Tetrandrine Inhibits Epithelial‐Mesenchymal Transition in IL‐6‐Induced HCT116 Human Colorectal Cancer Cells,” OncoTargets and Therapy 14 (2021): 4523–4536. 34456573 10.2147/OTT.S324552 PMC8387317

[150] S. Ben Hamouda and K. Essafi‐Benkhadir , “Interplay Between Signaling Pathways and Tumor Microenvironment Components: A Paradoxical Role in Colorectal Cancer,” International Journal of Molecular Sciences 24, no. 6 (2023): 5600. 36982677 10.3390/ijms24065600 PMC10057671

[151] Q. Z. Chen , Y. Li , Y. Shao , et al., “TGF‐beta1/PTEN/PI3K Signaling Plays a Critical Role in the Anti‐proliferation Effect of Tetrandrine in human Colon Cancer Cells,” International Journal of Oncology 50, no. 3 (2017): 1011–1021. 28197642 10.3892/ijo.2017.3875

[152] B. Kou , W. Liu , W. Zhao , et al., “Thymoquinone Inhibits Epithelial‐mesenchymal Transition in Prostate Cancer Cells by Negatively Regulating the TGF‐beta/Smad2/3 Signaling Pathway,” Oncology Reports 38, no. 6 (2017): 3592–3598. 29039572 10.3892/or.2017.6012

[153] S. Katta , A. Srivastava , R. L. Thangapazham , et al., “Curcumin‐Gene Expression Response in Hormone Dependent and Independent Metastatic Prostate Cancer Cells,” International Journal of Molecular Sciences 20, no. 19 (2019): 4891. 31581661 10.3390/ijms20194891 PMC6801832

[154] X. Liu , Y.‐S. Piao , and J. T. Arnold , “Transforming Growth Factor β1 Increase of Hydroxysteroid Dehydrogenase Proteins Is Partly Suppressed by Red Clover Isoflavones in human Primary Prostate Cancer‐derived Stromal Cells,” Carcinogenesis 32, no. 11 (2011): 1648–1654. 21914638 10.1093/carcin/bgr206 PMC3218644

[155] N. Dalpatraj , J. Tak , A. Naik , and N. Thakur , “Hesperetin Modulates TGFβ Induced Metastatic Potential of Prostate Cancer Cells by Altering Histone Methylation Marks,” Advances in Cancer Biology—Metastasis 6 (2022): 100077.

[156] M. M. Baruah , A. P. Khandwekar , and N. Sharma , “Quercetin Modulates Wnt Signaling Components in Prostate Cancer Cell Line by Inhibiting Cell Viability, Migration, and Metastases,” Tumour Biology: The Journal of the International Society for Oncodevelopmental Biology and Medicine 37, no. 10 (2016): 14025–14034. 27495232 10.1007/s13277-016-5277-6

[157] B. S. Pollard , M. A. Suckow , W. R. Wolter , et al., “Digitoxin Inhibits Epithelial‐to‐Mesenchymal‐Transition in Hereditary Castration Resistant Prostate Cancer,” Frontiers in Oncology 9 (2019): 630. 31428571 10.3389/fonc.2019.00630 PMC6687970

[158] Y. C. Wen , W. J. Lee , P. Tan , et al., “By Inhibiting Snail Signaling and miR‐23a‐3p, osthole Suppresses the EMT‐mediated Metastatic Ability in Prostate Cancer,” Oncotarget 6, no. 25 (2015): 21120–21136. 26110567 10.18632/oncotarget.4229 PMC4673254

[159] S. Terzioglu‐Usak , M. T. Yildiz , B. Goncu , and N. Ozten‐Kandas , “Achieving the Balance: Biphasic Effects of Genistein on PC‐3 Cells,” Journal of Food Biochemistry 43, no. 8 (2019): e12951. 31368541 10.1111/jfbc.12951

[160] S. Mirzoeva , C. A. Franzen , and J. C. Pelling , “Apigenin Inhibits TGF‐beta‐induced VEGF Expression in human Prostate Carcinoma Cells via a Smad2/3‐ and Src‐dependent Mechanism,” Molecular Carcinogenesis 53, no. 8 (2014): 598–609. 23359392 10.1002/mc.22005

[161] H. J. Ting , G. Deep , A. K. Jain , et al., “Silibinin Prevents Prostate Cancer Cell‐mediated Differentiation of Naïve Fibroblasts Into Cancer‐associated Fibroblast Phenotype by Targeting TGF β2,” Molecular Carcinogenesis 54, no. 9 (2015): 730–741. 24615813 10.1002/mc.22135 PMC4208986

[162] S. Shan , M. Su , H. Wang , et al., “Y‐27632 Targeting ROCK1&2 Modulates Cell Growth, Fibrosis and Epithelial‐mesenchymal Transition in Hyperplastic Prostate by Inhibiting β‐catenin Pathway,” Molecular Biomedicine 5, no. 1 (2024): 52. 39455522 10.1186/s43556-024-00216-9 PMC11511810

[163] S. E. Campbell , B. Rudder , R. B. Phillips , et al., “γ‐Tocotrienol Induces Growth Arrest Through a Novel Pathway With TGFβ2 in Prostate Cancer,” Free Radical Biology and Medicine 50, no. 10 (2011): 1344–1354. 21335085 10.1016/j.freeradbiomed.2011.02.007

[164] X. P. Shi , S. Miao , Y. Wu , et al., “Resveratrol Sensitizes Tamoxifen in Antiestrogen‐resistant Breast Cancer Cells With Epithelial‐mesenchymal Transition Features,” International Journal of Molecular Sciences 14, no. 8 (2013): 15655–15668. 23896596 10.3390/ijms140815655 PMC3759878

[165] Z. Yang , A. Garcia , S. Xu , et al., “Withania Somnifera Root Extract Inhibits Mammary Cancer Metastasis and Epithelial to Mesenchymal Transition,” PLoS ONE 8, no. 9 (2013): e75069. 24069380 10.1371/journal.pone.0075069 PMC3771884

[166] M. M. Al‐Ansari and A. Aboussekhra , “Caffeine Mediates Sustained Inactivation of Breast Cancer‐associated Myofibroblasts via Up‐regulation of Tumor Suppressor Genes,” PLoS ONE 9, no. 3 (2014): e90907. 24595168 10.1371/journal.pone.0090907 PMC3940951

[167] J. Lee , E. R. Hahm , A. I. Marcus , and S. V. Singh , “Withaferin A Inhibits Experimental Epithelial‐mesenchymal Transition in MCF‐10A Cells and Suppresses Vimentin Protein Level in Vivo in Breast Tumors,” Molecular Carcinogenesis 54, no. 6 (2015): 417–429. 24293234 10.1002/mc.22110 PMC4039625

[168] G. M. Adinew , E. Taka , B. Mochona , et al., “Therapeutic Potential of Thymoquinone in Triple‐Negative Breast Cancer Prevention and Progression Through the Modulation of the Tumor Microenvironment,” Nutrients 14, no. 1 (2021): 79. 35010954 10.3390/nu14010079 PMC8746460

[169] N. M. Elsherbiny and M. M. Al‐Gayyar , “Anti‐tumor Activity of Arjunolic Acid Against Ehrlich Ascites Carcinoma Cells in Vivo and in Vitro Through Blocking TGF‐beta Type 1 Receptor,” Biomedicine & Pharmacotherapy 82 (2016): 28–34. 27470335 10.1016/j.biopha.2016.04.046

[170] P. Paramita , B. W. Wardhani , S. I. Wanandi , and M. Louisa , “Curcumin for the Prevention of Epithelial‐Mesenchymal Transition in Endoxifen‐Treated MCF‐7 Breast Cancer Cel,” Asian Pacific Journal of Cancer Prevention 19, no. 5 (2018): 1243–1249. 29801408 10.22034/APJCP.2018.19.5.1243 PMC6031844

[171] J. Lee , “3,3'‐Diindolylmethane Inhibits TNF‐alpha‐ and TGF‐beta‐Induced Epithelial‐Mesenchymal Transition in Breast Cancer Cells,” Nutrition and Cancer 71, no. 6 (2019): 992–1006. 31032639 10.1080/01635581.2019.1577979

[172] C. Allegretta , G. Difonzo , F. Caponio , G. Tamma , and O. Laselva , “Olive Leaf Extract (OLE) as a Novel Antioxidant That Ameliorates the Inflammatory Response in Cystic Fibrosis,” Cells 12, no. 13 (2023): 1764. 37443798 10.3390/cells12131764 PMC10340374

[173] P. Nath , D. Majumder , R. Debnath , M. Debnath , S. Singh Sekhawat , and D. Maiti , “Immunotherapeutic Potential of Ethanolic Olive Leaves Extract (EOLE) and IL‐28B Combination Therapy in ENU Induced Animal Model of Leukemia,” Cytokine 156 (2022): 155913. 35640418 10.1016/j.cyto.2022.155913

[174] Z. Habli , G. Toumieh , M. Fatfat , O. N. Rahal , and H. Gali‐Muhtasib , “Emerging Cytotoxic Alkaloids in the Battle Against Cancer: Overview of Molecular Mechanisms,” Molecules (Basel, Switzerland) 22, no. 2 (2017): 250. 28208712 10.3390/molecules22020250 PMC6155614

[175] E. Azzini , S. I. Peña‐Corona , H. Hernández‐Parra , et al., “Neuroprotective and Anti‐inflammatory Effects of Curcumin in Alzheimer's Disease: Targeting Neuroinflammation Strategies,” Phytotherapy Research 38, no. 6 (2024): 3169–3189. 38616356 10.1002/ptr.8200

[176] E. Azzini , L. Barnaba , M. Mattera , D. Calina , J. Sharifi‐Rad , and W. C. Cho , “Updated Evidence on Raspberries as Functional Foods: Anticancer Bioactivity and Therapeutic Implications,” Food Frontiers 5, no. 6 (2024): 2351–2382.

[177] P. Chaudhary , P. Janmeda , A. O. Docea , et al., “Oxidative Stress, Free Radicals and Antioxidants: Potential Crosstalk in the Pathophysiology of human Diseases,” Frontiers in Chemistry 11 (2023): 1158198. 37234200 10.3389/fchem.2023.1158198 PMC10206224

[178] P. Chaudhary , D. Mitra , P. K. Das Mohapatra , et al., “Camellia Sinensis: Insights on Its Molecular Mechanisms of Action towards Nutraceutical, Anticancer Potential and Other Therapeutic Applications,” Arabian Journal of Chemistry 16, no. 5 (2023): 104680.

[179] A. K. Calgarotto , A. L. Longhini , F. V. Pericole de Souza , et al., “Immunomodulatory Effect of Green Tea Treatment in Combination With Low‐dose Chemotherapy in Elderly Acute Myeloid Leukemia Patients With Myelodysplasia‐related Changes,” Integrative Cancer Therapies 20 (2021): 15347354211002647. 33754891 10.1177/15347354211002647 PMC7995304

[180] G. D'Arena , V. Simeon , L. De Martino , et al., “Regulatory T‐Cell Modulation by Green Tea in Chronic Lymphocytic Leukemia,” International Journal of Immunopathology and Pharmacology 26, no. 1 (2013): 117–125. 23527714 10.1177/039463201302600111

[181] clinicaltrials.gov . Phase II Study of Curcumin vs Placebo for Chemotherapy‐Treated Breast Cancer Patients Undergoing Radiotherapy. Accessed 25 November 2023, https://clinicaltrials.gov/study/NCT01740323

[182] NCT03232138 Cg . Clinical Trial of Lung Cancer Chemoprevention With Sulforaphane in Former Smokers. Accessed 15 July 2024, https://clinicaltrials.gov/study/NCT03232138

[183] H. Wang , Y. Chen , L. Wang , Q. Liu , S. Yang , and C. Wang , “Advancing Herbal Medicine: Enhancing Product Quality and Safety Through Robust Quality Control Practices,” Frontiers in pharmacology 14 (2023): 1265178. 37818188 10.3389/fphar.2023.1265178 PMC10561302

